# Evolution of networks of protein domain organization

**DOI:** 10.1038/s41598-021-90498-8

**Published:** 2021-06-08

**Authors:** M. Fayez Aziz, Gustavo Caetano-Anollés

**Affiliations:** Evolutionary Bioinformatics Laboratory, Department of Crop Sciences, University of Illinois, Urbana, IL 61801 USA

**Keywords:** Computational biology and bioinformatics, Evolution, Molecular biology, Structural biology

## Abstract

Domains are the structural, functional and evolutionary units of proteins. They combine to form multidomain proteins. The evolutionary history of this molecular combinatorics has been studied with phylogenomic methods. Here, we construct networks of domain organization and explore their evolution. A time series of networks revealed two ancient waves of structural novelty arising from ancient ‘*p*-loop’ and ‘winged helix’ domains and a massive ‘big bang’ of domain organization. The evolutionary recruitment of domains was highly modular, hierarchical and ongoing. Domain rearrangements elicited non-random and scale-free network structure. Comparative analyses of preferential attachment, randomness and modularity showed yin-and-yang complementary transition and biphasic patterns along the structural chronology. Remarkably, the evolving networks highlighted a central evolutionary role of cofactor-supporting structures of non-ribosomal peptide synthesis pathways, likely crucial to the early development of the genetic code. Some highly modular domains featured dual response regulation in two-component signal transduction systems with DNA-binding activity linked to transcriptional regulation of responses to environmental change. Interestingly, hub domains across the evolving networks shared the historical role of DNA binding and editing, an ancient protein function in molecular evolution. Our investigation unfolds historical source-sink patterns of evolutionary recruitment that further our understanding of protein architectures and functions.

## Introduction

The biological functions of genes manifest through the proteins or functional RNA molecules they encode. In evolution, novel functions appear when genes produce new genes by duplication, mutation, recombination, fusion and fission, or when genes are generated de novo. Research has attempted to quantitatively describe the origins of these processes of molecular diversification and how they increase molecular complexity over the course of evolution, for instance through pathways of protein domain organization^1,2^. Protein domains are structural and functional units of evolution that make up proteins^3–5^, sometimes in unusually complex arrangements^6,7^. They fold into compact 3-dimensional (3D) atomic structures that arrange alpha-helical and beta-sheet structure elements into tightly packed conformations of the polypeptide chain^8^. The Structural Classification of Proteins (SCOP)^9^ and its extended version SCOPe^10^ are popular taxonomy gold standards of domain structure. SCOP definitions can be used to scan genome sequences for motifs of domains and study how they combine in proteins^6^. In SCOP, the structure of domains exhibiting similar 3D arrangements of secondary structures and thus identical topologies have been classified as folds (F)^9^. Within folds, protein domains whose structure and functional features indicate a common evolutionary origin are further grouped into fold superfamilies (FSF). These FSFs sometimes hold multiple evolutionarily related families, which unify domains with pairwise amino acid identities of more than 30% (Supplementary Fig. S1A). As of March 9, 2021, 276,231 annotated SCOPe domains populate the 175,282 protein structures of the Research Collaboratory for Structural Bioinformatics Protein Data Bank (RCSB-PDB). We note that the cornerstone of the SCOP domain hierarchy is common ancestry, i.e. the existence of shared-and-derived features in domain sequence, structure and function. Homology is also central to many other domain taxonomies, including CATH^11^, CDD^12^, ProDom^13^, Pfam^14^, and the meta-database InterPro^15^. Most databases benefit from machine learning. For example, SCOP and Pfam depend on the identification of conserved regions in protein sequences through sequence alignment and background knowledge, which are then used to build probabilistic hidden Markov models (HMMs) of linear sequence analysis. For example, SCOP uses HMMs of structural recognition to recurrently enrich the database^16^ in a framework that increases alignment-quality and stability of family and superfamily relationships. A similar framework drives the Pfam database but focuses exclusively on sequence information. One difficulty is that not all domains fold into discrete structural entities within the space of possible folds^17^. Some popular domains overlap within a continuum. This ‘gregariousness’ complicates domain classification, demanding the exploration of super-secondary structural motifs as candidate lower-level units of structure, function and evolution^18^.

Domain structures appear repeatedly in the protein molecules, singly or in combination with other domains^7^. More than two-thirds of protein sequences are longer than an average domain length, a vast majority of which are multidomain proteins^19^. A study of protein structures in 749 genomes showed that the lengths of orthologous protein families in Eukarya were almost double the lengths found in Bacteria and Archaea^20^. This variance among lengths results from shorter prokaryotic nondomain sequences that link domains to each other in proteins and have evolved reductively in prokaryotes but not in eukaryotes. The arrangement of domains along the sequence of multimeric proteins is referred to as *‘domain organization.’* Both the structure and organization of domains, which have been collectively termed protein domain *‘architecture’*, are considered far more evolutionarily conserved than protein sequence^7,21–23^. In addition, some domain combinations make up functional units that recur in different protein contexts^24^. They have been termed supradomains (Supplementary Fig. S1B). Thus, domains and supradomains behave as modules, parts that interact more often with each other than with other parts or modules of the system.

Comparative genomic approaches allow to study the modular landscape of domain organization. For example, the evolutionary placement of domains in multiple architectural contexts can be quantified by counting distinct neighbors^25^, domain adjacencies^26^, or consecutive domain triplets^27^ in proteins. These measures of ‘versatility’, ‘promiscuity’ or ‘mobility’ (reuse) of domain building blocks depend on both domain size and abundance. Smaller domains are more likely to be used in multidomain proteins and are therefore more mobile^27,28^, an observation supported by a Menzerath-Altmann’s law of domain organization driven by an economy of scale^29^. Similarly, highly abundant domains appear more versatile, prompting abundance-based normalization of domain versatility measurements when studying intrinsic combinatorial properties and variation across lineages and biological functions^30–32^.

In order to retrace past events in architectural evolution, statements of history (phylogenies) proposed directly from genomic data must be used to build chronologies of first evolutionary appearance of domains and domain architectures. Unfortunately, protein sequence has limited power in deep retrodictive exploration^7,21–23^. Furthermore, while structure is conserved over longer evolutionary timescales, a general metric for global pairwise comparison of structures does not yet exist^33^. Thus, the systematic classification of protein structure has been unable to unify the widely divergent folded structures at any level of abstraction (e.g. FSFs in a ‘galaxy’ of folds^34^), likely because different neighborhoods in protein sequence space contain different structures and functions^35^. Construction of a ‘periodic table’ of idealized structural representations of folds^36^ has not alleviated this difficulty due to an absence of rules of structural transformation that would explain the comparative framework. Numerous efforts to dissect the evolution of domain architectures have recently been reviewed^37^. To overcome limitations and produce global evolutionary views of the protein world, the focus shifted from molecules to proteomes. Trees of proteomes were first reconstructed from a proteomic census of structural domains (beginning with ref.^38^), and were later used to trace character-state changes along their branches to establish possible domain origins^39,40^. This approach, however, was restricted to domain structures and architectures appearing after the common ancestor of the proteomes surveyed in the trees. A much more effective way to create truly global chronologies of the protein world was the reconstruction of phylogenomic trees of domain organization (beginning with ref.^41^). These phylogenies take advantage of powerful serial homologies defined by the proteomic abundance of domains^42^ or architectures^7,43^ defined at F and FSF levels. Phylogenomic trees of domain structures helped uncover the natural history of biocatalysis by tracing chemical mechanisms in enzymatic reactions^44^, analyze the optimization and increase of protein folding speed derived from a flexibility-correlated factor known as contact order (the average relative distance of amino acid contacts in the tertiary structure of proteins)^45^, and study the history of an ‘elementary functionome’ with a bipartite network of elementary functional loop sequences and structural domains of proteins^46^. This last study revealed two initial waves of functional innovation involving founder ‘*p*-loop’ and ‘winged helix’ domain structures, and the emergence of hierarchical modularity and power law behavior in network evolution. Phylogenomic trees of domain architectures and their associated chronologies of molecular accretion showed that architectural diversification evolved through gradual accumulation of domains (singly occurring domains), domain pairs (two different domains), multidomains (numerous domains, with occasional repetition) and domain repeats (domains of one type that are repeated)^7^. The diversification began with a few single-domain architectures earlier in the timeline, followed by an increasing rate of accretion that culminated in a massive “big bang” of domain organization. The accumulation of architectures continued to date but with a decreasing rate^7,46^.

Here, we explore the evolving interactome of protein domain organization. We generate a chronology that captures the historical development of domain and multidomain interactions with a graph theoretical approach^6^ of time-varying (evolving) network structure. The chronology was calibrated with a molecular clock of protein structures, which transforms times of origin of domain architectures into geological timescales of billions of years (Gy)^47^. Five distinct composition- and topology-based ‘operative’ criteria of connectivity defined nodes and links of the evolving networks. This strategy identified connectivity distributions in a series of 169 growing networks, hubs of evolutionary recruitment acting as *donors* and *acceptors*, and structural adaptations of evolving networks to modular, random and scale-free properties. In particular, we discover a pattern of connectivity driven by fusions and fissions, respectively, with densely linked older and younger architectures from the evolutionary timeline sandwiching a period of sparse connectivity. This supports a biphasic or hourglass pattern previously observed in protein evolution^48^ and follows a model of module emergence^49^. We thus reveal remarkable patterns of emergence of hierarchy, modularity and structural cooption in evolving networks.

## Results and discussion

### Construction of evolving networks

**Figure 1:**
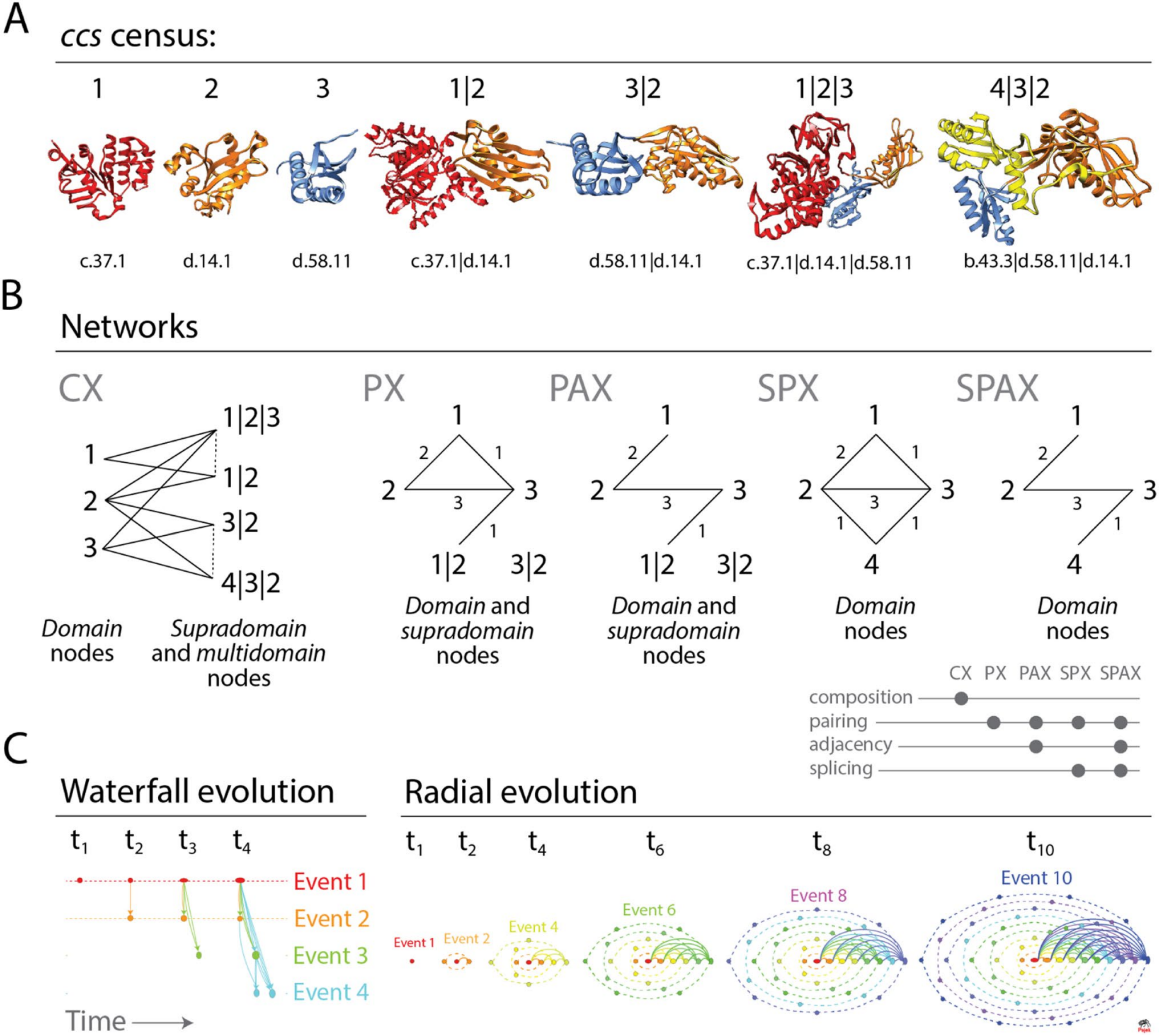
Networks of protein domain organization. (**A**) The genomic census of structural domains and their combinations defines SCOP *concise classification string* (*ccs*) descriptors of domains, supradomains and multidomains that are building blocks of networks. We illustrate the census with a sample from the entire entity set, comprising of 3 domains (1, 2 and 3), 2 supradomains (1|2 and 3|2) and 2 multidomains (1|2|3 and 4|3|2) that are common in dehydratase enzymes and elongation factors. *ccs* identifiers of structural domain constituents defined at fold superfamily (FSF) level are listed below the atomic models visualized in ribbon format with Chimera. (**B**) Five operative criteria for network generation capture the interactions among protein architecture nodes as networks grow in evolution. CX is a partial bipartite network (projection-decomposable) that connects domain nodes to supradomain and multidomain nodes (which can connect to each other; hatched links) when present in multidomain proteins. PX connects domain and supradomain nodes when multidomain proteins are ‘decomposed’ into pairs of architectures, regardless of topological constraints. PAX borrows the PX criterion but respects topological constraints. SPX connects domain nodes spliced from architectures when domain pairs are present in proteins. SPAX connects domain nodes when adjacent domain pairs are present in proteins. (**C**) Chronological development of evolving networks. In ‘waterfall evolution’ layout, time progresses from left to right as ‘discrete events’ of network evolution progressively unfold the appearance of nodes and links (time-directed arrows known as arcs) from top to bottom, colored according to their age. Arc multiplicities describe link cardinality. Source-sink recruitments of architectures are visualized by horizontal and vertical elongations of node symbols, which describe their outdegree and indegree, respectively. As networks grow, the symbols of older nodes widen by outdegree accumulation, while those of younger nodes grow tall by indegree accumulation. In ‘radial evolution’ layout, the time-variant network grows by accumulating nodes in concentric rings (orbitals), each reflecting a time event. We illustrate radial evolution with 6 snapshots of a network growing to a size of 55 nodes as it unfolds from time t_1_ to t_10_. Nodes (n) in orbitals (r) grow at r + 1 rate and only one node per orbital connects to single nodes in each of the other orbitals. Thus, outward links (o) of an orbital are o = t–r–1, where t is the current time. Inward links (i) of an orbital are i = t–o–1 = r. Finally, total links of a network at any time are t(t − 1)/2. The width and height of symbols represent the outdegree and indegree of nodes, respectively. Symbol sizes are shifted by 10 for a better visualization of nodes.

We build a time series of networks of domain organization embedding evolutionary information derived from the sequence and structure of millions of protein sequences encoded in hundreds of genomes. The goal is to unfold the history of how single-domain and multidomain proteins share domain make-up and how recruitment processes shape protein evolution. An ‘entity set’ of domains, supradomains, and multidomains were first extracted from the genomic census of fold structure and domain organization. This set of component parts of proteins, mostly recurrent, defined the nodes of the networks, which were labeled with *concise classification strings* (*ccs*) describing SCOP domain constituents (Fig. 1A). We define supradomains as sub-combinations of domains that appear in the census and are often used as evolutionary building blocks of multidomains. The definition is more inclusive than that of ref^24^.

**Figure 2:**
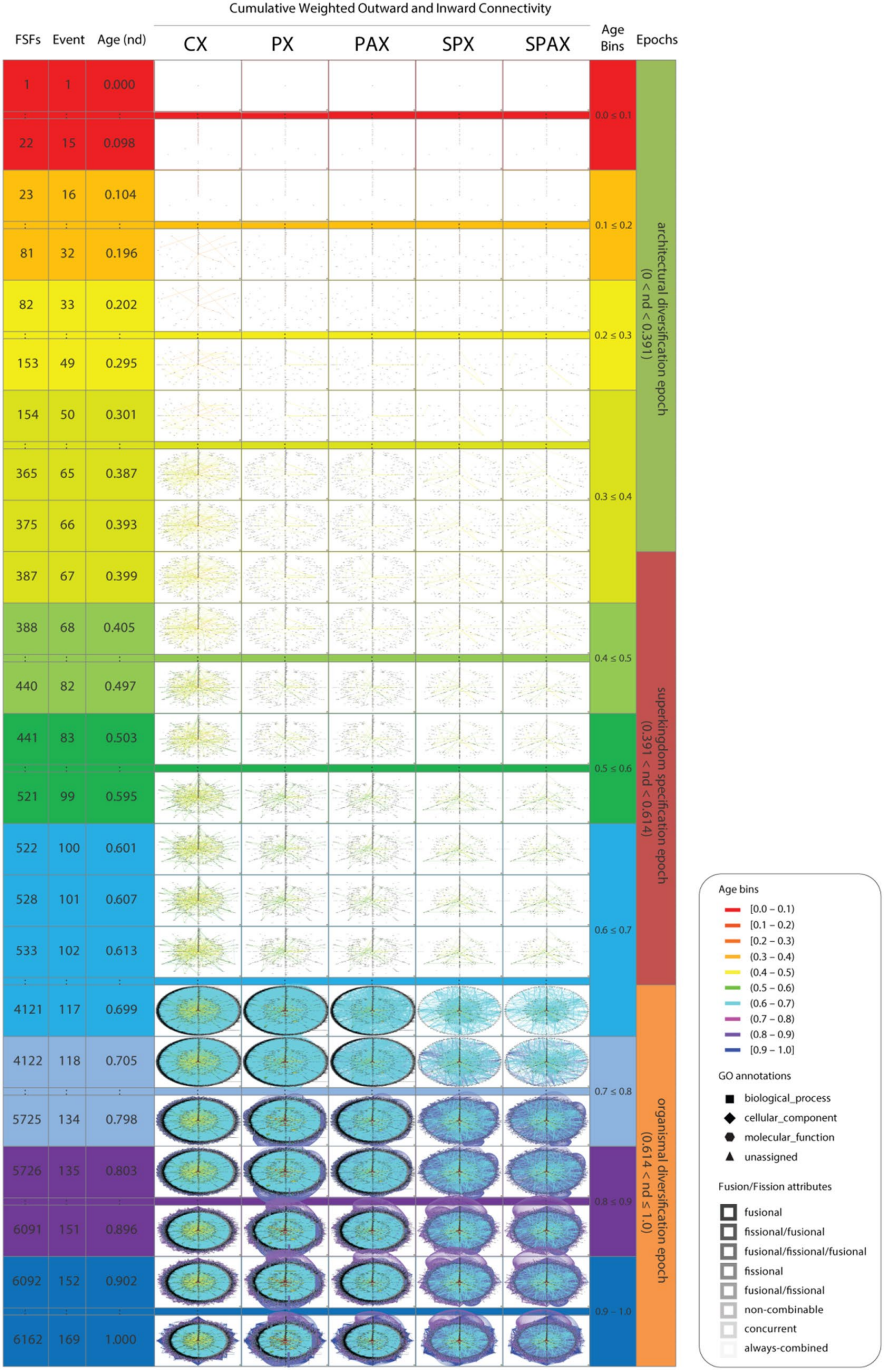
Evolving networks in radial evolution layout. Snapshots of network growth describe the evolution of 6162 domain, supradomain and multidomain architectures or 1643 domains spliced from them. They represent 24 out of 169 time events of the evolutionary timeline, which are indexed with evolutionary age (nd, ranging from 0.0 to 1.0), age bin (one of 10), and one of the 3 epochs of protein evolution ^42^. Age bins were custom RGB color-coded to highlight the flow of time, from top to bottom. The evolving CX, PX, PAX, SPX and SPAX networks reveal the gradual evolutionary accumulation of nodes and links. The sizes of the horizontal and vertical axes of the node symbols depict outward and inward weighted connectivity, respectively, with all weighted degree vectors shifted by 10 for visualization and inclusion of 0-degree nodes. The curved arcs describe recurring interactions between architectures that are accumulating along the successive events of the timeline. Arcs symbolize the flow of time from ancient to recent architectures and are color-coded according to the age of the more recent of the component nodes involved; arcs between contemporary nodes are excluded. Since, in pairwise networks the age of the most recent parent node could be assigned to the arc, the connectivity-defining pairing events are absent in the first (red) and the first and second (red, orange) bins of the PX and SPX and the PAX and SPAX networks, respectively. The angles of multiple arcs emerging from nodes are incremented by 2 to avoid overlap. Node RGB colors represent age. Grey-scale color of node borders depict fusional/fissional properties. Node shapes describe GO categories. High-resolution figures and Pajek network files are available at https://github.com/gcalab/SciRep.

The growing interactions among contemporary architectures are constrained by domain make up and domain arrangement in the protein chain. These evolving interactions were captured with five different operative criteria for timed network generation defined by composition, pairwise occurrence, adjacency, and splicing of domain parts in a protein molecule, where: (1) *composition* describes makeup (component parts) of the molecular whole; (2) *pairwise occurrence* describes appearance of parts in sets of two; (3) *adjacency* refers to their geometrical or spatial arrangement (topology); and (4) *splicing* refers to the rearrangement of parts by operations of joining and excision that decompose structures (Fig. 1B). The Composition Network (CX) linked domain and supradomain to multidomain nodes (in a partially bimodal fashion) when proteins shared compositional makeup. The Pairwise Network (PX) connected domain to supradomain nodes when components occurred in pairs in a protein. The Pairwise Adjacency Network (PAX) connected domain to supradomain nodes when components occurred in pairs that were adjacent. The Spliced Pairwise Network (SPX) linked domain nodes to each other when their pairs were present in domain-spliced proteins. Lastly, the Spliced Pairwise Adjacency Network (SPAX) linked domain nodes to each other when their adjacent pairs were present in the domain-spliced proteins (Fig. 2).

**Figure 3:**
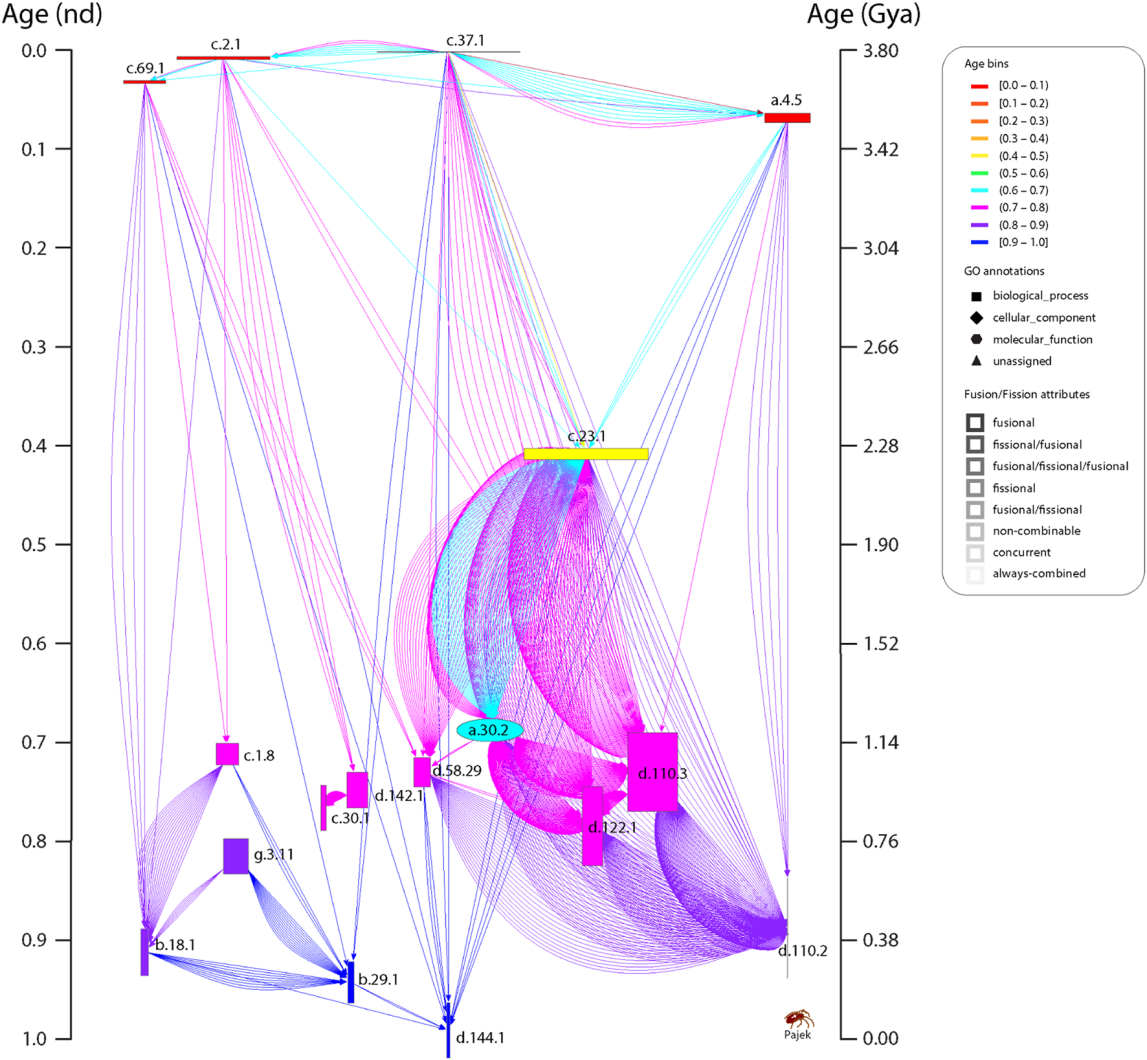
An extant SPX network in waterfall layout describing the evolution of spliced domains with the largest (100th percentile) network connectivity. The SPX network of 1,643 spliced domains was reduced with the restrictive criterion of excluding nodes with combined outdegrees and indegrees ≥ 99% of those of the rest of the nodes. The set of arcs (arched arrows symbolizing flow of time) was also reduced to pairing events between domains in the 100th percentile connectivity and excluded those between contemporary nodes. Nodes are arranged top-down and colored according to age (*nd*) on a relative 0-to-1 scale that describes evolutionary time events. Ages are also time-calibrated with a molecular clock of FSF domains, which uses fossils and microfossils, geochemical, biochemical, and biomarker data^47^. FSF origin is given in billion years ago (Gya). Nodes were labeled with SCOP ccs domain descriptors. To showcase source-and-sink relationships, node symbol sizes were scaled proportional to the weighted outdegree and indegree along the horizontal and vertical axes, respectively. Weighted degrees were scaled as × 2 + 2 to include 0-degree nodes for better visualization. The modular spread of nodes was based on VOS clustering (see methods). Arcs are color-coded according to the age of the more recent of the component nodes involved; no arcs were present in the ancient-most age bin (red) of the timeline. Angles of multiple arcs emerging from nodes are incremented by 2 to avoid overlap.

Finally, we mapped the time or origin (age) of individual architectures onto the nodes of networks built using these five operative criteria (Supplementary Fig. S2). We did so for each of the 169 time-events of the timeline. Network construction has been illustrated with connectivity details of the most ancient domains (Supplementary Fig. S3) and further described in *Sect. 1* of Supplementary Text. Networks showcased time directionality, connectivity distributions, and network layouts:

1. *Time Directionality* Mapping ages onto networks helped follow their evolutionary growth, as nodes and links accumulated over time since the origin of proteins to the present. The timeline of networks imposed a time directionality on network links, making them *arcs* (directed edges with arrows pointing from older to younger nodes) of directed graphs (Fig. 1C). The ages of arcs were borrowed from the youngest of the component nodes involved in a link (Supplementary Fig. S3B).
2. *Degree Distributions* The number of links connected to a node define that node’s ‘degree’. The degree distribution is a ‘composability’ attribute of a network and the entity set represented by its nodes, a design principle describing the inter-relationship of components of a system. In network evolution, the appearance of a new node may trigger establishment of one or more arcs from existing (older) nodes. Furthermore, o*utdegree* describes the number of outward links and i*ndegree* the number of inward links from a node. As the timeline progresses, older nodes gain higher outdegrees as compared to the higher indegrees of recent nodes (Fig. 1C), polarizing the network with arcs depicting ‘arrows of time’ (Supplementary Figs. S2 and S3). The chronological appearance of architectures (domains, supradomains and multidomains) as network connectivity expands along the timeline causes degree to accumulate in the evolving networks (Fig. 2). Multiple interactions of nodes along the timeline diversified connectivity, a feature captured and quantified by weighted degree. Interestingly, box-and-whisker’s plots of weighted outdegree and indegree demonstrate bimodal degree distributions typical of biological systems^49,50^ (Supplementary Fig. S4). The yin-yang patterns of contractions and expansions of architectural innovation are evident from the distributions of modern outdegrees and indegrees (Supplementary Fig. S5). In particular, the cumulative outdegree and indegree scattergrams demonstrate an hourglass (or bimodal) pattern of linkage development unfolding in evolution (Supplementary Fig. S6).
3. *Time Event-based ‘Radial’ and ‘Waterfall’ Layouts* The growth of a network evolving at discrete temporal intervals can be modeled with Discrete Event Simulation (DES) tools^51–53^. Borrowing the DES rationale, we modeled the evolution of directed networks of domain organization with time flowing from one event to another as discrete evolutionary ‘time steps’, typical of a *step function*. The progression of events was visualized with two types of layouts, a vertical representation we coined *‘waterfall’* layout that had nodes arranged top-down by age and a concentric ‘*radial’* representation of growing networks that unfolded time-events of protein evolution from center to periphery (Fig. 1C). Network clusters comprising of hubs and their cohesive neighbors were segregated to improve differentiation along the horizontal axis. The *waterfall* and *radial* layouts made evolutionary recruitment evident as time events progressed downward or outward, respectively (Figs. 2 and 3).

### Early history of modern domain organization

The accumulation of links connecting domain, supradomain and multidomain proteins in evolving CX, PX, PAX, SPX and SPAX networks played back the complicated history of domain recruitments that drive the evolution of domain organization. Figure 2 shows networks in radial layout at representative time-events defining boundaries of the three epochs of the evolving protein world (‘architectural diversification’, ‘superkingdom specification’ and ‘organismal diversification’, *sensu*^7,42^). Networks grew in time and became increasingly complicated tangles, massively expanding after a “big bang” of domain combinations during the organismal diversification epoch. Movies described the evolutionary growth of these networks (Supplementary Video 1).

To illustrate the versatility of the waterfall visualization strategy, we dissected the early origin of proteins with the SPX network. Two major waves of structural innovation arising from ancient ‘*p*-loop’ and ‘winged helix’ domains were observed in the waterfall diagrams of a highly connected (reduced) subnetwork visualization of the SPX network (Fig. 3), matching similar recruitment waves observed in the study of evolutionary networks of elementary functionomes^46^ and metabolites^54^. Waves originated in primordial α/β/α-layered sandwich, β-barrel and helical bundle structures identified in an earlier structural phylogenomic study as part of the most ancient 54 protein domain families^55^. However, most of the connectivity of these major pathways was established during the organismal diversification epoch less than 1.5 Gy ago (*nd* ≥ 0.6) and hence was fully developed relatively recently in evolution. The ‘*p*-loop’ and ‘winged helix’ waves embedded the major gateways of enzymatic recruitment we previously reported for metabolism^54^. The first gateway was mediated by the c.37 P-loop hydrolase fold and originated in the energy interconversion pathways of the purine metabolism subnetwork. The second pathway was mediated by the a.4 winged helix fold and originated in the biosynthesis of cofactors and the metabolic subnetwork of porphyrin and chlorophyll^54,56,57^. The congruent realization of these evolutionary patterns with data sources of different types is remarkable (Supplementary Video 2). It strongly supports the historical statements we propose. Further information can be found in *Sect. 2* of Supplementary Text.

### Network analysis of cooption mechanisms of recruitment

The networks of domains (SPX and SPAX) elicited 161 unique time-events along the evolutionary timeline, out of a total 169 events expected for networks of domains, supradomains and domain combinations (CX, PX and PAX) (Supplementary Tables 1–5). The node and connectivity distributions among the time-event bins of the evolving networks highlight the widespread, growing and recurrent combinatorial recruitment process that incorporates domains and their combinations into protein scaffolds and drives structural evolution (Fig. 2). Indeed, the largest hubs representing the most popular domains in the highly connected SPX subnetwork appeared not only early in evolution but also in the modern protein world (Fig. 3). Similar to the evolution of elementary functions^46^, domain innovation also developed early during the first ~ 1.8 Gy of protein history (Fig. 3). The combinatorial recruitment process however spanned the entire timeline (Supplementary Fig. S2). In terms of origins, the first donor and acceptor composition event occurred in protein evolution with the appearance of a link in the CX network connecting domain c.2.1 to domain combination c.2.1|a.100.1, ~ 3.54 Gya (*nd* = 0.069). The first donor and acceptor pair occurred in the pairwise PX and SPX networks ~ 3.12 Gya (*nd* = 0.179), ~ 0.42 Gy later (Δ*nd* = 0.11). The pairing event involved domains c.37.1 and d.14.1. The first adjacent donor and acceptor pair of the adjacency-based PAX and SPAX networks appeared ~ 2.90 Gya (*nd* = 0.237), ~ 0.22 Gy later (Δ*nd* = 0.06). The adjacently paired nodes were domains c.37.1 and c.23.16. These observations highlight a remarkable tendency of domain organization to gradually but recurrently constrain pairwise occurrences in multidomain proteins. The evolutionary history of donors and acceptors of domain organization is hence associated with a highly optimized process of cooption. To explore this combinatorics, we first dissected the network connectivity with bar plots that describe the chronological accumulation of links along the evolutionary timeline (Supplementary Fig. S7). This made general patterns quantitative and source-sink relationships explicit. Second, we analyzed the per unit donor/acceptor ratio in the evolving networks to highlight pairwise cooption and composability, respectively (Supplementary Fig. S8). Specifically, domain acceptors (represented by network indegree) of SPX increased in number to a global average of 8.63 (± 0.15) sinks per domain in evolution. Domain donors (represented by network outdegree) of SPX reached a higher global average of 9.7 (± 0.56) sources per domain, indicating significant reutilization of relatively ancient domains. In contrast, the average number of donors and acceptors in the evolving CX network plateaued at 3.41 ± 0.34 sources and 3.43 ± 0.05 sinks per domain/multidomain, respectively. This showed uniform source/sink evolutionary rates as proteins acquired higher composability with time. Third, an inferential analysis of cooption-based source-sink relationships maturing at modern times revealed an independence of patterns from the selected network generation criteria (Supplementary Fig. S9). Primarily, the composition events yielding source domains and supradomains were dominant, with the number of events almost doubling in the CX network from the origin to the organismal diversification epoch ~ 1.5 Gya (*nd* = 0.6). However, the pairwise cooption events of the SPX domain network, e.g., doubled in number and reached relatively comparable levels in evolution only after delays of ~ 0.6 Gy (Δ*nd* = 0.15) and ~ 2.1 Gya (*nd* = 0.75), respectively. Moreover, the number of cooption events yielding sink domains in SPX almost tripled by the beginning of the organismal diversification epoch. In contrast, the number of CX sinks reached that level only halfway along that evolutionary epoch. These divergent patterns indicate a frustrated dynamics of network growth. The early adoption of composability of domains and supradomains in multidomains seems to have preceded the pairwise cooption of domains in protein history, leading to the numerous recruitment pathways of the modern protein world. A discussion on the source-sink relationships impacted by domain fusion and fission processes can be found in *Sect. 3* of Supplementary Text.

### Hubs in network evolution

Network hubs are at the heart of network connectivity and could chaperone network evolution^26^. We ranked modern domains and domain combinations of age *nd* = 1 as hubs based on the 99.9^th^ percentile of indegree and outdegree. Hubs were annotated with domain organization attributes, including SCOP domain descriptions, age, fusional/fissional information, and GO terms. We also associated hubs with age ranks reflecting their order of evolutionary appearance in the timeline.

The most notable donor hubs for all networks types were the carrier protein domains e.23.1, a.28.1 and c.69.1, which are involved in Non-Ribosomal Peptide Synthesis (NRPS), whether directly or indirectly through other pathways (Table 1). These domains diversified later in evolution yielding cofactor-binding molecular switches and barrel structures^55^. Ancient NRPS pathways of domain accretion have been associated with a model that not only described stabilization and decoration of membranes by primordial alpha-helical bundles and beta-sheets, but also explained primordial protein synthesis and genetic code specificity chaperoned by ancient forms of aminoacyl-tRNA synthetase (aaRS) catalytic domains and NRPS modules. NRPS even preceded the emergence of the ribosome, acting as scaffold for nucleic acids and the modern translation function. In particular, the PX and PAX networks highlight the central evolutionary role of these novel emerging cofactor structures in the NRPS pathways. Thus, our findings made explicit that our connectivity criteria of generating networks of domain organization were at the cornerstone of the early development of genetic code and supported the evolutionary model of early biochemistry based on phylogenomic information and network structure.

**Table 1:** Domains and domain combinations scoring >  = 99.9th percentiles of 249.916, [63] and {23}, based on combined outdegrees of the five networks at time points 1.0, [0.676] and {0.671}, respectively. The square and curly brackets denote values from the events after and before the big bang, respectively. N/A, not applicable.

Age rank	Label		Node age	Network(s)	Out degree	Fusional/fissional	Description	GO name
388	c.23.1		0.4046243	PX, PAX, SPX	1013, 390, 330	fissional/fusional	CheY-like	Regulation of multicellular organismal development
1	c.37.1		0.0000000	PX, SPX, PAX, SPAX, CX [CX, PX, SPX, PAX, SPAX] {CX, PX, SPX, PAX, SPAX}	607, 380, 376, 314, 271 [109, 97, 74, 64, 64] {34, 32, 26, 23, 23}	fusional	P-loop containing nucleoside triphosphate hydrolases	Positive regulation of reproductive process
2446	a.30.2		0.6820809	PX	578	Fissional/fusional	Homodimeric domain of signal transducing histidine =	Alkene binding
48	e.23.1		0.1445087	PX [PX]	452 [74]	Fusional	Acetyl-CoA synthetase-like	Regulation of primary metabolic process
2	c.2.1		0.0057803	PX [PX, CX, SPX]	427 [117, 80, 66]	Fusional	NAD(P)-binding Rossmann-fold domains	Pyridine-containing compound metabolic process
1518	d.110.3|a.30.2		0.6763006	PX	423	Fissional/fusional	N/A	N/A
283	a.28.1		0.3526012	PX	416	Fusional	ACP-like	Cell periphery
2543	d.110.3&		0.6820809	PX	369	Fissional/fusional	N/A	N/A
858	d.110.2|d.110.3		0.6763006	PX	357	Fissional/fusional	N/A	N/A
187	c.30.1|d.142.1		0.3179191	PX	352	Fissional/fusional	N/A	N/A
8	c.69.1		0.0346821	PX	327	Fusional	Alpha/beta-Hydrolases	Regulation of multicellular organismal development
4777	d.110.3		0.7225434	PX	325	Fissional/fusional	PYP-like sensor domain (PAS domain)	Regulation of cellular macromolecule biosynthetic process
17	c.23.16		0.0809249	PX	315	Fusional	Class I glutamine amidotransferase-like	Positive regulation of oxidative phosphorylation uncoupler activity
4465	d.122.1|c.23.1		0.7109827	PX	291	Fusional/fissional/fusional	N/A	N/A
1599	d.110.3|d.110.2		0.6763006	PX	262	Fissional/fusional	N/A	N/A
443	c.1.33		0.5028902	PX	253	Fusional	EAL domain-like	Cyclic-guanylate-specific phosphodiesterase activity

Domains c.30.1, b.1.1, d.142.1 and g.3.11 (0.723 < *nd* < 0.977) were the most prominent acceptor hubs (Table 2). These structures are integral parts of two-component signal transduction systems that are common in microbes. The highly modular domains feature dual response regulator proteins involved in the two-component signal transduction system comprising of an N-terminal response regulator receiver domain and a variable C-terminal effector domain with DNA-binding activity. These proteins are transcriptional regulators in bacteria and some protozoa, detecting and responding to environmental changes, e.g. nitrogen fixation. These evolving interactions of microbes with the environment mediated by two-component systems have apparently influenced the evolutionary process of cooption. Three acceptor hubs that were significant in PX with indegree > 250 (following behind the 99.9^th^ percentile in other networks) were Nucleotide cyclase (d.58.29), Spermadhesin, CUB domain (b.23.1), and Fibronectin type III (b.1.2) (*nd* = 0.723–0.809). See *Sect. 4* of Supplementary Text for additional donor/acceptor hub information, and *Sect. 5* for cooption events occurring during the ‘big bang’ of domain organization.

**Table 2:** Domains and domain combinations scoring >  = 99.9th percentile of 247.977, [20] and {5}, based on combined in degrees of the five networks at time points 1.0, [0.676] and {0.671}, respectively. The square and curly brackets denote values from the events after and before the big bang, respectively. N/A, not applicable.

Age rank	Label	Node age	Network(s)	In degree	Fusional/fissional	Description	GO name
6044	d.110.2	0.8728324	PX, PAX, SPX	766, 295, 267	fissional	GAF domain-like	PURINE-containing compound catabolic process
4777	d.110.3	0.7225434	PX	735	Fissional/fusional	PYP-like sensor domain (PAS domain)	Regulation of cellular macromolecule biosynthetic process
5529	d.122.1	0.7745665	PX	701	Fissional/fusional	ATPase domain of HSP90 chaperone/DNA topoisomerase =	Nucleic acid metabolic process
5038	a.30.2|d.122.1	0.7341040	PX	550	Fusional/fissional/fusional	N/A	N/A
5101	c.43.1	0.7398844	PX	445	Fissional/fusional	CoA-dependent acyltransferases	Monocarboxylic acid catabolic process
5664	d.110.3|a.30.2|d.122.1	0.7919075	PX	439	Fusional/fissional	N/A	N/A
6150	c.43.1&	0.9768786	PX	432	Fusional/fissional	N/A	N/A
5304	c.30.1	0.7572255	PX	375	Fissional/fusional	PreATP-grasp domain	Pyrimidine-containing compound biosynthetic process
6148	b.1.1	0.9768786	PX	370	Fissional	Immunoglobulin	Regulation of mesoderm development
4848	e.23.1|a.28.1	0.7225434	PX	367	Fusional/fissional	N/A	N/A
5095	d.142.1	0.7398844	PX	359	Fissional/fusional	Glutathione synthetase ATP-binding domain-like	Pyrimidine-containing compound biosynthetic process
5731	g.3.11	0.8034682	PX	317	Fissional/fusional	EGF/Laminin	Positive regulation of receptor activity
4118	c.43.1&|e.23.1|a.28.1	0.6994219	PX	287	Fusional/fissional/fusional	N/A	N/A
4758	d.58.29	0.7225434	PX	272	Fissional/fusional	Nucleotide cyclase	Regulation of primary metabolic process
5521	d.110.3|d.58.29	0.7745665	PX	266	Fusional/fissional	N/A	N/A
4855	c.43.1&|e.23.1|a.28.1|c.43.1&	0.7225434	PX	265	Fusional/fissional	N/A	N/A
4763	a.30.2|d.122.1|c.23.1	0.7225434	PX	263	Fusional/fissional/fusional	N/A	N/A
5768	b.23.1	0.8092486	PX	261	Fissional/fusional	Spermadhesin, CUB domain	Regulation of anatomical structure size
5759	b.1.2	0.8092486	PX	259	Fissional/fusional	Fibronectin type III	Regulation of CD4-positive, alpha–beta T cell activation
2886	c.43.1|e.23.1|a.28.1	0.6878613	PX	258	Fusional/fissional/fusional	N/A	N/A
[1620]	c.43.1&|e.23.1	0.6763006	PX	28	Fusional/fissional/fusional	N/A	N/A
[1223]	d.142.1|c.24.1	0.6763006	PX	25	Fusional/fissional/fusional	N/A	N/A
[1311]	a.28.1|c.43.1&	0.6763006	PX	25	Fusional/fissional/fusional	N/A	N/A
[1032]	e.23.1|a.28.1|c.43.1&|e.23.1	0.6763006	PX	23	Fusional/fissional/fusional	N/A	N/A
[283]	a.28.1	0.3526012	PX, SPX, PAX, SPAX	22, 22, 21, 21	Fusional	ACP-like	Cell periphery
[1556]	d.142.1|a.92.1|c.30.1|d.142.1|c.24.1	0.6763006	PX	22	Fusional/fissional/fusional	N/A	N/A
[1085]	e.23.1|a.28.1|c.43.1&|e.23.1|a.28.1	0.6763006	PX	21	Fusional/fissional/fusional	N/A	N/A
{672}	a.4.1	0.6647399	PX, CX	8, 7	Fissional/fusional	Homeodomain-like	Regulation of epithelial cell differentiation involved in kidney development
{324}	c.73.1	0.3641618	PX	6	Fissional/fusional	Carbamate kinase-like	Heterocycle metabolic process
{460}	c.73.1|d.58.18&|c.2.1|d.81.1	0.5202312	CX	5	Fusional/fissional	N/A	N/A
{734}	b.113.1|a.156.1|g.39.1|c.37.1	0.6705202	CX	5	Fusional/fissional	N/A	N/A
{13}	c.2.1|a.100.1	0.0693642	PX	5	Fusional/fissional/fusional	N/A	N/A
{58}	d.14.1	0.1791908	PX, SPX	5, 5	Fusional	Ribosomal protein S5 domain 2-like	NUCLEIC acid phosphodiester bond hydrolysis
{270}	g.39.1	0.3468208	PX	5	Fissional/fusional	Glucocorticoid receptor-like (DNA-binding domain)	Fibroblast growth factor receptor signaling pathway involved in ureteric bud formation
{388}	c.23.1	0.4046243	PX	5	Fissional/fusional	CheY-like	Regulation of multicellular organismal development

### Emergence of preferential attachment in network evolution

Genomic-centric processes such as duplication, recombination, fusion and fission shape patterns of molecular complexity^2^. Many of these patterns can be explained with large ‘scale-free’ networks that grow by following the preferential attachment principle^58^. These self-organizing and highly inhomogeneous networks attach links to highly connected hub-like nodes in a ‘rich-get-richer’ fashion, lacking a characteristic scale, irrespective of the properties of individual nodes or systems^59^. This pattern of network expansion, which is remarkably popular in biology^60^, is sharply distinct from that of the Erdős–Rényi random network model^61,62^. In a scale-free network, the probability *P*(*k*) of nodes connecting with neighboring *k* nodes (i.e. the ratio of nodes with *k* links) decays as a power law, *P*(*k*) ~ *k*^–γ^, with γ defined as the exponent of power law decay. The frequency distributions of node-connectivity in biomolecular networks have γ typically ranging 2.1–2.4^63^. Thus, scale-free properties drive degree distributions with heavy tails, where very few nodes have high degree values.

**Figure 4:**
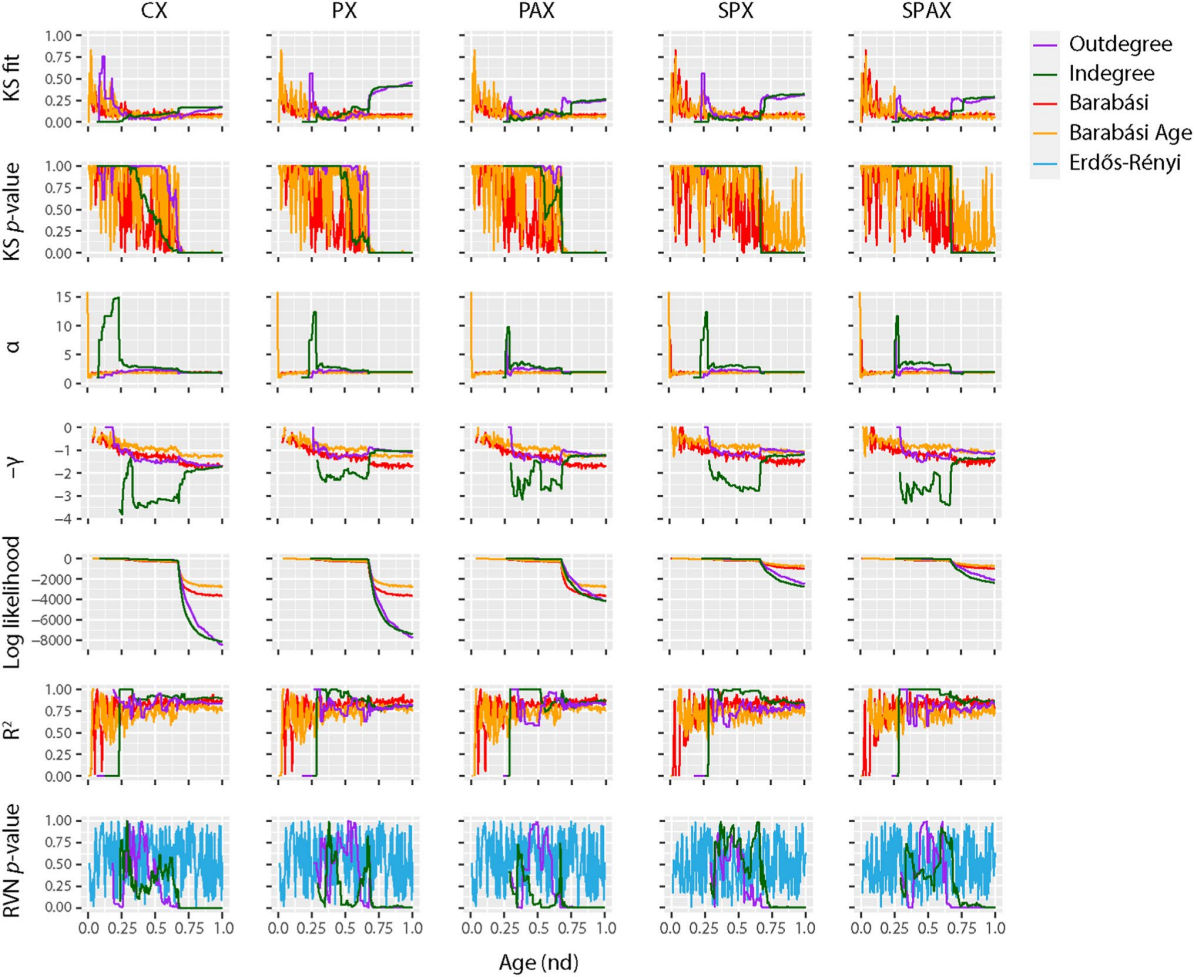
Statistical descriptors of power law and random behavior. Six indicators of preferential attachment were studied along the evolutionary timeline to explore processes of network growth, with network age (*nd*) indicated on a relative 0-to-1 scale. Outdegree and indegree connections were cumulative and weighted in evolving networks. Barabási (red) and Barabási-Age (orange) networks were included as control sets. The Barabási model specifies the probability of preference of an old node as P_i_ ~ k_i_^α^ while the Barabási-Age model grants heavier power law properties to older nodes (exhibiting smaller *nd*) with P_i_ ~ (k_i_^α^)(l_i_^β^), where k_i_ is the indegree of node i of the current event, α is the preferential attachment exponent (α = 1 for linear preferential attachment), l_i_ is the age of node i, i.e. the number of events elapsed since the node was added, with maximum number measured by the ‘aging.bin’ parameter, and β is the aging exponent (β = 1 for linear increases in probability of preference of an older node with high l_i_). Power law indices include: (1) the KS fit statistic that compares the input degree data distribution with the fitted power law distribution (smaller scores denote better fit); (2) the KS *p* value, which rejects the null hypothesis that degree data was drawn from the fitted power-law distribution when less than α = 0.05; (3) the exponent of the fitted power-law distribution (α); (4) the slope of power-law linear regression model (γ); (5) the log-likelihood of the fitted parameters; and (6) the coefficient of determination (R^2^) that measures the percentage of degree data that fits the linear model. The randomness of the evolving networks was quantified by the *p* value of an approximated beta distribution from the rank version of von Neumann's Ratio Test for Randomness^89^ (RVN_*p* value_). The alternate hypothesis was non-randomness. Comparative graphs of strictly random Erdős–Rényi control networks of corresponding sizes at the given time-events were also plotted. Lower KS fit, higher KS *p* value, higher α, lower −γ and near-zero likelihood, given lower RVN_*p* value_, support power law behavior.

Our statistical analyses of the featured indegree distributions along the timeline of growing networks uncovered interesting patterns of power law dynamics (Fig. 4). The scale-free patterns were established early on in protein evolution, primarily evident in the CX composition network. These patterns were remarkably divergent from evolving networks connected at random (RVN *p* value > 0.05). While power law behavior generally declined as the networks evolved (*KS p-value* < 0.05, *α* < 2.5), it somewhat sustained after the ‘big bang’ but only in CX and not in the pairwise networks (*KS fit* and γ closer to 0 and 2 in CX, respectively). A *log* linear regression model of CX produced the highest absolute value for γ of 3.81 among the five networks, which was achieved early along the evolutionary timeline (*nd* ~ 0.25). This value of γ was much higher than values reported for metabolic networks (γ ~ 2.2)^60^. Remarkably, the γ was maintained at ~ 3 before and after the ‘big bang’, while remaining at ~ 2 until modern times with a minimum value of 1.7. The other four networks generated primarily with the pairwise criterion apparently deviated from the power-law behavior, especially after the ‘big bang’. For instance, the γ of PX and PAX peaked at 2.4 (*nd* ~ 0.35) and 3.2 (*nd* ~ 0.38), respectively, slightly later than CX. We also noted a transition in γ from 2.1 in PX and 2.7 in PAX prior to the ‘big bang’ to 1.6 in both after the big bang, plateauing at ~ 1 until the present. In the SPX and SPAX networks, γ reached a peak even later in time than PX and PAX with values of 2.8 (*nd* ~ 0.54) and 3.4 (*nd* ~ 0.66), respectively. These values transitioned from 2.4 in SPX and 2.8 in SPAX from before the big bang to 1.6 and 1.7 after the big bang, respectively, plateauing at ~ 1 in both the networks. As expected, the average γ based on less representative outdegree of each of the five networks remained low (1 ± 0.05).

We noticed biphasic patterns when -γ was plotted over network connectivity, with two minima at *nd* ~ 0.37 and ~ 0.67. Moreover, the scale-free tendency of adjacency networks seemed comparatively higher than that of networks lacking the adjacency restriction. For instance, the average values of γ for the PAX and SPAX networks (1.87 ± 0.06 and 2.13 ± 0.07, respectively) were relatively higher than those for the corresponding parent PX and SPX networks (1.61 ± 0.05 and 1.89 ± 0.06, respectively). This suggests that proximity in amino acid sequence plays a major role in rendering the power-law behavior of evolving networks of domain organization. Overall, the average γ of CX (2.56 ± 0.06) remained the highest along the evolutionary timeline, indicating that composition strongly elicits the preferential attachment property. A complementary transition from random to non-random behavior (RVN *p* value: 1 → 0) in ancient networks (*nd* ~ 0.3) implies deviation from randomness as biological networks evolve. Remarkably, this transition event coincides with the origin of a processive ribosome. Such biphasic patterns are common in biology and have explained the emergence of biological modules^49^ in metabolic networks of *Escherichia coli*^50^, networks of elementary functionomes^46^, and molecular ancestry networks of enzymes^64^. *Section 6* of Supplementary Text further discusses *scale-freeness* and *randomness* of networks.

### Emergence of hierarchical modularity

**Figure 5:**
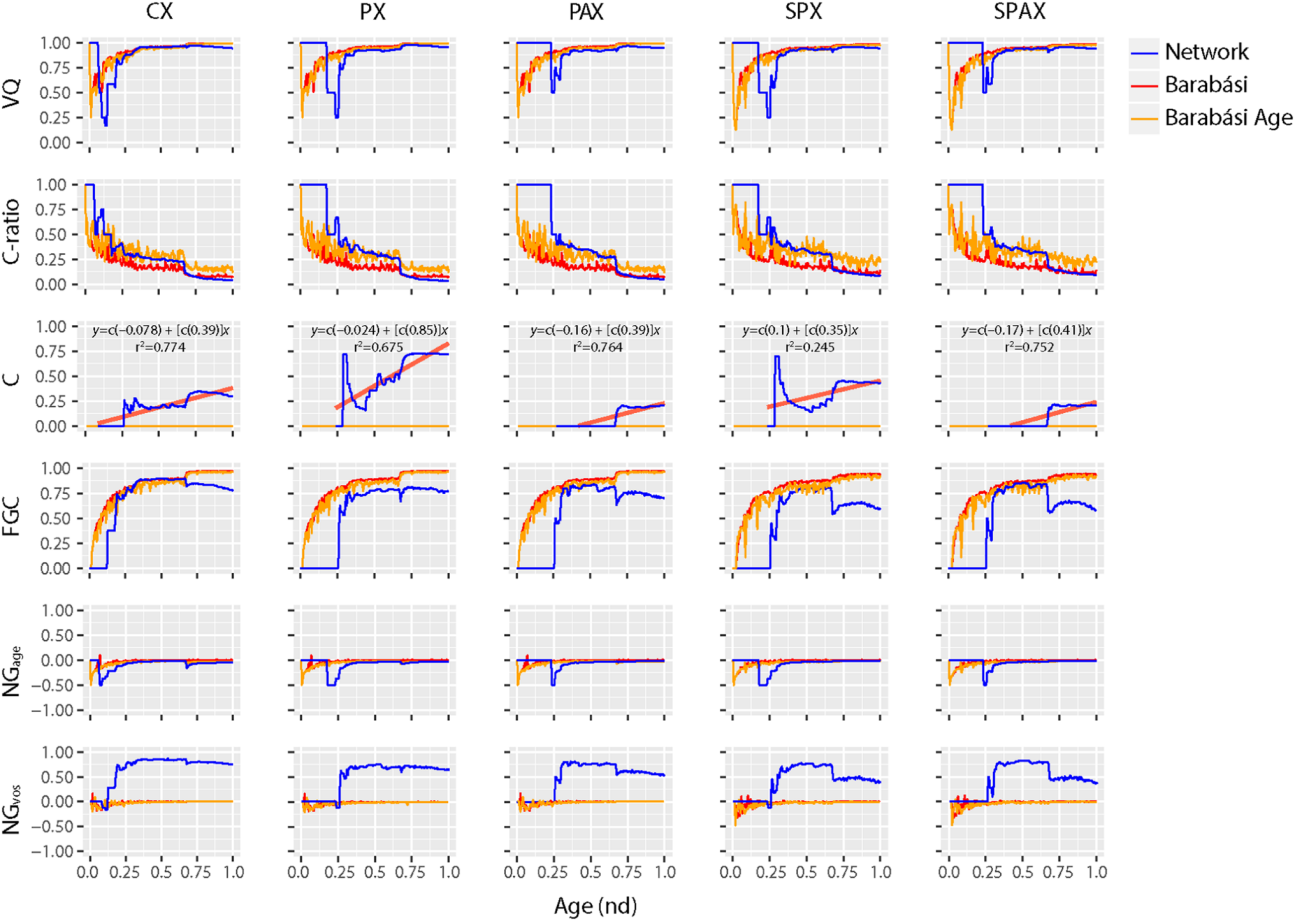
Network modularity. Six indicators of modularity were studied along the evolutionary timeline to explore the evolution of network structure, with network age (*nd*) indicated on a relative 0-to-1 scale. Modularity indices include the VOS Quality (VQ) index, the Clustering ratio (C-ratio), the average Clustering Coefficient (C), the Fast-Greedy Community (FGC) index, and the Newman-Girvan index defined by age (NG_age_) or VOS clustering (NG_VOS_). Modularity calculations required cumulative, undirected, and weighted connectivity input. The Barabási (red) and Barabási-Age (orange) models (see caption of Fig. 4) were included as control sets. The regressions of C with age (nd) are shown as linear models (red lines) for each network together with supporting determination coefficients (R^2^).

Modular networks embed sets of communities (closely-knit modules) that establish links preferentially within themselves and do so sparsely with the rest^65^. Network modularity usually offsets the power-law behavior of biological networks by distributing node degrees within communities^66–68^. However, both scale-free properties and modular structure may co-exist in a network when modules coalesce hierarchically^60^. A primary index of modularity is the *average clustering coefficient* (*C*), defined as a node-averaged ratio of triangles (graph cycles of length 3) to triads (the connected graph triples) of the network, not taking into account the weights or direction of the node-links^60,69,70^ (Fig. 5). The adjacency PAX and SPAX networks both showed the lowest *C* (averaged over *nd*) with a value of 0.09 ± 0.009. The composition CX network had a relatively higher *C* of 0.2 ± 0.009. However, the non-adjacency pairwise PX and SPX networks had the highest *C* values of 0.5 ± 0.02 and 0.32 ± 0.014, respectively. These values were still lower than those reported for metabolic networks (*C* =  ~ 0.6)^60,68,71^. Hence, the networks supposedly evolved more random smaller modules connected by various inter-modular links, rather than stronger larger modules with few interconnections. Also, the evolution of modular structure appeared better consolidated by pairwise (PX and SPX) and to a lesser degree composability (CX) constraints rather than by adjacency (PAX and SPAX). Comparing patterns of modularity of evolving networks to those of randomness (given by RVN_*p* value_) indicated complementary transitions between the two behaviors over the evolutionary timeline (Figs. 4 and 5).

**Figure 6:**
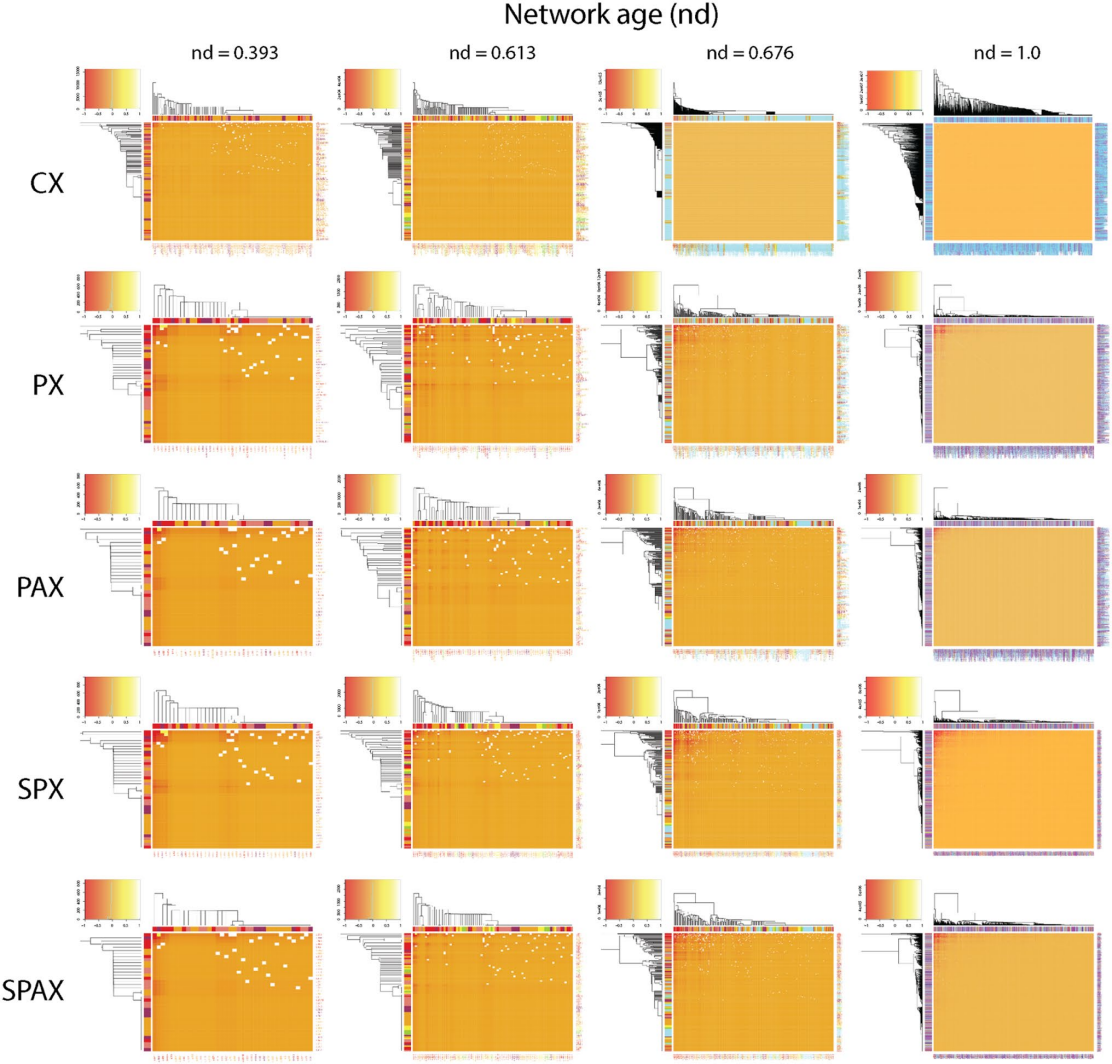
Evolution of modularity and hierarchical organization of networks over select events of the evolutionary timeline. NG_age_ pairwise modularity values^65^, scaled by log10 of network-wide absolute modularity values, were used as input for the calculation of Euclidean distance matrices^87^, which were visualized as heatmaps. Heatmap tiles represent modular strength between any two architectures relative to the respective strength of their linkages to other architectures of the network. The embedded dendrograms that define the order of rows and columns of the heatmaps were generated by hierarchical clustering of the distance matrices with the Ward’s minimum variance method^88^. The height of dendrograms represents dissimilarity between clusters while the clades show grouping rearrangements of architectures. The top-left insets depict frequency histograms of the heatmap modularity values scaled from − 1 to 1 (i.e. disassortative to assortative). The four panels describe growth of each evolving network (left-to-right). Network age corresponds to the middle approximate boundaries of the three evolutionary epochs of the protein world (Supplementary Fig. S2), i.e., end of ‘architectural diversification’ (*nd* = 0.393), end of ‘superkingdom specification’ (*nd* = 0.613), onset of the ‘big bang’ of domain organization at the start of ‘organismal diversification’ (*nd* = 0.676); and the present (*nd* = 1). Nodes were age-sorted ascendingly within clusters and labelled using standard SCOP nomenclature^9^. In the case of SPX and SPAX, nodes correspond to 1643 domains mapped to the entity set of 6162 architectures. The color-coding of bands and labels identifies the age of architectures (Supplementary Fig. S2). The relatively 'flatter' heatmap and 'skewed' dendrogram patterns of CX (typically at *nd* = 0.667 and *nd* = 1.000) are an artifact of unweighted distance matrices of CX, which contrast with the weighted ones of pairwise criterion-based networks. The most prominent clades correspond to the modules of the most ancient domain structures harboring the two major waves of architectural innovation. We also generated heatmaps of power-law control networks of corresponding sizes at the given time-events (Supplementary Fig. S10). When compared to the pairwise networks, the combined heatmap and dendrogram patterns of CX suggest a hidden switch from scale-freeness to modular behavior, eventually giving rise to hierarchical modularity with visible emergence of modules within modules.

In order to dissect the modular behavior of evolving networks, we studied the regression patterns of *C* against network size N and evolutionary age *nd*. For typical scale-free models, *C* declines sharply with increasing N (*C* ~ N^-coefficient^), while the coefficients are as high as 0.75^72^. Instead, highly modular networks are typically independent of N^60^. In our networks, *C* regressed by N with very low coefficients (CX, 0.000036; PX, 0.00007; PAX, 0.000035; SPX, 0.00016; SPAX, 0.00016). In contrast, the regression of *C* with age (*C* ~ *nd*^-coefficient^) produced significantly higher coefficients (CX, 0.39; PX, 0.85; PAX, 0.39; SPX, 0.35; SPAX, 0.41) (Fig. 5). As expected^73^, the reference power-law (Barabási) networks that were used as control showed a *C* of zero. Our data strongly suggests the existence of a highly modular structure that is independent of network growth but is strongly constrained by history, especially when considering the pairwise interactions of the PX network. The rise of the modularity index with emerging power-law degree distribution during certain periods of network evolution indicated a parallel formation of complex hierarchical module clusters with scale-free properties, not distinct from those present in metabolic networks^60^. Our networks of domain organization showed a slight lag between an onset of scale-free organization (measured with KS fit and γ indegree statistics) and a delayed emergence of modular behavior (measured with *C*), occurring during early protein evolution. This was followed by intermittent periods of hierarchical modularity spanning across the middle of the evolutionary timeline. Remarkably, the evolving networks showed again a prominent biphasic pattern of hierarchical modularity involving two peaks of modularity (higher statistic *C*) coinciding with increased power-law behavior (valleys of KS fit and -γ curves), at *nd* ~ 0.37 and *nd* ~ 0.67, respectively (Figs. 4 and 5). The modularity heatmaps and dendrograms of select phases of network evolution confirm these biphasic patterns (Fig. 6), which were markedly distinct from the long-tailed clustering patterns of preferential attachment (Supplementary Fig. S10). As identified earlier^46^, the timing of this switch coincides with the early development of genetic code specificity in the emerging ribosomal aaRS catalytic domains, which was facilitated by the OB-fold structure^74^. These counteracting and delicately balanced trends of modularity and preferential attachment suggest that the emergence of scale-free behavior of the partial bipartite CX network must have impacted the hierarchical modular structure of the modern pairwise networks of domain organization (PX, PAX, SPX, SPAX) (Supplementary Video 3). A detailed account of our testing and verification of this conjecture is explained in Sect. 7 of Supplementary Text.

## Conclusions

We here present for the first time an evolutionary chronology of networks of domain organization. Tracing the time events of origination of protein domain architectures in these growing networks revealed major evolutionary pathways of molecular recruitment of domains and functions. Two prominent ancestral waves of structural novelty involved ancient domain innovations and founder ‘*p*-loop’ and ‘winged helix’ domain structures. We found that evolutionary recruitment in proteins is ongoing and highly modular. Remarkably, the networks highlighted the role of cofactor-supporting structures of NRPS pathways, which were backbone to the early evolution of the genetic code. The evolving domain rearrangements featured multitier evolutionary episodes of scale-free network structure, hierarchy and modular behavior. Remarkably, our analyses support biphasic patterns of diversification and module emergence that we have observed earlier^46,49^. In an initial phase, at the cusp of architectural diversification, the modular components of emerging domain organization associated through weak linkages of recruitment. The second phase was massive and prolonged, with a multitude of modules appearing after the ‘big bang’ of the protein world, supporting the onset of organismal diversification. Such biphasic patterns are prevalent in biology and impact size, dipeptide makeup, and loop-mediated flexibility of proteins, possibly due to their intrinsic disorder^45,74^. We propose that biphasic patterns in evolving networks are integral to module emergence in biological history. We prompt further study of their structure and origin.

## Methods

### Experimental design

#### Phylogenomic analysis of the entity set of protein domain architectures

We explore the evolution of networks describing how structural domains combine and split to form single domain and multidomain proteins, i.e. the domain organization of proteins. The definition of protein domain structures followed the FSF level of SCOP version 1.75^9^ (Fig. 1). Domain interactions were studied along an evolutionary timeline of structural and architectural innovation directly derived from a phylogenomic tree of architectures reconstructed from an HMM-based census of structural domain organization of 1,730 FSF structures from ~ 3 million protein sequences encoded in 749 genomes of 52 archaeal, 478 bacterial and 219 eukaryal organisms (dataset A749)^20^ (Supplementary Fig. S1). The percentage of proteins with structural assignments was 62.2 ± 0.09(SD)% (see Table S1 and discussion in ref.^20^). The tree was generated using maximum parsimony as the optimality criterion in PAUP* following the parsimony ratchet search strategy described in ref.^7^. Data matrix and tree files are provided as Supplementary Files 1–3 at https://github.com/gcalab/SciRep. The phylogeny represents reconstruction of the “natural history” of proteins that is supported by a model of protein structural growth^75^ and is carefully indexed with various evolutionary epochs of the protein world^7^.

#### Calculation of the ages of domain organization

The ages of domains and domain combinations were calculated as node distance (*nd*) values, which were derived directly from the rooted phylogenomic tree of protein domain organization^7^. *nd* values describe relative ages (in a relative 0–1 scale) of first appearances of 6,162 domains and domain combinations (multidomains) defined at SCOP FSF level (the extant ‘entity set’ sampled by our study; Fig. 2) Collectively, ages defined an evolutionary timeline embodying architectural transformations and molecular transitions mediated by fusion and fission processes in the form of 169 unique ‘time events’ (age groups or time slivers) (Supplementary Fig. S2). A Python script was used to count the number of nodes from the root (base) of the tree to each leaf node and present the distance matrix of nodes in a relative zero-to-one scale^46^. The script utilized the high imbalance of phylogenomic trees as a fundamental feature to derive the relative ages of domain organization^7^. The tree imbalance resulted from the accumulation of structures and their combinations in proteins and proteomes and not from node density, thus representing a true evolutionary process^47^.

The timeline was calibrated with a molecular clock of FSF structures (t = –3.831*nd* + 3.628) used to calculate geological age in Gy through calibration points of FSF domains associated with microfossil, fossil and biogeochemical evidence, biomarkers, and first-appearance of clade-specific domains^47^. The *RSCB–PDB* count was determined by following the hyperlink associated to the number of entries or structures (which is updated weekly) and selecting “Customizable Table” from the ’Reports’ menu above the results section. Subsequently, SCOP, CATH, and PFAM ID options were selected as domain information under the ’Domain Details’ section and domain counts data were exported as a comma separated value (.csv) file report. Supplementary Tables 1–5 provide an exhaustive summary of various connectivity categories of evolving networks based on this ‘entity set’ of domain organization. The extraction pipeline of SPX/SPAX domain units from the original data set can be found in Supplementary Table 6.

#### Indexing domain attributes

Domain ages and assignment of fusional/fissional properties followed ref.^7^. SCOP *concise classification strings* (*ccs*) of domain descriptions^9^ were downloaded from http://scop.mrc-lmb.cam.ac.uk/scop/parse/index.html for SCOP version 1.75 as the file dir_des_scop_txt_1_75.txt. Available descriptions for 2,223 single domains were obtained from SCOP unique identifiers (sunID). The Gene Ontology (GO) specifications were recorded from the Superfamily Database (SUPFAM) available at http://supfam.cs.bris.ac.uk/SUPERFAMILY/GO.html. High-coverage domain-centric GO annotations that were supported only by all UniProts (including multidomain UniProts) were downloaded as the file Domain2GO_supported_only_by_all.txt. High-quality truly domain-centric GO annotations that were supported by both single domain UniProts and all UniProts (including multidomain UniProts) were downloaded as the file Domain2GO_supported_by_both.txt. We reported only the GO annotations ‘by all’ to capture higher coverage. Also, the GO terms were reported only for the 2223 single domains with descriptions available. Specialized GO annotations from two levels of hierarchy downstream were taken from files Domain2GO-Hie-Dist1.csv and Domain2GO-Hie-Dist2.csv. Structural domains functional ontology (SDFO) that mapped information from a theoretic analysis of Domain2GO annotation profiles were reported from the file SDFO.txt.

#### Network construction, visualization and analysis

Mathematical definitions for construction of networks can be found in Supplementary Materials and Methods. The social network analysis tool Pajek^76^ and the statistical test bench R’s *igraph* package^77^ were used to visualize and analyze the networks, respectively. The collective impact of events was made explicit by Pajek’s Visualization of Similarity (VOS) clustering method^78,79^. VOS helped reveal communities and design layouts of networks with nodes separated into network modules, where high *modularity indices* ranged from 94–95%. Number of clusters varied over networks (CX, 691; PX, 3,886; PAX, 4,126; SPX, 607; SPAX, 620). Network clusters were visually compacted to hubs and their cohesive neighbors with the energy-optimizing Kamada-Kawai ‘separate components’ algorithm^80^. Pajek allowed to proportionally reduce the size of highly connected nodes by some scaling factor for optimally uncluttered visualization. Waterfall and radial network layouts were designed with node-size scaled down by factors of 0.1 and 0.25, respectively. R packages equipped with specialized code constructs to draw graphs and derive statistics were used to analyze network properties^81,82^. We also used Pre-Hypertext Processing language (PHP) to write custom scripts that generated radial visualizations of the networks and helped conduct housekeeping data management^83^. The PHP scripts were executed in the command line. Results of these scripts were input into Pajek’s and R’s analytical procedures. We used the open-source software ImageMagick (www.imagemagick.org) for batch conversion, captioning, and appending of network images (to represent legends and scales). A detailed description of partition and data files, list of network data analysis functions, charting and graphing procedures, methods to generate power law statistics, modularity indices and randomness checks, and the method pipeline used to achieve waterfall diagrams can be found in Supplementary Materials and Methods.

### Statistical analysis

#### Scale-free network behavior

Linear regression models of *P*(*k*) given *k* (i.e. the probability of having *k-*neighbors) were used to derive the γ coefficient of the power law distribution and the determination coefficient, R^2^. The value of γ represents an absolute slope of the log linear model of *P*(*k*) versus *k*. The slope is usually ≤ 0. γ >> 1 indicates strong tendency towards preferential attachment. R^2^ indicates the percentage of data that fits the linear model. High values of both γ and R^2^ suggest strong scale-free behavior. Additional power law statistics were calculated as: (1) the exponent of the fitted power law distribution, α, with an assumption that P(*X* = *x*) is proportional to x^–α^; (2) KS fit statistic to compare the input degree distribution with that of fitted power-law; and (3) the KS *p*-value of a statistical test, with the null hypothesis that data is being drawn from a power law distribution^84,85^. α >> 1, 0 < KS fit scores << 1, and KS *p*-values ≥ 0.05 suggest that degree data was derived from a fitted power law distribution. Maximum log likelihood of the fitted scale-free parameters was also determined. Control networks were included for reference that were generated with ‘Barabási’ methods^58^ of the *igraph* package from R^77^. These controls simulated basic and extended age-dependent power law graph models given varying sizes of the evolving networks.

#### Network modularity

We investigated modularity using six indices: (1) The *VOS Quality index* (*VQ*) was determined using the Pajek VOS algorithm by considering the number or weights of the links (arcs) between the nodes as similarities. Clusters or communities that were deemed ‘similar’ were iteratively drawn closer to each other until a final layout was achieved with least crossings and closest clusters. The quality index *VQ* was thus calculated for this final layout as ∑_i=1 c, j=i+1 c_ (e_ij _− a_i_^2^), where c is the number of communities; e_ij_ is the fraction of edges with one node v in the community i (c_i_) and the other node w in the community j (c_j_), defined as ∑_vw_ (A_vw_/2 m) where v ϵ c_i_, w ϵ c_j_, m is the sum of weights in the graph and A_vw_ is the weighted value or 0, indicating presence or absence of edge between nodes v and w in the adjacency matrix A of the network; and a_i_ is the fraction of weighted k neighbors attached to the nodes in community i, i.e. k_i_/2m^78,79^. (2) The *Clustering Ratio* (*C-ratio*) is the ratio of the number of network clusters to the count of the connected nodes in the network. (3) The average *Clustering Coefficient* (*C*) is defined as the ratio of the triangles impingent on a node to the connected triples, determined as a global average over all nodes in a simplified (undirected/unweighted) network^60,69,70^. *C* is not meaningful for strictly bipartite or scale-free graphs^73^. We also report coefficients of linear regression of *C* over the age and size of the networks of domain organization. (4) *The Fast-Greedy Community* (*FGC*) agglomerative hierarchical algorithm detects community structure for networks with m edges, n nodes, and a depth d of the dendrogram describing the community structure, given an optimized linear running time of O(m × d × logn) ~ O(n × log^2^n)^86^. The *Newman-Girvan* algorithm index (*NG*) was computed with two different input partitions, the first (5) defined by age (*NG*_*age*_) and the second (6) defined by VOS clustering (*NG*_*vos*_). *NG* calculates the modularity of a network given a predefined division or partition to measure the influence of the partition in separating the different node types. This indicates either assortative (positive) or disassortative (negative) mixing across modules^65^. The *NG* algorithm computes an index as 1/(2 m)∑_ij_(A_ij _− 1/(2m)k_i_k_j_ × ∆(c_i_,c_j_)), where m is the sum total of weights in the graph and A_ij_ are weighted entries in the adjacency matrix of the network; k_i_ | k_j_ and c_i_ | c_j_ are the weighted degrees and the components (numeric partitions) of the nodes i and j, respectively; finally, ∆(x,y) equals 1 if x = y and 0 otherwise^65^. The *VQ*, *C-ratio*, *C* and *FGC* indices each range from 0 to 1, while the *NG* indices range from − 1 to 1. In all cases, higher values represent strong modularity of the network at an event of evolutionary history. Heatmaps of modularity were constructed using log10-scaled modularity matrices, with each map element given as (A_ij _− k_i_k_j_/(2m))M_*nd*_, where A_ij_, k_i_, k_j_ and m were the same as defined for *NG*^65^, while M_*nd*_ was the network’s modularity index at event *nd*. Cladistic representations of modularity were visualized with dendrograms whose metrics were calculated from squared Euclidean distance matrices, which indicate dissimilarities between cluster means^87^. The dissimilarity or distance matrices were clustered hierarchically using the Ward's minimum variance method that seeks compact and spherical clusters^88^.

#### Quantifying randomness in networks

The Bartels rank test of randomness, which primarily offers a rank version of von Neumann's Ratio Test for Randomness^89^, was used to measure random network behavior. The resultant test statistic RVN is defined as ∑_i=1→n−1_ (R_i_−R_i+1_)^2^/∑_i=1→n_ (R_i _– (n + 1)/2)^2^, where R_i_ = rank (X_i_) with i = 1…n, (RVN − 2)/σ is the asymptotically standard normal, and σ^2^ = [4(n − 2)(5n^2^ − 2n − 9)]/[5n(n + 1)(n − 1)^2^]. The null hypothesis of this method was randomness, which was tested against the alternate hypothesis of non-randomness, given a trend of RVN values. A *p* value is computed from a two-sided beta distribution approximation test. Random graph controls were created by following the Erdős–Rényi graph model^61,90^.

## Supplementary Information

Supplementary Figure S1.

Supplementary ﻿Figure S2.

Supplementary ﻿Figure S3.

Supplementary ﻿Figure S4.

Supplementary ﻿Figure S5.

Supplementary ﻿Figure S6.

Supplementary ﻿Figure S7.

Supplementary ﻿Figure S8.

Supplementary ﻿Figure S9.

Supplementary ﻿Figure S10.

Supplementary File 1.

Supplementary File 2.

Supplementary File 3.

Supplementary Information.

Supplementary Video 1.

Supplementary Video 2.

Supplementary Video 3.
