# Supplementary figures and images for: Evolution of networks of protein domain organization
Source: Sci Rep. 2021 Jun 8;11:12075. doi: 10.1038/s41598-021-90498-8 (PMC8187734; doi:10.1038/s41598-021-90498-8)

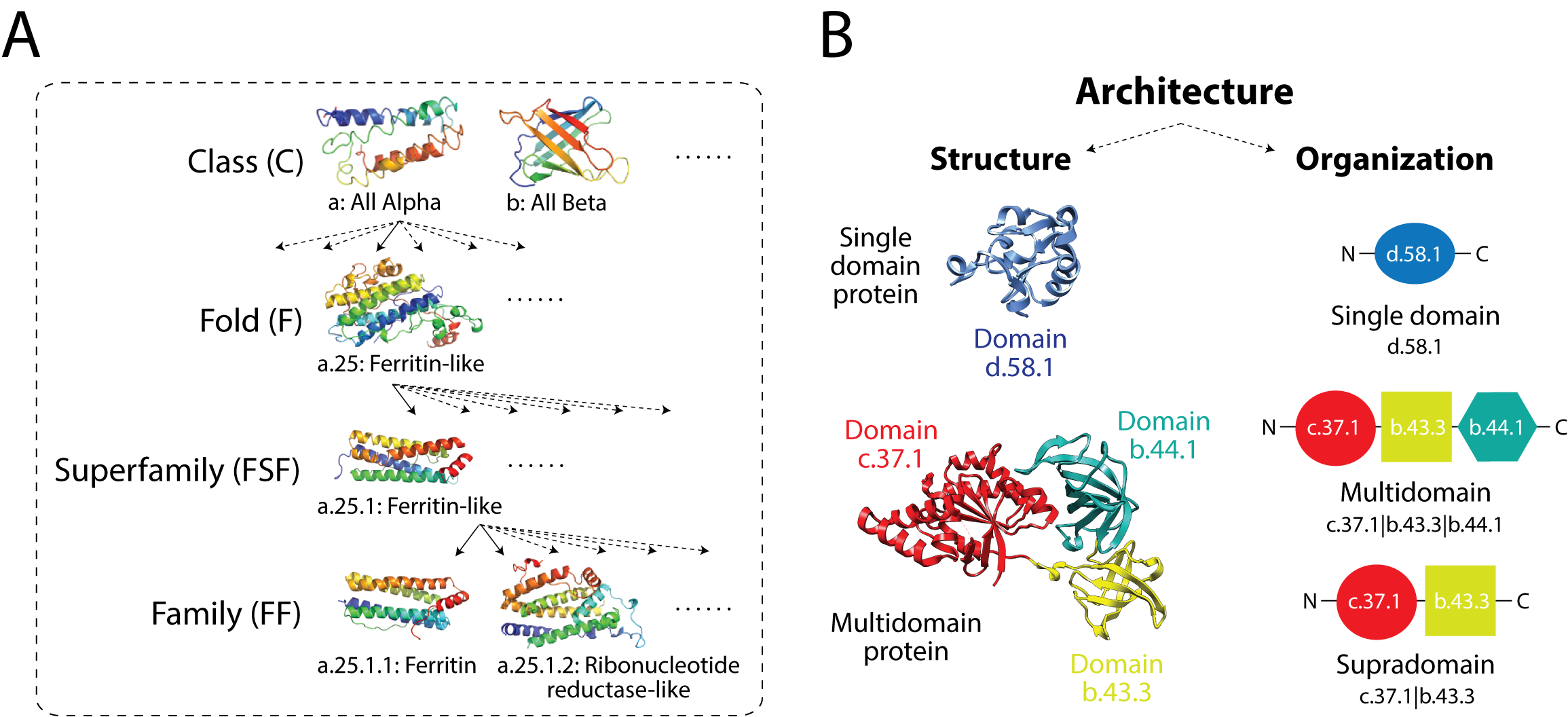

Supplement: Supplementary file 1 — Supplementary Figure S1. [file 41598_2021_90498_MOESM1_ESM.tif]

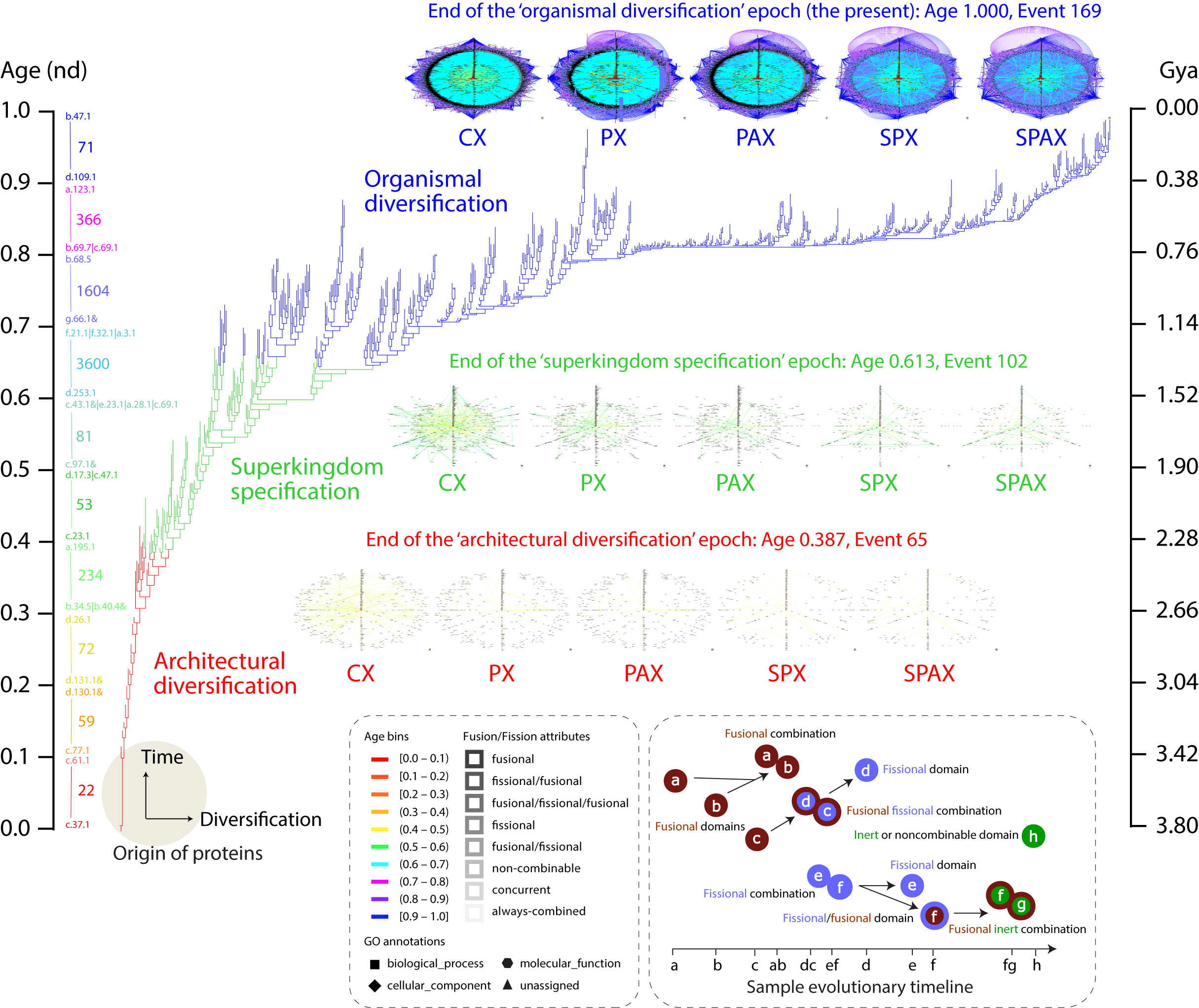

Supplement: Supplementary file 2 — Supplementary ﻿Figure S2. [file 41598_2021_90498_MOESM2_ESM.tif]

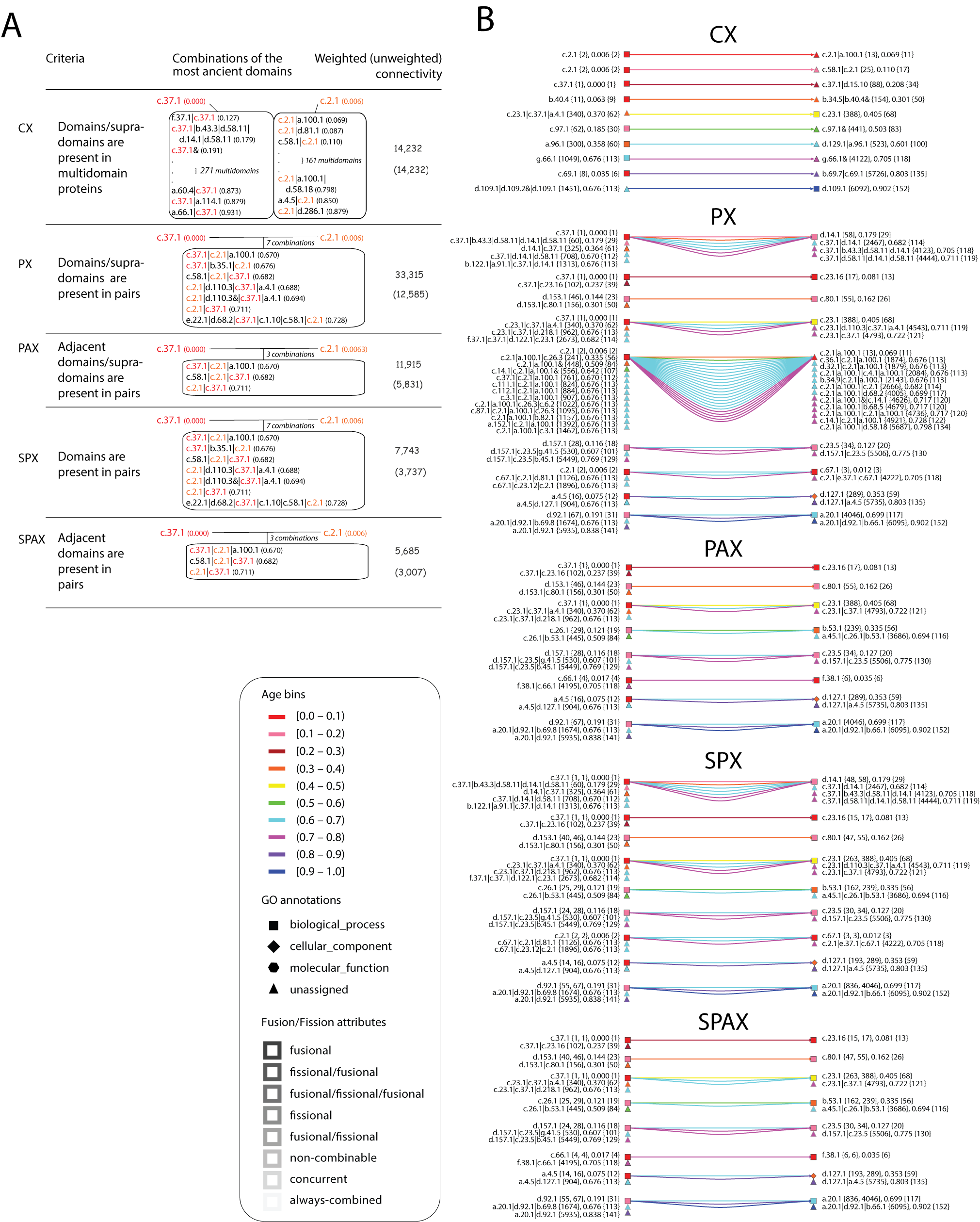

Supplement: Supplementary file 3 — Supplementary ﻿Figure S3. [file 41598_2021_90498_MOESM3_ESM.tif]

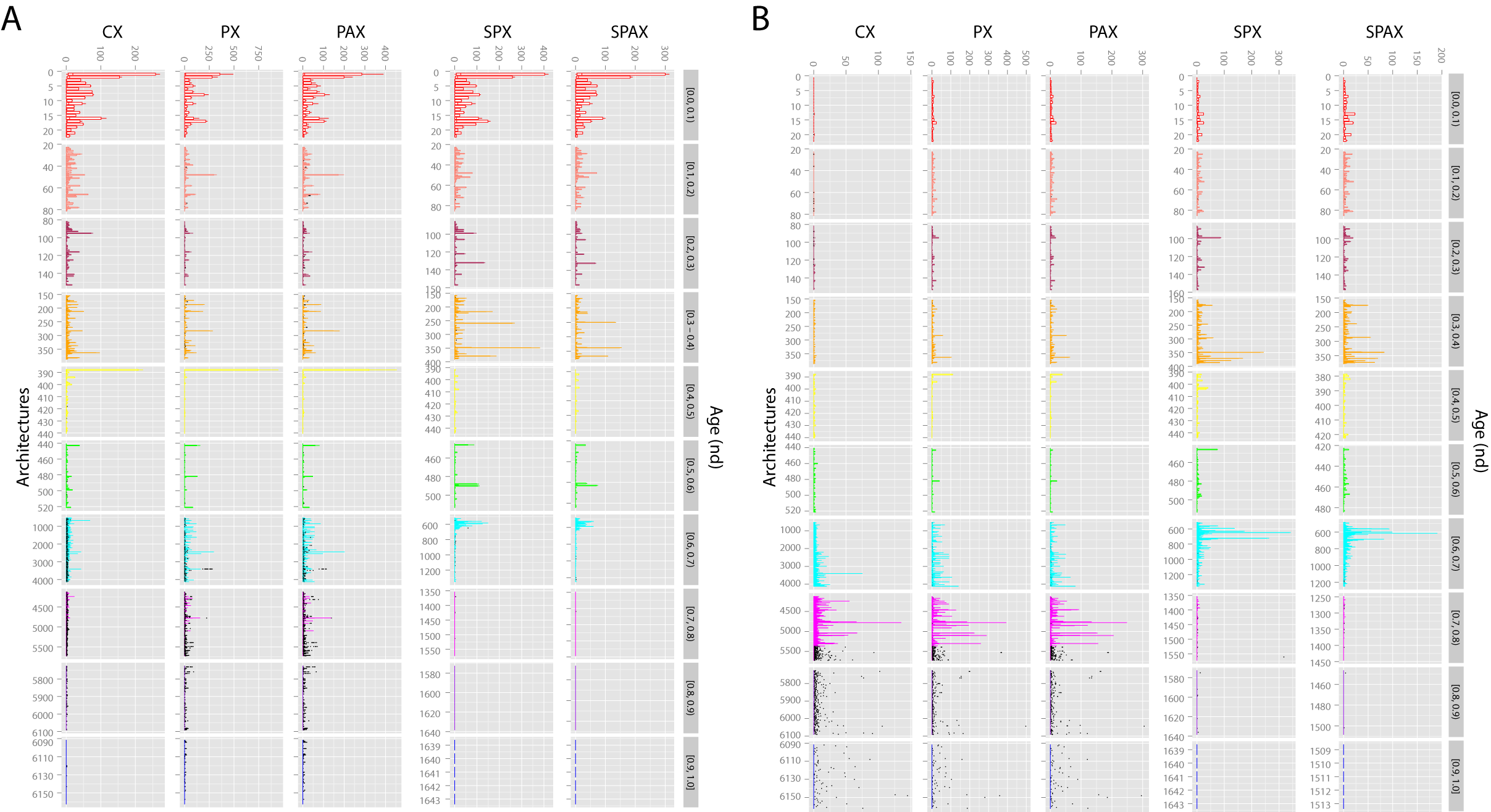

Supplement: Supplementary file 4 — Supplementary ﻿Figure S4. [file 41598_2021_90498_MOESM4_ESM.tif]

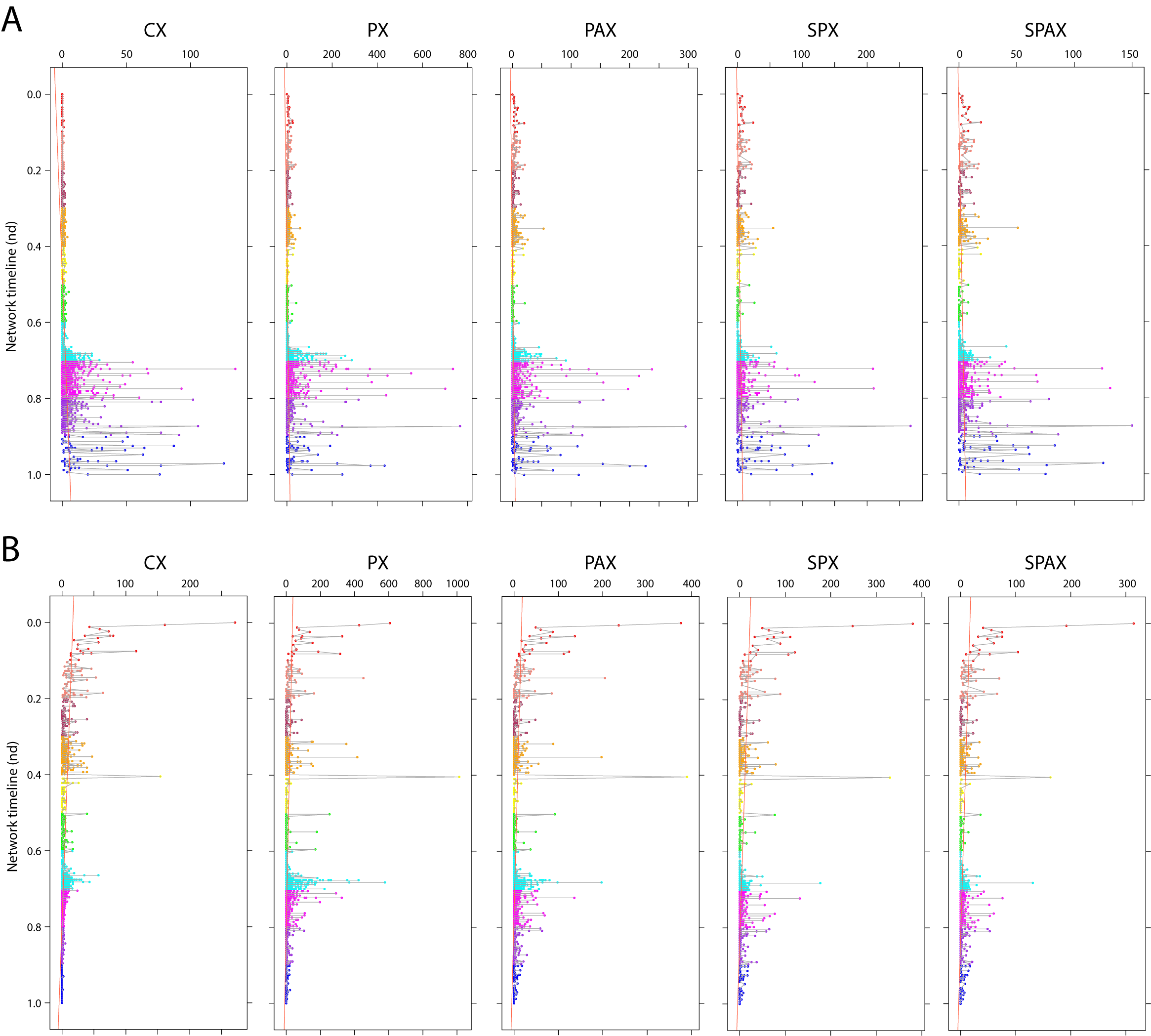

Supplement: Supplementary file 5 — Supplementary ﻿Figure S5. [file 41598_2021_90498_MOESM5_ESM.tif]

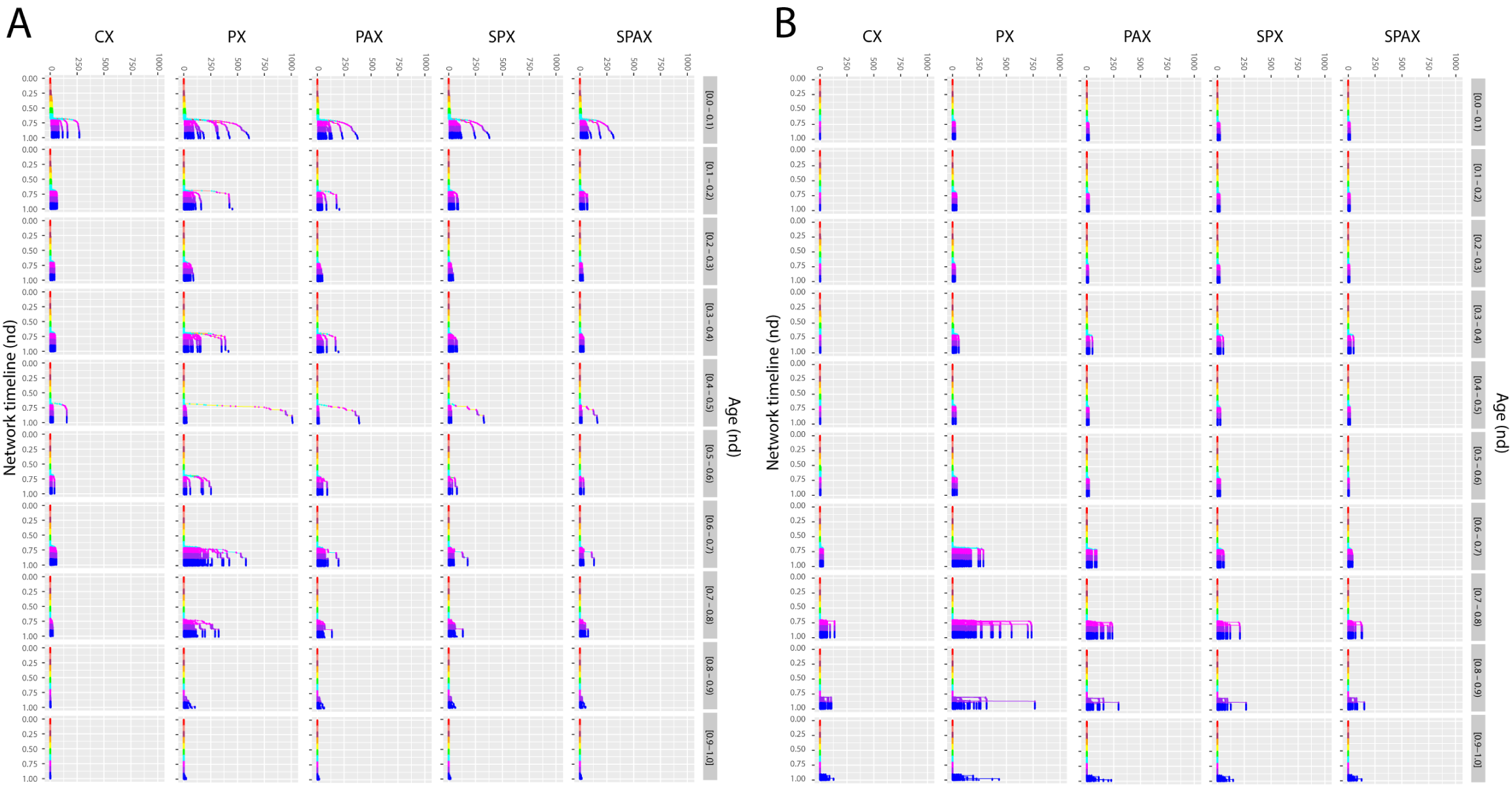

Supplement: Supplementary file 6 — Supplementary ﻿Figure S6. [file 41598_2021_90498_MOESM6_ESM.tif]

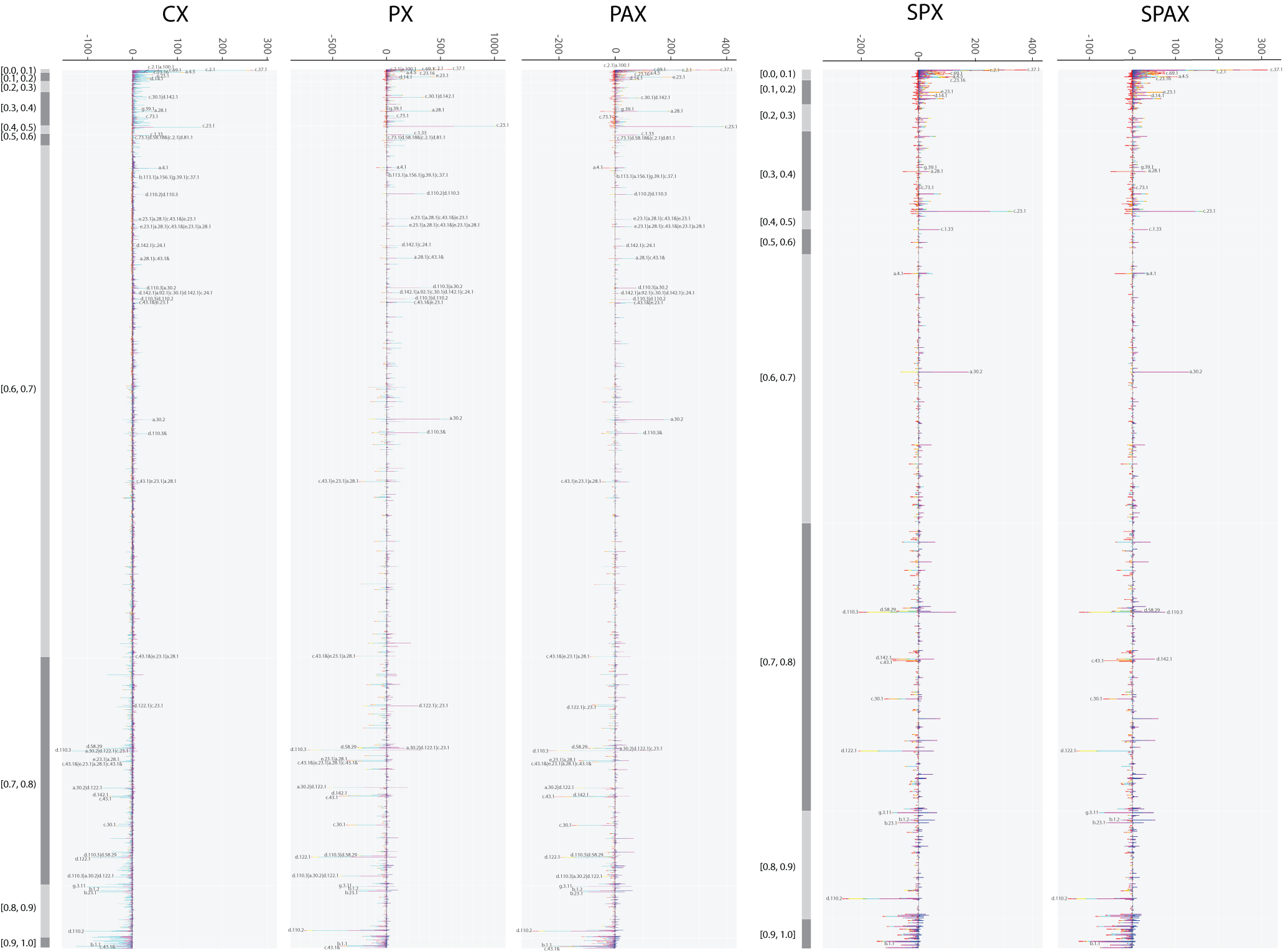

Supplement: Supplementary file 7 — Supplementary ﻿Figure S7. [file 41598_2021_90498_MOESM7_ESM.tif]

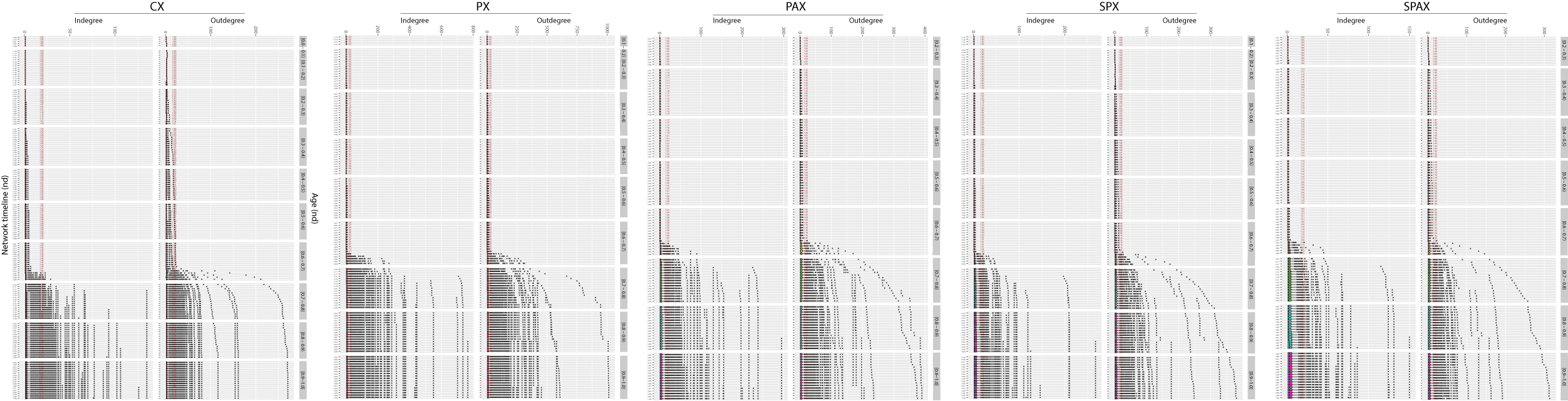

Supplement: Supplementary file 8 — Supplementary ﻿Figure S8. [file 41598_2021_90498_MOESM8_ESM.tif]

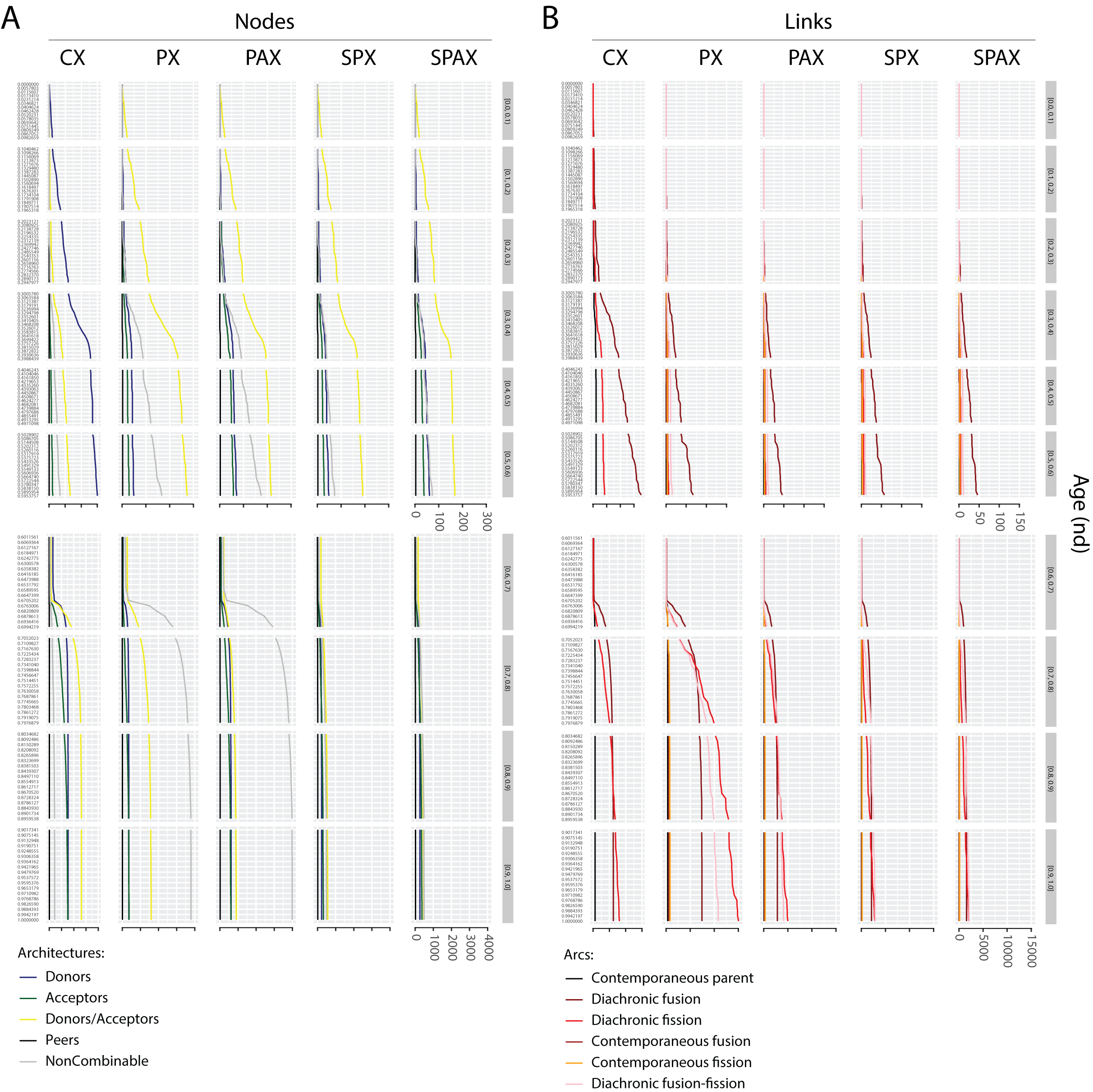

Supplement: Supplementary file 9 — Supplementary ﻿Figure S9. [file 41598_2021_90498_MOESM9_ESM.tif]

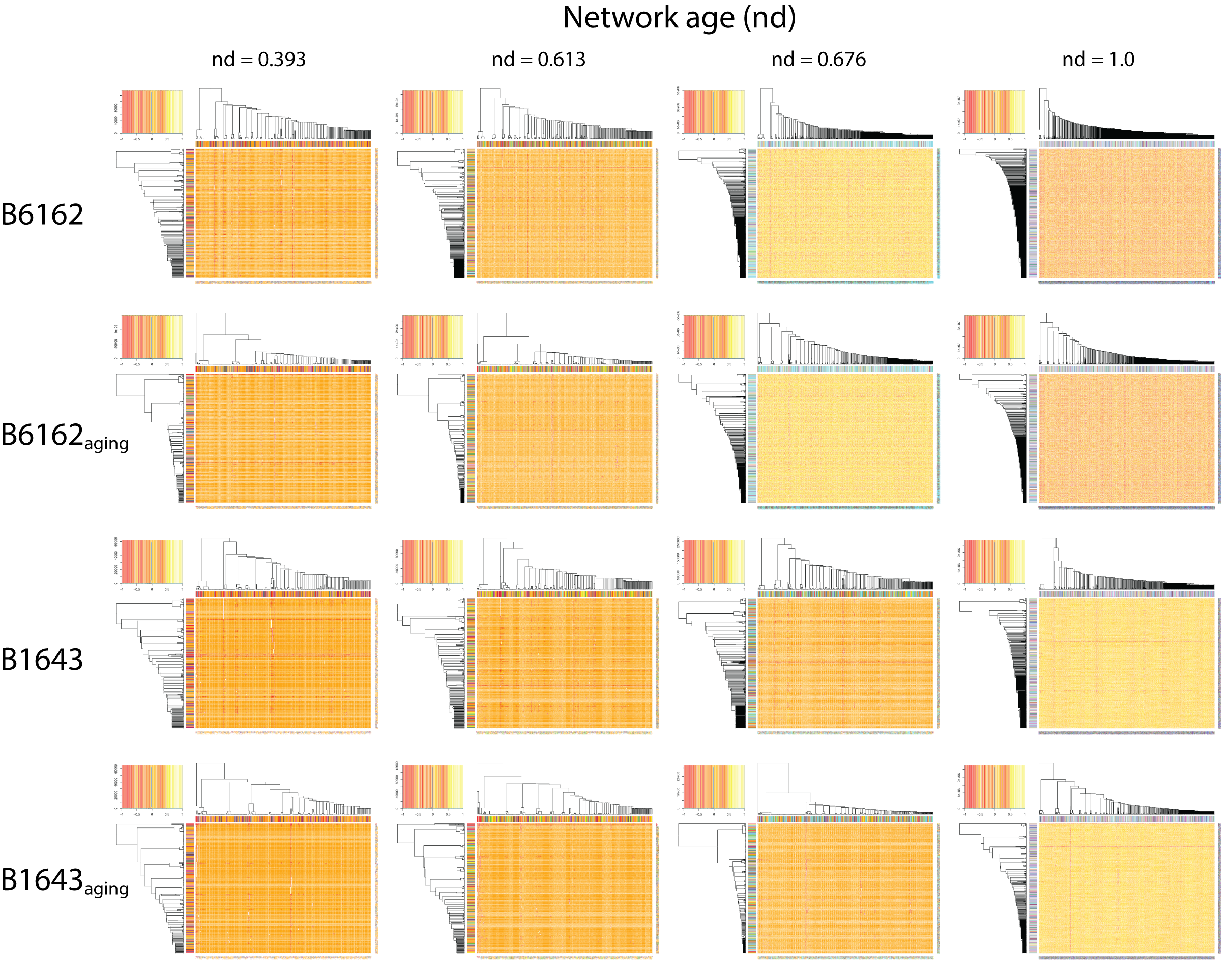

Supplement: Supplementary file 10 — Supplementary ﻿Figure S10. [file 41598_2021_90498_MOESM10_ESM.tif]
