# Supplementary material for: Evolution of networks of protein domain organization
Source: Sci Rep. 2021 Jun 8;11:12075. doi: 10.1038/s41598-021-90498-8 (PMC8187734; doi:10.1038/s41598-021-90498-8)
Supplement: Supplementary file 14 — Supplementary Information. [file 41598_2021_90498_MOESM14_ESM.docx]

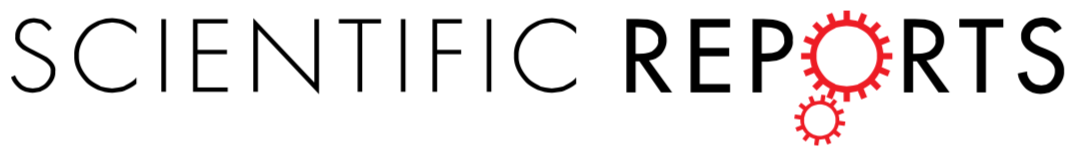


**Evolution of Networks of Protein Domain Organization**

**M. Fayez Aziz and Gustavo Caetano-Anollés**

Evolutionary Bioinformatics Laboratory, Department of Crop Sciences, University of Illinois, Urbana, IL 61801, United States. Correspondence and requests for materials should be addressed to G.C.-A (e-mail: gca@illinois.edu)

**Supplementary Information**

Supplementary Text pages 1-15

*Section 1 – Network Connectivity Criteria*

*Section 2– Early History of Modern Domain Organization*

*Section 3 – Network Analysis of Cooption Mechanisms of Recruitment*

*Section 4 – Hubs in Network Evolution*

*Section 5 – The Big Bang of Protein Domain Organization*

*Section 6 – Emergence of Preferential Attachment in Network Evolution*

*Section 7 – Emergence of hierarchical modularity during evolution of primordial structures*

*Section 8 – Supplementary Materials and Methods*

*References*

Supplementary Figures pages 16-25

Supplementary Tables pages 26-68

Supplementary Videos and Files page 69

**Supplementary Text**

***Section 1 ­­– Network Connectivity Criteria***

Here we describe in detail the connectivity criteria that were used to construct the networks of domain organization (Fig. 1B). Network links were defined by composition, pairwise occurrence, adjacency (topology), and splicing criteria: (i) *composition* describes makeup (component parts) of the molecular whole; (ii) *pairwise* *occurrence* describes appearance of parts in sets of two (pairs) in a molecule; (iii) *adjacency* refers to the geometrical, spatial or topological arrangement of parts; and (iv) *splicing* refers to the rearrangement of parts by operations of joining and excision that decompose structures.

A) *Composition Network (CX):* CX is a ‘composition' network because it captures the make-up of supradomains and multidomains directly from the domain organization of proteins. CX is also a partial bipartite graph-theoretic representation of domain organization, which can be decomposed into two network projections using mathematical properties of finite graphs^91^. The *‘domain organization’* (DO) bipartite graph consists of two disjoint sets of nodes, one describing structures that are present in supradomains and mutidomain proteins and the other describing their domain constituents. In theory, the graph can project connectivity within each set of nodes^92^, i.e. DO can be decomposed into two projections, a *‘domain’* (D) network and a *‘multidomain’* (M) network. However, the multimeric *‘supradomains’* (S) introduce an intricate third middle network layer. All networks can potentially display evolutionary information through evolutionary ages assigned to the nodes and connectivity components of the networks. Notice that the make-up and association of domains and multidomains that exist in proteins at molecular level define the nodes and links of the networks. Hence, the higher the connectivity of domains, supradomains and multidomains in the bi-modal DO graph, the larger the connectivity would be of the associated D and M uni-modal graphs. In CX, a domain node connects through a link to a multidomain node when the former is present in the domain combination, i.e. when the multidomain shares that domain. For example, consider domains 1, 2 and 3 connect to (are part of) a multidomain 1|2|3. Similarly, those same three domains will connect to another multidomain 1|3|2 because they are also part of that domain organization. The CX bipartite network is partial because the multidomain node set also harbors a significant number of links within the set. These additional links describe how smaller domain combinations present in the *ccs* census (that we term supradomains) can be component parts of larger architectural counterparts. For example, supradomains 1|2 and 2|3 connect to (are part of) the multidomain 1|2|3. Note that supradomains 2|1 or 1|3 are not part of 1|2|3 because they have discordant architectural topologies (which are considered an intrinsic property of the different architectural modules) and are not part of the census. To summarize, CX is a partial bipartite network that links various forms of domain organization like domains, supradomains and multidomains that are present in multidomain proteins. As a result, domains and multidomains that do not share domain organization remain isolated from the rest. The entity set of CX has 6,162 nodes connected by 14,232 links (Supplementary Table 1).

B) *Pairwise Network (PX):* PX is a ‘pairwise’ network because domain and supradomain nodes are connected to each other ‘in pairs’ when they are present in a parent multidomain, regardless of topology. These pairs help decompose domain organization into component parts, which become domain and supradomain network nodes. For example, multidomain 1|2 holds one domain pair (1 and 2) that must always induce a link between domain 1 and domain 2 in the network. Similarly, multidomain 1|2|3 holds 3 domain pairs (1 and 2, 1 and 3, and 2 and 3), which induce links between the domains of each pair. Note that PX does not enforce topological constraints among domain pairs of the molecule (e.g. domain order in the sequence). For instance, each of the following three multidomains, 1|2|3, 1|3|2, 2|1|3 or 3|1|2, will induce identical connectivity despite harboring distinct topologies. As with CX, pairs of supradomains present in multidomains, along with domains, will induce links between domain and/or supradomain nodes. For example, the presence of supradomain 1|2 together with multidomain 1|2|3 in the census will induce a link between the supradomain node 1|2 and the domain node 3. If 2|3 was also present in the census, then there would be a link between 1|2 and 2|3 supradomain nodes as well, despite the overlapped domain 2. PX can be considered a partial projection of CX that captures pairwise events instead of composition. The network criterion was inspired by the use of domain couplings in the study of structural and functional diversity of proteins^6^. The entity set of PX has 6,162 nodes connected by 33,315 links (Supplementary Table 2).

C) *Pairwise Adjacency Network (PAX):* PAX follows the operative criterion of PX but is a ‘pairwise adjacency’ network that applies a topological constraint of adjacency to the sequence of domains and supradomains in multidomains. We consider it biologically relevant to capture the sequence contiguity information in domain organization, especially because the sequences of domain interfaces often behave as evolutionarily conserved motifs that are responsible for optimal entropy dissipation and function^6,93^. Thus, adjacency provides evidence for coevolution in domain organization. Domain and supradomain nodes are connected ‘in pairs’, when two adjacent structures occurring contiguously in the sequence are present (without overlap) in a multidomain, regardless of node order. For example, multidomain 1|2|3 holds two pairs of adjacent domains, 1 and 2, and 2 and 3, which induce links between the respective domains. Similarly, multidomain 1|3|2 holds two adjacent domain pairs that are contiguous in the sequence, 1 and 3, and 3 and 2, which would link domains 1 and 2 each with domain 3. Now, given a multidomain 1|4|2|3, and supradomains 1|4 and 2|3 present in the census, there would be a link between 1|4 and 2|3 supradomain nodes as well, because these two supradomains are adjacent in the sequence without overlap. Moreover, a pair consisting of adjacent domain and supradomain would also induce a link in that contiguous pair. In the multidomain 1|4|2|3, for example, there would be links induced between respective adjacent domains and supradomains, 1 and 4|2, 4 and 2|3, 2 and 1|4, 3 and 4|2, 1 and 4|2|3, and 3 and 1|4|2, provided that the various structures are present in the census. Note that domains/supradomains that are not adjacent remain isolated in the network. The entity set of PAX has 6,162 nodes, connected by 11,915 links, a significantly reduced number as compared to PX due to adjacency constraints (Supplementary Table 3).

D) Spliced Pairwise Network (SPX)

SPX is a partial subset of PX and a domain projection-like of the CX network. Nodes represent domains that have ‘spliced’ from proteins, very much as exons are spliced from genomic sequence. SPX nodes arise from computation of a ‘domain set’ from the *ccs* census. The connectivity criterion is the pairwise presence of domains in a multidomain configuration regardless of topology constraints (which resembles that of PX). For example, multidomains 1|2|3, 1|3|2 and 1|2 generate a total of three links between domains 1 and 2 because the multidomains collectively harbor three pairs of those domains. The three domain combinations also generate two links between domains 2 and 3, and two links between domains 1 and 3. The weight of connections derived from link enumeration reflects the involvement of domains in the combinatorics of domain organization, and indirectly the evolutionary effects of fusion and fission processes in shaping the generation of architectural modules^7^. For that reason, non-combinable domains of single-domain proteins remain isolated in the network. When tracing the evolution of these networks, the age of spliced domains from various multidomain sources was opted based on the most ancient occurrence. SPX comprises of a computed set of 1,643 domains extracted from the original set of 6,162 domains and domain combinations, and 7,743 links of pairwise interaction (Supplementary Table 4). The extraction of these domain units from the original data set involved two categories: primarily single domains and unique spliced domains. The latter was further sub-categorized based on the three prominent types of domain combinations: domain repeats, multidomains with no repeats, and multidomains with repeats, in that order (Supplementary Table 6).

E) *Spliced Pairwise Adjacency Network (SPAX):* SPAX is a subset of SPX and PAX. The domain nodes are comprised of the same single domains and spliced domains node-set of SPX. Connectivity is however constrained by the 'adjacency in the sequence' criterion of PAX. Hence, nodes connect only when a pair of adjacent domains (i.e. occurring contiguously) in a sequence is present in a multidomain configuration, regardless of other topological order considerations. For example, multidomains 1|2|3 and 1|3|2, and supradomain 1|2 generate a total of two links between domains 1 and 2, because they collectively harbor only two pairs of these domains contiguously. The multidomains also generate two links between domains 2 and 3 (because the two domains in the pairs 2 and 3, and 3 and 2 are adjacent in the sequence), but only one link between domains 1 and 3. To summarize, in SPAX, single and spliced domains that pair adjacently in multidomain proteins are linked, while nonadjacent and non-combinable domains remain isolated. Therefore, SPAX explores the effect of proximity in amino acid sequence on fusion and fission processes responsible for domain combinatorics. SPAX comprises of the computed set of 1,643 domains extracted from the original set of 6,162 domains and domain combinations, and 5,685 links of pairwise interaction, a significantly reduced number as compared to SPX due to adjacency constraints (Supplementary Table 5).

Progression of the network connectivity across the various phases of the phylogenetic timeline was also explored to highlight the impact of connectivity criteria on network evolution (Supplementary Fig. S2), network snapshots along the evolutionary timeline (Fig. 2), and example connectivity of the most ancient domains and that of the earliest nodes from the 10 age bins (Supplementary Fig. S3).

***Section 2 – Early History of Modern Domain Organization***

To dissect evolutionary patterns of the early origin of proteins and illustrate the versatility of the waterfall visualization strategy, we focused on the SPX network. The SPX directed graph had 7,743 arcs, 1,643 nodes, a *network density* of 0.003 [7,743/(1,643×(1,643−1))], an *average outdegree|indegree* of 4.713 (±0.449/±0.372) and a *total degree* of 9.425±0.656. The SPX network also showed significant community structure with 607 clusters and a high modularity index of 0.945. The outdegree of domain nodes indicates the ability of domains to act as ‘donors’ (sources) of structures to domain combinations while their indegree depicts their ability to act as ‘acceptors’ (sinks). We distill the waterfall diagram by further dissecting the connectivity percentiles, based on outdegree, indegree and total degree. To simplify the complex network, we studied the total degree of the SPX waterfall at the 100^th^ percentile of connectivity (Fig. 3). The evolutionary transition from wide to tall node-symbols revealed the source-sink origination dynamics of molecular structure (Supplementary Video 1). This transition not only expressed the higher probability of recruitment of ancient domains by more recent domains, as expected, but also the anomalous existence of exchange-hubs in the middle of protein evolution. Remarkably, two major waves of structural innovation were observed in the waterfall diagrams of the highly connected SPX subnetwork (Supplementary Video 2). These observations conform to recent findings about the exploration of the evolution of bipartite networks of elementary functionomes^46^ and previous evolutionary studies of metabolism^54^. Although having separate evolutionary origins, these waves involved common sandwich, barrel and bundle protein domain structures.

*Wave 1:* The larger ‘first’ wave originated in the P-loop containing nucleoside triphosphate (NTP) hydrolase domain (c.37.1) and its uniquely connected but relatively more derived NAD(P)-binding Rossmann-fold (c.2.1) and alpha/beta-hydrolase (c.69.1) domains (Fig. 3). The eminent Rossmanoid α/β/α-layered c.37.1 domain structure is the most ancient in the timeline of domain history^8,56^. The *p*-loop prototype also enabled the nucleotide triphosphate binding functions of the P-loop hydrolase fold with its Walker A (*p*-loop) sequence motif. The wave segment that originates from the Rossmann c.2.1 structure, which typically embeds the β/α-barrel structures that are widespread in metabolism, connects to the glutathione synthetase ATP-binding domain-like (d.142.1) structure and then to the PreATP-grasp domain (c.30.1) terminal domain node. Wave segments originating in c.69.1 and c.2.1 connect via the (trans)glycosidases (c.1.8) and galactose-binding domain-like (b.18.1) structures to two more terminal derived structures. c.37.1 shares part of its wave with c.69.1 through nucleotide cyclase (d.58.29) and with c.2.1 through the PYP-like sensor domain (PAS domain) (d.110.3) and a major hub, the CheY-like (c.23.1) structure. The wave terminates at the most recent but active sinks, the concanavalin A-like lectins/glucanases (b.29.1) and protein kinase-like (PK-like) (d.144.1) domains. An interesting motif of the wave is the relatively recent triangular link pattern established between EGF/Laminin (g.3.11), b.18.1 and b.29.1. This wave of the most highly connected subnetwork of the SPX network demonstrates that the ‘*p*-loop’ (c.37.1) established a massive pathway of domain recruitment including the ancestral domain hub c.23.1, the downstream and highly connected domain d.110.3, and terminal domain d.144.1. The most ancient domains of the wave involved a strong recruitment pathway spanning ~0.5 billions of years (Gy) of history. This pathway enabled a host of functions related to metabolism and translation, with their 3-layered α/β/α structures and closed or partly-opened β-barrel structure. However, the later origination of the major source hub c.23.1 from c.37.1 is quite ancient in protein evolution and appeared as far back as 2.3 Gy ago (*nd* = [0.4, 0.5)).

*Wave 2:* Soon after the first *p*-loop wave was established, a ‘second wave’ originated ~3 Gy ago (*nd* = [0.2, 0.3)) in the ‘winged helix’ DNA binding domain (a.4.5) that holds important structural motifs for nucleic acid recognition (Fig. 3). This wave was constrained in scope with its major part merged with the *p*-loop wave through the major donor hub c.23.1, the highly connected sink/source d.110.3 and the terminal acceptors GAF domain-like (d.110.2) and d.144.1. Downstream to the major source hub c.23.1, two structures i.e. homodimeric domain of signal transducing histidine (a.30.2) and ATPase domain of HSP90 chaperone/DNA topoisomerase (d.122.1), merge into a triangle motif with the first wave through d.58.29. Historically, the a.4.5 origin of the wave harbors a parent structure, the DNA/RNA-binding 3-helical bundle fold (a.4) flanked by a 4-strand β-sheet. Moreover, the a.4.5 domain uniquely exposes elbows between helix-turn-helix motifs that specify atypical protein-protein and protein-RNA interactions in enzymes. These winged structures celebrate a central role in transcription, and in providing nucleic acid clamping capacity and flexibility to RNA polymerases^94^. The a.4.5 domain also provides interface surfaces for domain-domain recognition in protein complexes like polymerases, ubiquitin-ligases and condensins.

***Section 3 – Network Analysis of Cooption Mechanisms of Recruitment***

Our understanding of structural evolution is limited. There has been a scarcity of alternative hypotheses that could explain the evolution of macromolecules and non-static connectivity patterns responsible for emerging protein structure and function. A popular proposal is that new molecular forms arise by ligation of small polypeptides with somewhat irregular structure, morphing into stable larger peptides^55,74^, which then combine to form defined folded topologies in small protein domains^95–97^. A continuum of these prior structures is expected when invoking the principle of spatiotemporal continuity. We get a glimpse of such continuity from the interpolating connectivity scatter grams that were extracted from the final degree distributions at *nd* = 1 and elicit continuous tidal patterns of highs and lows of mutually-facilitated structural innovation of domains and domain combinations (Supplementary Fig. S5). We further explored the combinatorics of evolving networks by dissecting network connectivity with bar plots of chronological accumulation of links along the evolutionary timeline (Supplementary Fig. S7). This makes patterns quantitative and source-sink relationships explicit. For example, we noticed that most domain acceptors populated the recent organismal diversification epoch that started 1.5 Gy ago (*nd* ≥ 0.6). Note that 8 *nd* values were missing in the timeline of the domain-only SPX and SPAX networks: 0.046, 0.087, 0.168, 0.173, 0.416, 0.538, 0.618, 0.954. Additional quantitative insight was obtained from box-and-whisker plots of final weighted indegree and outdegree data of evolving networks (Supplementary Fig. S8). While architectural reorganization during the 0.4 ≤ *nd* < 0.9 period was overwhelming, the most recent sink protein structures act as acceptors of very ancient structures of *nd* < 0.1. Overall, acceptor sink structures drew innovation from donor source structures spanning the entire timeline, taking advantage of the repertoire of very ancient as well as ancient but more derived domain organization. Individual architectures thus significantly coopted numerous ancient counterparts to mold their functional tasks. This evolutionary pattern of recruitment is similar to that inferred from the enzymatic analysis of metabolic networks^98^.

We now reveal that the architectural formation of reusable domains and larger proteins with more complex domain organization, in the two most ancient and massive waves of innovation and recruitment that we identified (Fig. 3), continues to occur and does so at different rates (Supplementary Fig. S7). First, early domains provided the raw materials for recombination with newer domains along the entire 3.8 Gy history of proteins. Their ongoing history suggests the existence of an evolutionarily active repertoire of domains and architectural forms that are evolvable and not relics waiting for extinction. This conservation of sequence motifs as structural forms and their combinatorial reuse serves to recognize a hierarchy of structural complexity that is found closely interrelated throughout protein history. Second, connectivity patterns support a non-sequential and parallel buildup of domain and multidomain repertoires, suggesting different evolutionary rates of the two forms of domain organization. While domain and domain combination motifs that are reused in evolution provide strong support to ‘molecular canalization’, i.e. structural endurance despite changes in context and environment, the continued push toward architectural innovation and change, throughout evolution, is remarkable. The evolving domain structures embody a limited set of sequence motifs within individual folds, which are classified by SCOP families. This embodiment of motifs by structures can be used to study architectural combinatorial reorganization. From a historical point of view, structurally and energetically stable domains have higher chance to prevail and impart useful functions to multidomain proteins through properties that would ensure persistence of the emerging cells, including ribosomal activity, molecular transport, bioenergetics, and biosynthetic functions. Interestingly, phylogenomic analysis showed similar dynamics of combinatorial use of domains in multidomain proteins^7^. We find that domains that were relatively older than others contributed significantly to the combinatorial expansion of the protein world through fusion and fission processes (Supplementary Fig. S2). Based on similar observations made from expansion patterns of networks of domain organization with time, we hypothesize that the older domains essentially “donated” crucial structures and functions that were developed at the inception of the protein world. Conversely, younger domains “accepted” the prior structures in various molecular contexts to enhance functional diversity. The CX network showed that (1) relatively ancient donor domains may fuse to recruiting acceptor multidomains, and (2) donor multidomains undergo fission and modularize into relatively recent acceptor domains. Supradomain and domain pair-based PX, PAX, SPX and SPAX networks reveal that relatively ancient donor domains developed conserved evolutionary ties or partnerships with relatively recent acceptors in mutually shared multidomain (Supplementary Fig. S3). These pairs historically developed such that (3) fusional donors and acceptors paired in fusional multidomains, (4) fissional donors and acceptors diverge from fissional multidomains, and (5) fusional donors merge into fusional/fissional multidomains wherefrom fissional acceptors split off. Noticeably, a large number of domains in the evolving networks act both as donors and acceptors, suggesting robustness that has been acquired during the historical processes of evolution (Supplementary Tables 1-5). This age-based directional feature of the networks of domain organization provides an evolutionary signal to discern the ancient but ongoing processes of cooption.

***Section 4 – Hubs in Network Evolution***

Table 1 lists donor hubs from the five networks of domain organization. CheY-like (c.23.1), P-loop containing nucleoside triphosphate hydrolases (c.37.1), Homodimeric domain of signal transducing histidine (a.30.2), Acetyl-CoA synthetase-like (e.23.1), NAD (P)-binding Rossmann-fold domain (c.2.1) and ACP-like (a.28.1) were the most prominent donor hubs in the PX network with outdegree > 400. Domains c.37.1 and c.2.1 are the two most ancient domains and have been previously known for versatility and robustness^99^. The harbinger domain a.4.5 (*nd* = 0.075) of the second wave of structural innovation followed closely behind the 99.9^th^ percentile in the PX network with outdegree 188. Also, domain c.23.1 follows closely behind the 99.9^th^ percentile in the SPAX and CX networks with outdegrees 163 and 154, respectively, a.30.2 in the PAX, SPX and SPAX networks with outdegrees 197, 177 and 131, respectively, e.23.1 in the PAX network with outdegree 205, c.2.1 in SPX, PAX, SPAX and CX networks with outdegrees 248, 236, 192 and 161, respectively, and a.28.1 in PAX network with outdegree 197. Domain c.2.1 is involved in co-factor biosynthesis, which is a significant primordial function that stabilized membranes and supported the early evolution of molecular structure and function^55^. Remarkably, domain hub c.23.1 appears after the superkingdom specification epoch (0.391 < *nd* < 0.614)^42^. This late occurrence of a robust hub at the cusp of a new era highlights that the versatility of domain recruitment is not confined to the ancestral domains of the architectural diversification epoch (0 < *nd* < 0.391). Other significant donor domains in the PX network were alpha/beta-hydrolases (c.69.1), PYP-like sensor domain (PAS domain) (d.110.3) and Class I glutamine amidotransferase-like (c.23.16) with outdegree > 300, and EAL domain-like (c.1.33) with outdegree > 250.

Table 2 lists acceptor hubs for the networks of domain organization. The GAF domain (d.110.2), the PYP-like sensor domain (PAS domain) (d.110.3) and the ATPase domain of HSP90 chaperone/DNA topoisomerase (d.122.1) were the most significant acceptor hubs in the PX network with indegree > 700. The GAF domain (with GO annotation of ‘purine-containing compound catabolic process’) is a signaling small-molecule binding domain involving GMP receptors that shares a similar fold with the PAS domain. The PAS domain (with GO annotation of biological process ‘regulation of cellular macromolecule biosynthetic process’) is common in signal transduction proteins (e.g. two-component systems) and is implicated in both small molecule binding and protein-protein interaction^100^. The GAF and PAS domains appeared during the late organismal diversification epoch (0.614 < *nd* <= 1.0)^42^. CoA-dependent acyltransferase domain (c.43.1) was above the 99.9^th^ percentile in the PX network with indegree > 400. Interestingly, domain d.110.3 was also a significant donor hub in the modern protein world. Four domains from PX network qualified as acceptor hubs from the organismal diversification era with indegree > 300: PreATP-grasp domain (c.30.1), Immunoglobulin (b.1.1), Glutathione synthetase ATP-binding domain-like (d.142.1), EGF/Laminin (g.3.11). Their ages range from *nd* =0.723-0.977.

***Section 5 – The Big Bang of Protein Domain Organization***

A massive wave of domains and domain combinations was made evident during the initial phase of the organismal diversification epoch (0.614 < *nd*_FSF_ ≤ 1.0)^42^ by the large number of terminal branches of short length that appeared in the phylogenomic trees^7^. This burst of protein innovation was coined “big bang” after an analogy to the cosmological origin of the universe. Evolving networks reveal network snapshots at time events of choice (Fig. 2). To study the big bang of domain organization, we first identified an evolutionary period spanning a short series of time events (0.659 < *nd*_FSF_ <= 0.826) with a significant abundance spike of architectural novelties. Architectural diversity increased (relative to *nd* = 0.671) from 776 to 2,202 domains and domain combinations in the CX, PX and PAX networks, and from 389 to 514 domains in the SPX and SPAX networks. Time events *nd* = 0.671 and *nd* = 0.676 spanned the largest abundance increase, empirically defining the start of the big bang and offering a rich repertoire of emerging network links. For convenience, we refer to these time-events as ‘before’ and ‘after’ the big bang. We noticed changes in network connectivity and explored salient characteristics of domain organization during the big bang transition.

An analysis of donor/acceptor hubs (at a 99.9^th^ percentile of connectivity) that are associated with the big bang of domain combinations showed that the most ancient P-loop containing nucleoside triphosphate hydrolase (c.37.1) structure was the only donor with outdegree above the 99.9^th^ percentile in all 5 network types (Table 1). Domain c.37.1 continued to be the most significant donor in all five networks after the big bang. This supports its central role in jumpstarting the combinatorial processes of the big bang. The second most ancient and versatile NAD(P)-binding Rossmann-fold domains (c.2.1) structure^6^ was a significant donor in the PX, CX and SPX networks after the big bang. Remarkably, the central role of the c.2.1 primordial hub of co-factor biosynthesis and membrane stabilization^55^ continues after the big bang. A third ancestral domain from the architectural diversification epoch (0 < nd < 0.391)^42^, the acetyl-CoA synthetase-like (e.23.1) structure, also gained significantly high donor connectivity after the big bang. Not only e.23.1 showed active combinatorics in the PX network, it was also one of the leading domains after the big bang following behind the 99.9^th^ percentile in PAX, CX, SPX and SPAX networks, with outdegrees 29, 21, 18 and 15, respectively. Among the donor domains comparatively below the 99.9^th^ percentile, c.23.1 followed closely behind the c.2.1 and e.23.1 domains in the PX, CX and PAX networks with outdegrees 54, 45 and 21, a.28.1 in the PX, PAX and CX networks with outdegrees 59, 31 and 18, c.69.1 in the PX, CX, PAX and SPAX networks with outdegrees 56, 29, 23 and 23, c.23.16 in the PX, CX, PAX and SPX networks with outdegrees 38, 16, 16 and 13, and c.1.33 in the CX and PX networks with outdegrees 12 and 10. These domains span ages in the range *nd* = 0.035 - 0.503, which shows a diverse and disassortative pattern of co-options during the combinatorial interplay that follows the big bang. Remarkably, the cofactor/carrier domains e.23.1, a.28.1 and c.69.1 continue their role as donors of pre-ribosomal NRPS pathways during the post big bang protein world to coordinate de novo protein synthesis^55^. Our analysis thus uncovers this significant association between composition, pairwise and adjacency criteria of evolving networks of domain organization and NRPS’s machinery of assembly line protein synthesis in secondary metabolism.

The acceptor domains that were prominent before the big bang were only evident in the PX network, with indegree equal to or more than the 99.9^th^ percentile, namely Homeodomain-like (a.4.1), Carbamate kinase-like (c.73.1), Ribosomal protein S5 domain 2-like (d.14.1), Glucocorticoid receptor-like (DNA-binding domain) (g.39.1) and CheY-like (c.23.1) (Table 2). These domains appeared during the late architectural diversification epoch (0 < *nd* < 0.391) and during the early superkingdom specification epoch (0.391 < *nd* < 0.614)^42^. Notably, c.23.1 that has a significant role in co-factor biosynthesis was also a popular donor of the networks of domain organization following the big bang and until modern time. Domains a.4.1 and d.14.1 were also significant acceptors in CX and SPX networks, respectively. However, the only domain that was significant after the big bang with indegree more than the 99.9^th^ percentile was Acyl carrier protein (ACP)-like (a.28.1). Remarkably, these hub domains share a historical role of DNA binding and editing that is ancient in evolution of molecular function. a.4.1 homeobox domain proteins are transcription factors that bind DNA through a helix-turn-helix (HTH) structure. c.73.1 dimer domains consist of a 16-stranded beta-sheet that is surrounded by alpha-helices, and convert carbamoyl phosphate and ADP into ammonia, carbon dioxide and ATP as a last step of the arginine dihydrolase (AD) pathway. d.14.1 domains are found in RNA/DNA-binding proteins and kinases sometimes with a left-handed, 2-layer alpha/beta fold whose core structure consists of beta (3)-alpha-beta-alpha. g.39.1 are also DNA-binding domains involving glucocorticoid receptors. c.23.1 single domain proteins are often present at N-terminal of a DNA-binding effector domain and consist of a 3-layer alpha/beta/alpha sandwich with a histidine protein kinase phosphorylation region that activates responses to environmental signals as part of a bacterial two-component signaling system. Finally, a.28.1 domains that carry the pantetheine-4'-phosphate prosthetic group, provide a 'swinging arm' for the attachment of activated fatty acids and amino-acid groups that are essential cofactors of the modular NRPS systems. Examples include lipoylation of pyruvate, growth and development of Gram-positive organisms and the synthesis of a variety of microbial bioactive peptides. Notably, these domains partake extensively in pairwise and adjacency networks, highlighting their ancient role in primordial functions of protein translation of genetic code. Interestingly, none of the domains that gained significance as acceptor hubs in the modern protein world were among the significant acceptors before or after the big bang. This shows that the process of recruitment is ongoing and that there is high probability that novel domains were not molecularly canalized but rather were evolvable forms that supported innovation and modularization. Remarkably, as we continue to explore the transitions in network states at the big bang, a significantly large number of multidomain proteins originate or gain acceptor activity after the big bang. It should also be noted that the role of acceptor hub shifts to novel domains during organismal diversification, i.e. in the era following the big bang (Supplementary Fig. S9).

***Section 6 – Emergence of Preferential Attachment in Network Evolution***

In a previous effort to identify power law patterns in evolution of protein domain organization, selected protein sequences were studied to identify the interactions among their evolutionary units, i.e. the individual domains. The emergent network of pairwise associations of SCOP domains showed that only a few domains were ubiquitous in most of the multidomain proteins and that the domain combinations thus followed a scale-free pattern under the principle of preferential attachment^6,101^. However, many other biological networks could not be fully explained by a fitted power law distribution. Metabolic networks, in particular, deviate from scale-free behavior that assumes a perspective of highly optimized tolerance to external attacks^102^. In contrast to scale-free networks, such “self-dissimilar” or scale-rich networks do not fragment on removal of a few high-degree hub nodes^103^. In a relevant study, interactions among metabolites of *Escherichia coli* were induced by various stresses^50^. The resulting metabolomes were rewired in a fashion that did not follow power law behavior. Here, we investigate the degree distributions of the evolving networks of domain organization for indications of scale-free patterns.

We tested the chronological accumulation of time-directed connectivity by using the outdegree and indegree data and derived statistics relevant to various power law tests (Fig. 4). An emergent property of power law and associated generative models was observed only in the evolving CX network and earlier phases of the other networks. We deduced this by analyzing the scaling response of the node degree as the networks grew along the time-events of the evolutionary timeline. Most statistics failed to reject preferential attachment in CX and the connectivity distributions of the ancient nodes in PX, PAX, SPX and SPAX. Thus, evolution of composability of domain organization (CX) and the pairwise cooption of early architectures followed scale-free features that organized networks appearing before the big bang. We focused on indegree data (i.e. the connectivity of acceptors) to verify our power law statistics, due to the more substantiated usage of indegree for scale-free behavioral studies^85^. However, we noticed a close approximation of data curves representing the outdegree (i.e. the connectivity of donors) as well. The primary indicator we used to scrutinize scale-free behavior was the Kolmogorov-Smirnov (KS) statistical test of power law fit^84,85^. Lower KS fit statistic (≤0.1) and higher p-values of the KS test (>0.05) supported power-law fitting of network degree distribution. We also compared the scale-free behavior of evolving networks with a randomness criterion called von Neumann's Ratio Test for Randomness (RVN)^89^. The resultant patterns resembled a yin-yang of rise in the power-law index accompanied by a reduction in randomness and vice versa (Supplementary Fig. S10A). Another indicator, the exponent (α) of the fitted power law distribution is usually α > 1 for power law fitted *P* (X = x^–α^) distributions. In our networks, α was much higher during early protein evolution and then stabilized to a value of ~2 during modern times (Fig. 4). These patterns suggest a gradual deviation from power-law decay as the networks evolved. To ensure data quality, the log-likelihood and the coefficient of determination (R^2^) parameters of the fitted power law distributions were also monitored. The log-likelihoods dropped significantly lower than zero for the networks during the modern era of the timeline, making power law distributions less likely late in protein evolution. R^2^ in all networks was found conveniently higher than ~75% indicating strong confidence level of our degree data profiles and the linear models we explored.

***Section 7 – Emergence of hierarchical modularity during evolution of primordial structures***

We further tested the community structure of evolving networks with three additional statistical metrics of network modularity. For two of these metrics, the Newman-Girvan (*NG*) index was used that calculates edge ‘betweenness’ iteratively (Fig. 5). *NG* maximizes the number of shortest paths that run through a certain edge, while systematically removing edges of high inter-community centrality^65^. This iterative edge removal method yields an *NG* index that represents the network community structure, given an input partition vector (grouping or membership) to the *NG* algorithm. The *NG* index typically ranges from –1 to 1, with positive values indicating higher modular connectivity within groups and negative values indicating connectivity across groups. Firstly, we tested against the set of 169 ages (*NG_age_*), which primarily described ‘historical modularity’ of the evolving networks. Secondly, we partitioned the *NG* index by the Visualization of Similarity (VOS) method (*NG_vos_*), which describes the cohesiveness of clusters of low dimensionality^78,79^. Thirdly, we applied the Fast-Greedy Community (*FGC*) detection algorithm to get a hierarchical overview of the agglomerative clustering structure^86^. Remarkably, the patterns produced by the *NG_vos_* and *FGC* indices remained similar in terms of a growing community structure and agglomerative cohesiveness in the five evolving networks. In contrast, the evolving behavior of *NG_age_* was atypical. A trail of history-driven modular network structure was evident early in the timeline with *NG_age_* ~0 levels, indicating formation of small modules that were independent of age and free of scale (Figs. 4 and 5). This initial phase of formation of an age-diverse modular structure continued during the early architectural diversification, reducing the *NG_age_* to a lowest value of –0.5 (*nd* = ~0.1 in CX and ~0.2 in the other networks). These modularity patterns matched those we observed early on in the domain projection of the elementary functionome network, which describes the rise of protein structures and functions^46^. The value of *NG_age_* then gradually increased reaching a temporary plateau of ~0 at the start of the superkingdom specification epoch (*nd* = ~0.4). This intermediary phase indicated a trend towards formation of modules between nodes with similar ages, matching the modular behavior of the elementary functionome bipartite network later in evolution^46^. However, we observed an atypical second event of reduction in the value of *NG_age_* ~–0.1 during the big bang of domain combinations that was only evident in the CX and PX networks (Fig. 5). It illustrates that during early protein evolution, their domain structures were loosely coupled by age. Later on, this trend was overtaken by agglomerative network structure and the existing architectures engaged more freely in a widespread recruitment of emerging structures and functions. The modern times, however, harbored balanced community interactions in terms of age, indicating maintenance of specialized domain modules.

Notably, while CX is a scale-free (Fig. 4) and partial bipartite network with underrepresented triangles of node connectivity, it was included in our investigation of modularity due to extant *C*-based modular structure, along with its projection-like pairwise derivative networks of domain organization (Fig. 5). The modular behavior of the CX network, however, wavers throughout the evolutionary timeline. The early absence of modules (*C* = 0; 0 < *nd* < 0.25) was counteracted by a tendency towards maximum scale-free organization. *C* then spikes to 0.26 at *nd* = 0.26 and continues at ~0.2 and 0.30-0.35 before and after the big bang, respectively. During this period, preferential attachment had its second peak (*nd* = ~0.67). This indicates a more concentrated formation of hierarchical modules at both of these time events of CX evolution. Similarly, the pairwise domain and supradomain sharing PX and spliced domain sharing SPX networks showed an early lack of modularity (*C* = 0 until *nd* = 0.28) during a period when the scale-free property was on the rise (Figs. 4 and 5). This lapse in modularity was interrupted by a sudden spike to *C* of ~0.7 at *nd* ~0.3, followed by a plummeting slope, which reduced *C* to ~0.2 by *nd* ~0.4. This short time span of ~0.5 Gy hosts the first generative phase of hierarchical modularity of the PX and SPX networks. A second generative phase gradually raised *C* from ~0.5 in PX and from ~0.25 in SPX before the big bang to ~0.6 and ~0.3, respectively, after the big bang. Again, this phase was accompanied by a gradual decline of preferential attachment and a gradual rise of modularity, which continued until present times with *C* plateauing at second peaks of ~0.7 and ~0.4 in PX and SPX, respectively. Interestingly, modularity of the adjacency-based PAX and SPAX networks remained constrained until the organismal diversification epoch (*nd* = 0.66), during a period when the scale-free property was on the rise. Triangular modules resulting from adjacent sharing of domain organization only began appearing at the big bang, with *C* of ~0.1. This modular behavior then quickly flourished in an inverse exponential (logarithmic) fashion to a maximum *C* of 0.2 (*nd* = 0.72) before plateauing around that level late in evolution. The remarkable consequence of these findings is that topology appears to be a relevant factor for network structure only after the big bang of domain combinatorics.

We highlight these general recruitment trends with heat maps of *NG_age_* that describe the historical modularity of the evolving networks (Fig. 6). Cells colored in red demonstrate a semi-sigmoidal signal during early protein evolution (first three panels), diluting into a ubiquitous pinkish white pixelated pattern. Similar recruitment patterns were demonstrated by the *NG_age_*-based dendrograms accompanying the heatmaps, which showcase historical patterns of modular clustering in the evolving networks that were distinct from the long tail clustering patterns of preferential attachment (Supplementary Fig. S10B). These historical hues and cliques were stimulated by a self-organizing hierarchy guiding re-clustering of modules in macromodules, a feature exhibited by the scale-free hierarchical association of modules in metabolic networks^60^. The process ends with a free and independent generation of modules manifesting late in protein evolution. The mechanics behind the transformation from power-law behavior to hierarchical modularity (scale-free clustering) that later unfolded into modules of domain organization involves a hidden switch occurring ~3.4 Gy ago (Supplementary Video 3).

***Section 8– Supplementary Materials and Methods***

**Mathematical Definitions of Networks**

CX:

- Given:
  - Set A of domains and domain-combinations,
  - Set B of domain-combinations only,
  - where B ⊂ A (B is a proper subset of A),
- The composition-based network CX (V, E) is such that:
  - V (the set of vertices) = A ∪ B (= A), and
  - E (the set of edges) = {{a, b}: where a ∈ A, b ∈ B}, while
    - a is a substring of b, or
    - b contains a, or
    - a composes b, or
    - b has a

PX:

- Given:
  - Set A of domains and domain-combinations,
  - Set B of domains and domain-combinations,
  - Set C of domain-combinations only,
  - where C ⊂ A (C is a proper subset of A) and B = A,
- The pairwise network PX (V, E) is such that:
  - V (the set of vertices) = A ∪ B ∪ C (= A), and
  - E (the set of edges) = {{a, b}: where a ∈ A, b ∈ B}, while
    - ∃ c ∈ C | a is a substring of c ∧ b is a substring of c, or
    - c contains both a and b, or
    - a composes c ∧ b composes c, or
    - c has both a and b

PAX:

- Given:
  - Set A of domains and domain-combinations,
  - Set B of domains and domain-combinations,
  - Set C of domain-combinations only,
  - where C ⊂ A (C is a proper subset of A) and B = A,
- The pairwise adjacency network PAX (V, E) is such that:
  - V (the set of vertices) = A ∪ B ∪ C (= A), and
  - E (the set of edges) = {{a, b}: where a ∈ A, b ∈ B}, while
    - “|” denotes two elements joined adjacently
    - ∃ c ∈ C | “a|b” is a substring of c ˅ “b|a” is a substring of c, or
    - c contains either “a|b” or “b|a”, or
    - “a|b” composes c ˅ “b|a” composes c, or
    - c has either “a|b” or “b|a”

SPX:

- Given:
  - Set A of single and spliced domains only,
  - Set B of single and spliced domains only,
  - Set C of domain-combinations only,
  - where A ∩ C = ε (A and C are mutually exclusive) and B = A,
- The spliced pairwise network SPX (V, E) is such that:
  - V (the set of vertices) = A ∪ B (= A), and
  - E (the set of edges) = {{a, b}: where a ∈ A, b ∈ B}, while
    - ∃ c ∈ C | a is a substring of c ∧ b is a substring of c, or
    - c contains both a and b, or
    - a composes c ∧ b composes c, or
    - c has both a and b

SPAX:

- Given:
  - Set A of single and spliced domains only,
  - Set B of single and spliced domains only,
  - Set C of domain-combinations only,
  - where A ∩ C = ε (A and C are mutually exclusive) and B = A,
- The spliced pairwise adjacency network SPAX (V, E) is such that:
  - V (the set of vertices) = A ∪ B (= A), and
  - E (the set of edges) = {{a, b}: where a ∈ A, b ∈ B}, while
    - “|” denotes two elements joined adjacently
    - ∃ c ∈ C | “a|b” is a substring of c ˅ “b|a” is a substring of c, or
    - c contains either “a|b” or “b|a”, or
    - “a|b” composes c ˅ “b|a” composes c, or
    - c has either “a|b” or “b|a”

**Network data analyses.** The networks were visualized and analyzed using Pajek^76^ at multiple stages while they unfolded along the evolutionary timeline. The nodes (vertices) and links (arcs) data of networks were compiled in Excel spreadsheets. The quantitative network property of cumulative weighted degree per node (*cwdn*) was generated using custom Pajek macros. *cwdn* was formatted as three types of tabular data matrices for each network (BOX 1). The data rows of matrices defined domain organization and were sorted by age of either node (individual *nd*) or network (event *nd*), in ascending order. Separate data matrices were stored for the ‘in’- and ‘out’-degree types.

BOX 1. Types of data matrices

| Matrix type | Matrix objective |
| --- | --- |
| By ‘node age’ (NOA) | Used for box plots and x-y line plots. Categorical columns were ordinal number, age bin, age and node label of loop and domain nodes in the networks. Additional columns described *cwdn* in increasing order of events. Rows were sorted by node. |
| By ‘network age’ (NEA) | Used for power law distribution graphs. They are essentially transpositions of NOA data. Columns were ordinal number, age bin, and age of networks, followed by *cwdn* arranged by nodes. Rows were sorted by events. |
| By ‘degree dispersion’ (DD) | Used for stacked bar charts. Column and row order are the same as NOA data. However, columns provided the distributions of final *cwdn* (i.e. at *nd*=1) across connected node age bins. |

**Network visualization.** A set of Pajek menu commands was used to generate visualization attributes and layouts (BOX 2). Node categories were made distinguishable using various shape and color options. Evolutionary patterns were unfolded using age-based color-coding of domain organization nodes. A color palette was used that ranged from red for the most ancient node (*nd* = 0) to blue for the most recent node (*nd* = 1).

BOX 2. Network visualization tools and commands

| Pajek tool and command | Output |
| --- | --- |
| Network: Create Vector: Centrality: Weighted Degree: ‘All’, ‘Output\Input’ | The weighted degree vectors for undirected and directed networks, respectively. |
| Draw: ‘Network + Vector’ | Visualization of the weighted degree of nodes as node size. |
| Network: Create Partition: Communities: VOS clustering: ‘Multi-level coarsening + Multi-level refinement’ | The community-based layout of the networks, using the VOS clustering method^52,53^. In addition, modularity indices were obtained. Default parameters were used. |
| Draw: ‘Network + Partition’ and Draw: Layers: ‘In y Direction’ | Vertical arrangement of nodes according to their age in both bipartite and waterfall layouts of the networks with age mappings. |
| Draw: Layout: Energy: Kamada-Kawai: ‘separate components’ | Visual distribution of partitions^54^ (clusters or communities) in waterfall layout with the most energetically favored network configurations. |

**Charts and graphs.** Graphing code constructs and packages of R^78,79^ were used to visualize the *cwdn* (BOX 3).

BOX 3. Graphing and charting constructs and operations

| R *package*: function | Result |
| --- | --- |
| *Reshape: ** | Transform textual data tables to vector form. |
| *Lattice: ** | Generate panels of network- and node-age bins. |
| *LatticeExtra, grid: ** | Resize panels. |
| *LatticeExtra:* ‘panel.lmline ()’ | Draw linear regression model lines and equations, e.g. in *log-log* graphs. |
| *Data.table: ** | Edit data tables, e.g., to remove empty age bins. |
| *igraph: ** | Read network graph files to calculate modularity and apply the power law distribution model to calculate its fit statistics. |
| *R base*: ‘bwplot ()’ | Box and whisker’s plots based on NOA files, to depict measures of central tendency of *cwdn* over network age. |
| *Lattice:* ‘xyplot ()’ | XY scatter and line plots based on NOA files drawing final *cwdn* attained at network age 1. |
| *R color palette: ** | The color-coding of data points by node age. |
| *ggplot2:* ‘ggplot ()’, ‘geom_bar ()’ and ‘grid.draw ()’ | Produce degree dispersion stacked bar charts and graphical trend curves of power law behavior and modularity, based on DD, NEA data and VOS modularity report files, respectively. |
| *ggplot2:* scale_fill_manual () | Color-coded stacked bars representing distribution of final *cwdn* over ages of connected node set. |
| *ggplot2:* ‘layer ()’ | Stack negative valued bars in reflection. |
| *ggplot2:* ‘theme ()’ | Color-coding of multiple curves of power law statistics and modularity indices. |
| *gridExtra:* ‘grid.arrange ()’ and ‘arrangeGrob ()’ | Plotting together of curves for comparison. |
| *ggplot2:* ‘geom_smooth ()’ | Addition of linear regression lines to some modularity plots. |
| *ggplot2: ‘*geom_tile ()’, ‘scale_fill_gradient ()’, ‘scale_x/y_discrete ()’ etc. | Customized modularity heat maps. |
| *ggdendro: ** | Plotting of dendrograms. |
| *R base*: ‘aggregate ()’ | Calculate averages of categorized data. |
| *plotrix*: ‘std.error ()’ | Measure dispersion of data through standard error. |

**Barabási Control networks.** Reference networks for comparative analysis of power law and modularity were generated using ‘barabasi’^80^ methods of the R’s *igraph* package^77^. The ideal power law model was implemented with ‘barabasi.game ()’. An extended model was implemented with ‘aging.prefatt.game ()’. This latter model simulated the scale-free evolution of random graphs by altering the probability of preference of an old vertex growing multifold, exponentially with age. Networks of sizes 6,162 and 1,643 were created to simulate reference controls for the corresponding network groups of CX, PX, PAX and SPX, SPAX. Ages were assigned to individual nodes in incremental order to keep age proportion per event consistent with the timelines of the test networks.

**Power law statistics.** *P(k)* vs. *k* and *log-log* plots provided preliminary insight into the scale free behavior of a network. Power law behavior usually manifests in degree distributions exhibiting long tails (similar to *Poisson*). R libraries and operations were utilized to run the analysis (BOX 4). The distributions were color-coded by age.

BOX 4. R operations used in power law analysis

| R *package*: function | Result |
| --- | --- |
| *Lattice:* ‘xyplot ()’ | Plot distributions generated from NEA files. |
| *Data.table:* ‘table ()’ | Compute *k* frequency tables. |
| *R base*: ‘length ()’ | Calculate length of degree tables. |
| *Lattice:* ‘lm ()’ and ‘panel.abline ()’ | Generate and draw linear regression models. |
| *R base*: ‘summary (lm)$ coefficients’ | Retrieve the γ-slope power law coefficient and R^2^ determination coefficient. |
| *R base*: ‘ks.test ()’ | Calculate primary power-law stats from Kolmogorov-Smirnov Tests |
| *igraph:* ‘power.law.fit ()’ | Obtain additional statistics supporting the preferential attachment principle. |

**Modularity indices.** Modularity indices were calculated for each network using various capabilities of the *igraph* package^77^ (BOX 5). Isolated vertices were deleted using a custom function and corresponding partitions were adjusted, as required by the modularity algorithms. *cwdn* was used as input in calculation of all modularity indices. VOS modularity indices and partitions were generated using Pajek^52,53^. *NG* modularity indices^65^ were computed using two types of memberships (or partitions) as input: VOS clustering (*NG_vos_*) and age (*NG_age_*). Clustering ratios were determined using custom functions by dividing the number of clusters from the connected node set with its size.

BOX 5. R’s *igraph* package operations used in modularity analysis

| *igraph’s* function | Result |
| --- | --- |
| ‘read.graph ()’ | Import network graph data. |
| ‘fastgreedy.community ()’ | Calculate the *FGC* modularity index^75^. |
| ‘as.undirected ()’ | Collapse directed edges for *FGC.* |
| ‘modularity ()’ | Compute *NG* modularity indices. |
| ‘transitivity ()’ | Determine average clustering coefficient (*C*), using the ‘average’ option^65,70,71^. |

**Randomness quantification.** The randomness quality of domain networks was computed using the rank version of von Neumann's Ratio Test for Randomness a.k.a Bartels rank test of randomness. The network degree data from NEA files was input along with the parameters alternative="two.sided", to test null hypothesis of randomness against nonrandomness, and pvalue=beta, to compute p-value using an approximation given by the Beta distribution^89^. The algorithm removes missing values by default. The random graphs to be included as controls were generated per Erdős–Rényi model implementation of *igraph* package^77^ (BOX 6). The distributions were color-coded for better visual inspection.

BOX 6. R operations used in calculation of randomness

| R *package*: function | Result |
| --- | --- |
| *randtests:* ‘bartels.rank.test ()’ | Calculate von Neumann's Ratio Test for Randomness (RVN) over network degree^89^. |
| *igraph:* ‘erdos.renyi.game ()’ | Generate random graphs per the Erdős–Rényi model^61,62^. |

**Heatmaps and dendrograms.** *NG* pairwise modularity matrix was computed for each network using ‘modularity_matrix ()’^86^. Node-age memberships (or partitions) were used as input. Each matrix was scaled by log10 of the overall absolute *NGage* modularity score of the network, before generating a heatmap with ‘heatmap.2 ()’. Pairwise scores were adjusted to -1 to make the range [-1, 1]. The x-y axis node labels were color-coded and ordered by age. Dendrograms were generated using ‘dist ()’ by calculating squared Euclidean distance matrices that indicate dissimilarities between the cluster means^87^. The distance (or dissimilarity) matrices were hierarchically clustered using ‘hclust ()’ with the Ward's minimum variance method aiming at finding compact, spherical clusters^88^. Nodes of the dendrograms were color-coded and ordered within clusters by age.

**External support**: Additional information, including scripts and workflows for visualization and statistical analyses can be found in the Molecular Ancestry Networks (MANET) database repository (<http://manet.illinois.edu>).

***References***

91. Chung, F. R. K., Erdős, P. & Spencer, J. On the decomposition of graphs into complete bipartite subgraphs. in *Studies in pure mathematics, To the memory of Paul Turán* (eds. Erdős, P., Alpar, L., Halasz, G. & Saekőzy, A.) 95–101 (Budapest: Bikhäuser Verlag, 1983).

92. Diestel, R. *Graph theory. Graduate Texts in Mathematics*. vol. 173 (Heidelberg: Springer-Verlag, 2010).

93. Winter, C., Henschel, A., Kim, W. K. & Schroeder, M. SCOPPI: a structural classification of protein – protein interfaces. *Nucleic Acids Res.* **34**, D310--D314 (2006).

94. Teichmann, M., Dumay-Odelot, H. & Fribourg, S. Structural and functional aspects of winged-helix domains at the core of transcription initiation complexes. *Transcription* **3**, 2–7 (2012).

95. Berezovsky, I. N. & Trifonov, E. N. Van der Waals locks: loop-n-lock structure of globular proteins. *J. Mol. Biol.* **307**, 1419–1426 (2001).

96. Aharonovsky, E. & Trifonov, E. N. Protein sequence modules. *J. Biomol. Struct. Dyn.* **23**, 237–242 (2005).

97. Goncearenco, A. & Berezovsky, I. N. Prototypes of elementary functional loops unravel evolutionary connections between protein functions. *Bioinformatics* **26**, i497--i503 (2010).

98. Kim, H. S., Mittenthal, J. E. & Caetano-Anollés, G. Widespread recruitment of ancient domain structures in modern enzymes during metabolic evolution. *J. Integr. Bioinform.* **10**, 214 (2013).

99. Teichmann, S. A., Chothia, C. & Gerstein, M. Advances in structural genomics. *Curr. Opin. Struct. Biol.* **9**, 390–399 (1999).

100. Taylor, B. L. & Zhulin, I. B. PAS domains: internal sensors of oxygen, redox potential, and light. *Microbiol. Mol. Biol. Rev.* **63**, 479–506 (1999).

101. Apic, G., Gough, J. & Teichmann, S. A. An insight into domain combinations. *Bioinformatics* **17**, S83 (2001).

102. Tanaka, R. Scale-rich metabolic networks. *Phys. Rev. Lett.* **94**, 168101 (2005).

103. Li, L., Alderson, D., Tanaka, R., Doyle, J. C. & Willinger, W. Towards a theory of scale-free graphs: Definition, properties, and implications (Extended Version). *Arxiv Prepr. cond-mat/0501169* (2005).

**Supplementary Figures**


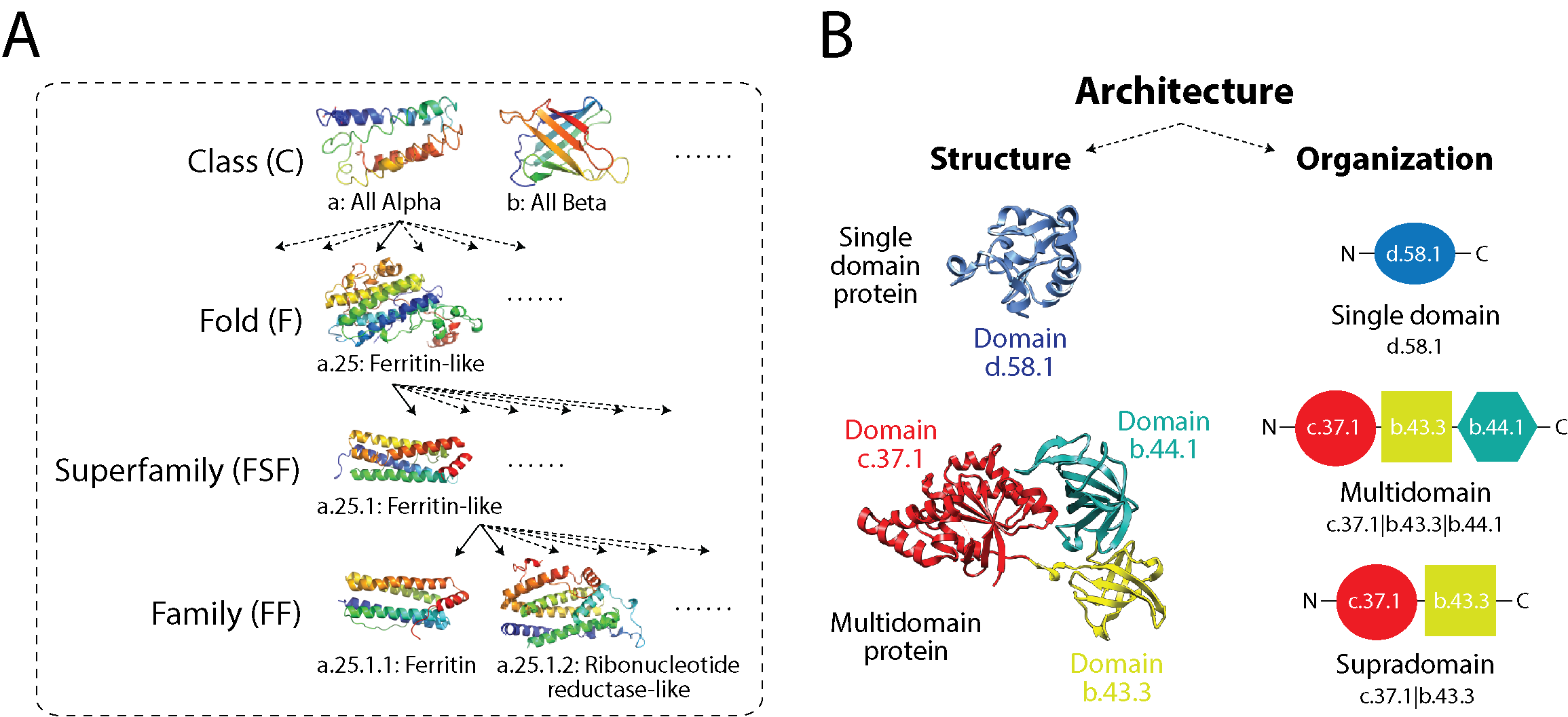


**Supplementary** **Figure S1. The structure and organization of proteins. A.** A protein is made of structural, functional and evolutionary units, the structural domains. Protein domains can be classified using the SCOP hierarchy of class (C), fold (F), fold superfamily (FSF) and fold family (FF). Currently, sets of 7 classes, 1,232 Fs, 2,026 FSFs, and 4,919 FFs of SCOPe 2.07 describe the world of proteins. **B.** Proteins have ‘architectures’ defined by their ‘structure’ (the folding of constituent domains in 3-dimensional space) and their ‘organization’ (the combinatorial ordering of domains along the polypeptide chain). We illustrate this structural and organizational complexity with single domain and multidomain protein examples, the ferredoxin of *Azotobacter vinelandii* (PDB entry 1a6l) and the translation factor eEF-1 alpha from *Sulfolobus sofataricus* (PDB entry 1jny). The bacterial ferredoxin contains only a single domain, with a 4Fe-4S ferredoxin (d.58.1) FSF structure. In contrast, the archaeal elongation factor is much more complex. It contains three domains, an N-terminal G-domain with a P loop containing nucleoside triphosphate hydrolase (c.37.1) FSF, a central domain with a translation protein (b.43.3) FSF, and a C-terminal domain with a EF-Tu/eEF-1 alpha/elF2-gamma C-terminal domain (b.44.1) FSF. The N-terminal and central domains form a ‘supradomain’ that functions as a module that hydrolyzes GTP to drive conformation change in the central translation domain. It is present as a more complex evolutionary unit in other initiation and elongation factor proteins (e.g. elF2 gamma, EF-G). Here we use the term supradomain as a sub-combination of domains that is repeatedly present in the architectural census and its modular role can be explored with networks of domain organization.

**
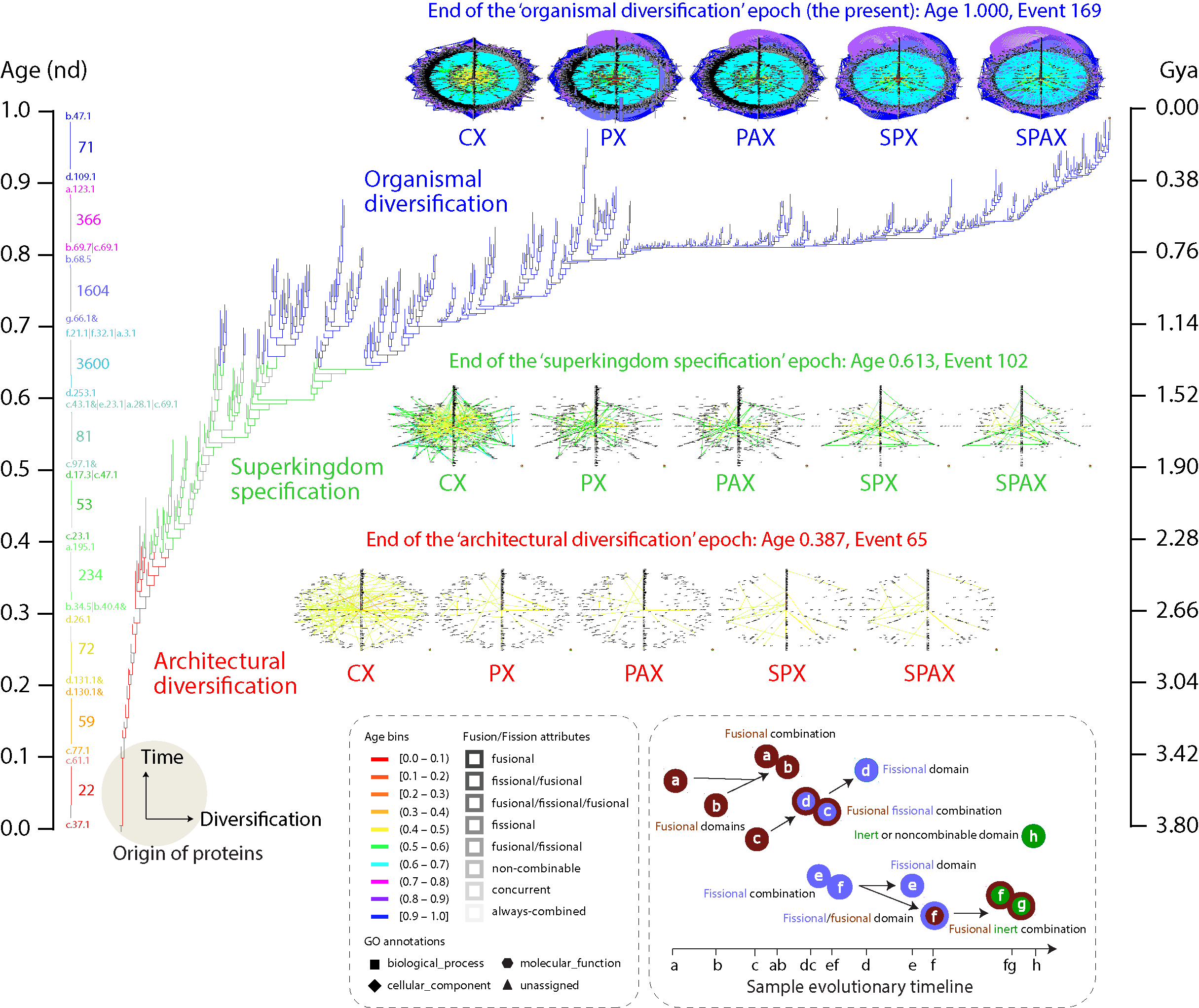
**

**Supplementary** **Figure S2. The phylogenomic tree of protein architectures embodied an evolutionary timeline of protein domain organization**. The optimal rooted phylogenomic tree reconstructed from genomic abundances of 6,162 protein architectures (length=623,423; CI=0.024; HI=0.976; RI=0.653; RC=0.016; G-fit=–2.895) is highly unbalanced. This property of tree topology allows to assign a relative evolutionary age to individual terminal leaves. This takes the form of node distance (nd) values normalized on a scale from 0.0 (the ‘root’ leaf) to 1.0 (the ’end’ leaf), which is depicted as an RGB gradient color scale with 10 bins on the left. SCOP *ccs* labels along the timeline, which correspond to taxa in the tree, describe the first and last architectures of each bin. Labels are color coded to depict progression of time with in-between numbers describing the count of architectures in each bin. The RGB gradient color scale with 10 bins on the right was derived from a molecular clock of folds, which calibrated the relative nd timeline according to a geological time scale that began 3.8 billion years ago (Gya). The colored branches of the tree represent the three evolutionary epochs of the protein world: architectural diversification (nd < 391), superkingdom specification (391 < nd < 0.614) and organismal diversification (0.614 < nd < 1.00)^7^. Snapshots of evolving networks of protein domain organization taken at the end of each of the three evolutionary epochs are described in radial format (Fig. 1C), with nodes representing architectures and color-coded arcs representing donor-acceptor recruitments and flow of time from ancient to recent nodes (arcs between contemporary nodes were excluded). Networks showcase gradual introduction of novel architectures by event 65 (red), the rise of architectural modularity by event 102 (green), and the massive preferential accumulation of architectures that followed the ‘big bang’ of domain combinations by event 169 (blue). Link and node RGB colors represent age while node grey-scale borders denote fusional/fissional properties. The vertical/horizontal node scales represent connectivity and the node shapes describe GO categories. See Supplementary Fig. S3 for further details. *Inset:* The order of appearance of architectures along the timeline defines fusion and fission processes of a combinatorial landscape that shapes the evolution of domain organization in proteins. Six categories of nodes are defined by these processes. Fusional domains (i) combine to form fusional combinations (ii) and fusional/fissional combinations (iii). The latter may eventually yield fissional domains (iv). A fissional combination (v) can split into fissional domains and fissional/fusional domains (vi). Two additional categories describe architectures that do not partake in the combinatorial processes. The three high-level attributes are color-coded as: fusion (dark red), fission (blue), and inert (green).


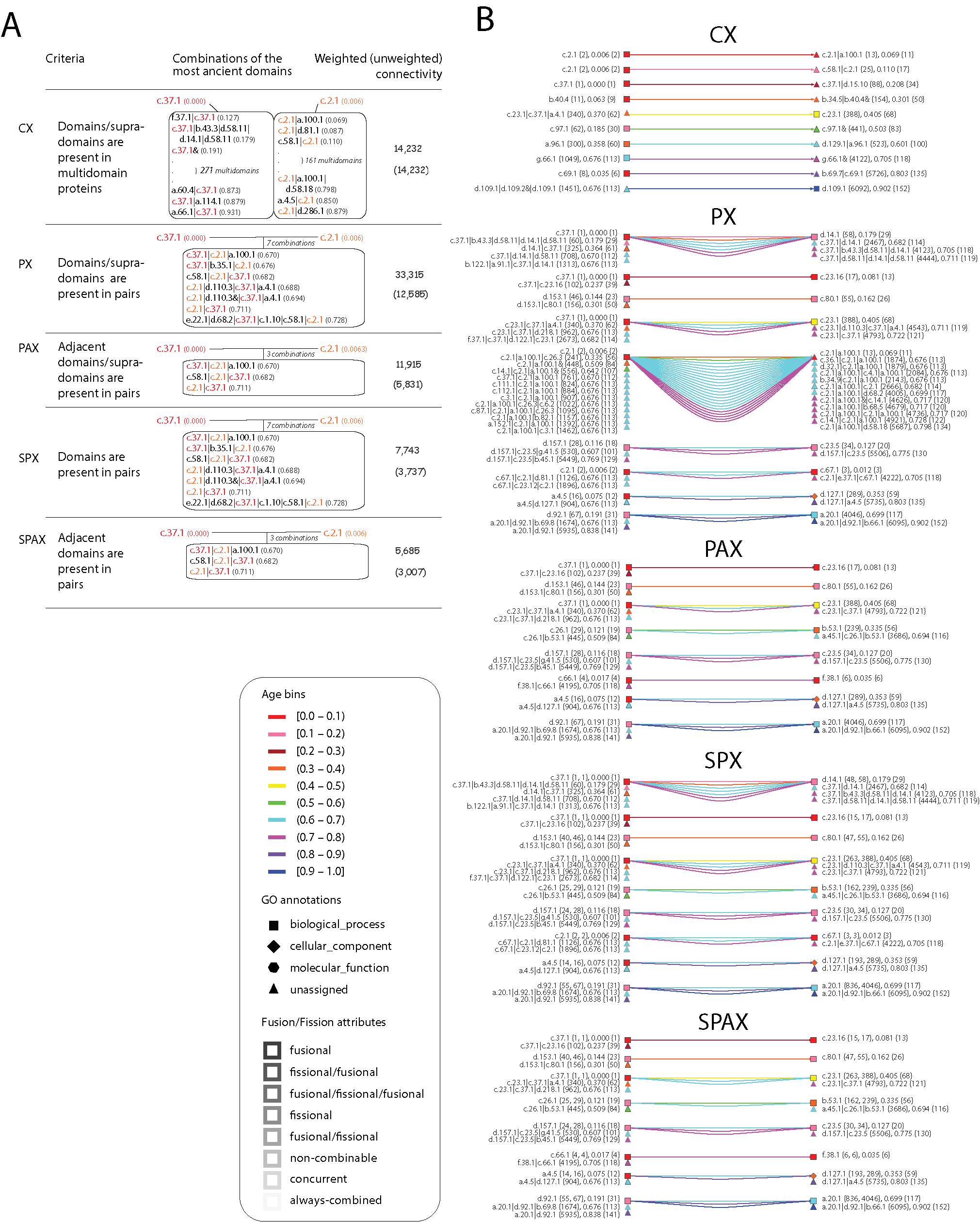


**Supplementary** **Figure S3. The evolutionary combinations of the most ancient structural domains. A.** We applied the five network generation criteria, which specify how domains, supradomains and multidomains interact with each other in a protein, to the two most ancient domain FSFs: The P-loop containing nucleoside triphosphate hydrolases (c.37.1) and the NAD(P)-binding Rossmann-fold domains (c.2.1), color-coded red and orange, respectively. Domains and their combinations are named using *ccs* labels and their ages (*nd* values) are provided in parentheses. Weighted connectivity accounts for multiple occurrences of the same domain interaction across various events in the timeline. Unweighted connectivity (in parentheses) regards those occurrences only once. **B.** Network links established throughout the timeline between the very first connected nodes in each of the 10 age bins are shown for the five networks of protein domain organization. The arcs are directed links from ancient to recent nodes. Their multiplicities (arched arcs) denote recurrent connectivity at various network events. Arc colors represent the age of the youngest among the two linked children nodes and parent architectures, which are listed under the links of the four pairwise networks. Consequently, the oldest age bin (red) is absent in PX and SPX, and the two oldest bins (red, salmon) are absent in PAX and SPAX. The angles of multiple arched arcs emerging from nodes are incremented by 2 to avoid overlap. Nodes are described with *ccs* labels, rank number of architectures (1-6,162) and of SPX/SPAX extracted domains (1-1,643) in the phylogenomic tree (in curly braces), age and event (in curly braces). Node RGB colors represent age. Grey-scale color of node borders depict fusional/fissional properties. Node shapes describe GO categories.


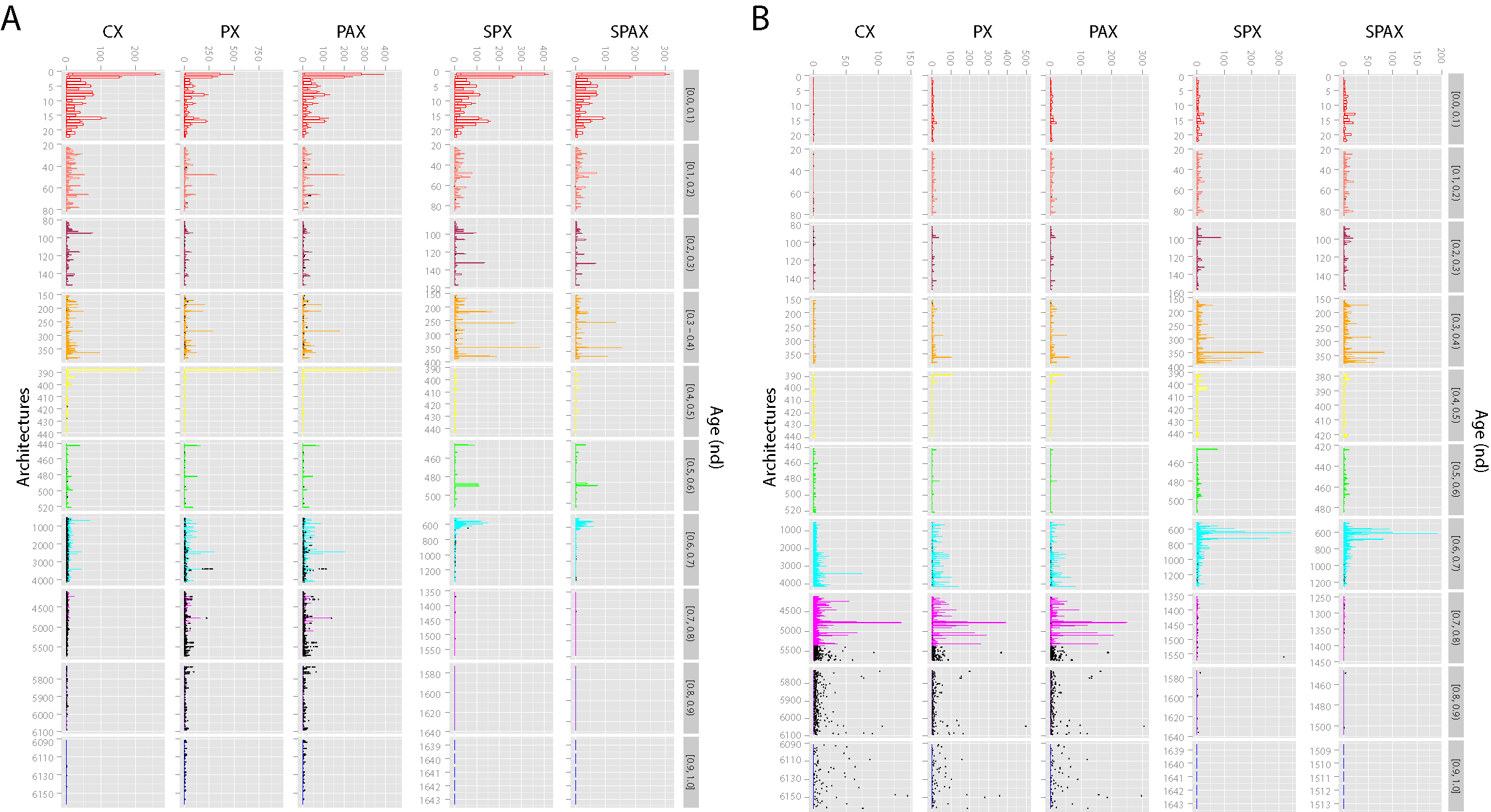


**Supplementary** **Figure S4. Boxplots of the cumulative weighted (A) outdegree and (B) indegree connectivity over the evolutionary timeline.** The Tukey boxplots show boxes (first and third quartiles bracketing the median), whiskers (values within ±1.5 × inner quartile range), and outliers (individual dots). The plots describe the span of connectivity given as expansion in node degree along the 169 events of CX, PX and PAX, and the 161 events of SPX and SPAX. They provide a view of chronological accumulation of connections (in the form of links or arcs) with time. Outdegree and indegree connectivity distributions are given separately for each network. The entries on the left scale of the plots correspond to the entire entity set of 6,162 architectures of the phylogenetic tree in case of CX, PX and PAX, and to 1,643 single domains mapped to the entity set of 6,162 architectures in case of SPX and SPAX. This scale was arranged top-down following chronological order of architectures (*nd*). The timeline of events was coarse-grained into 10 age bins (panels) and the boxplots were color coded according to age (Supplementary Figs. S2 and S3).


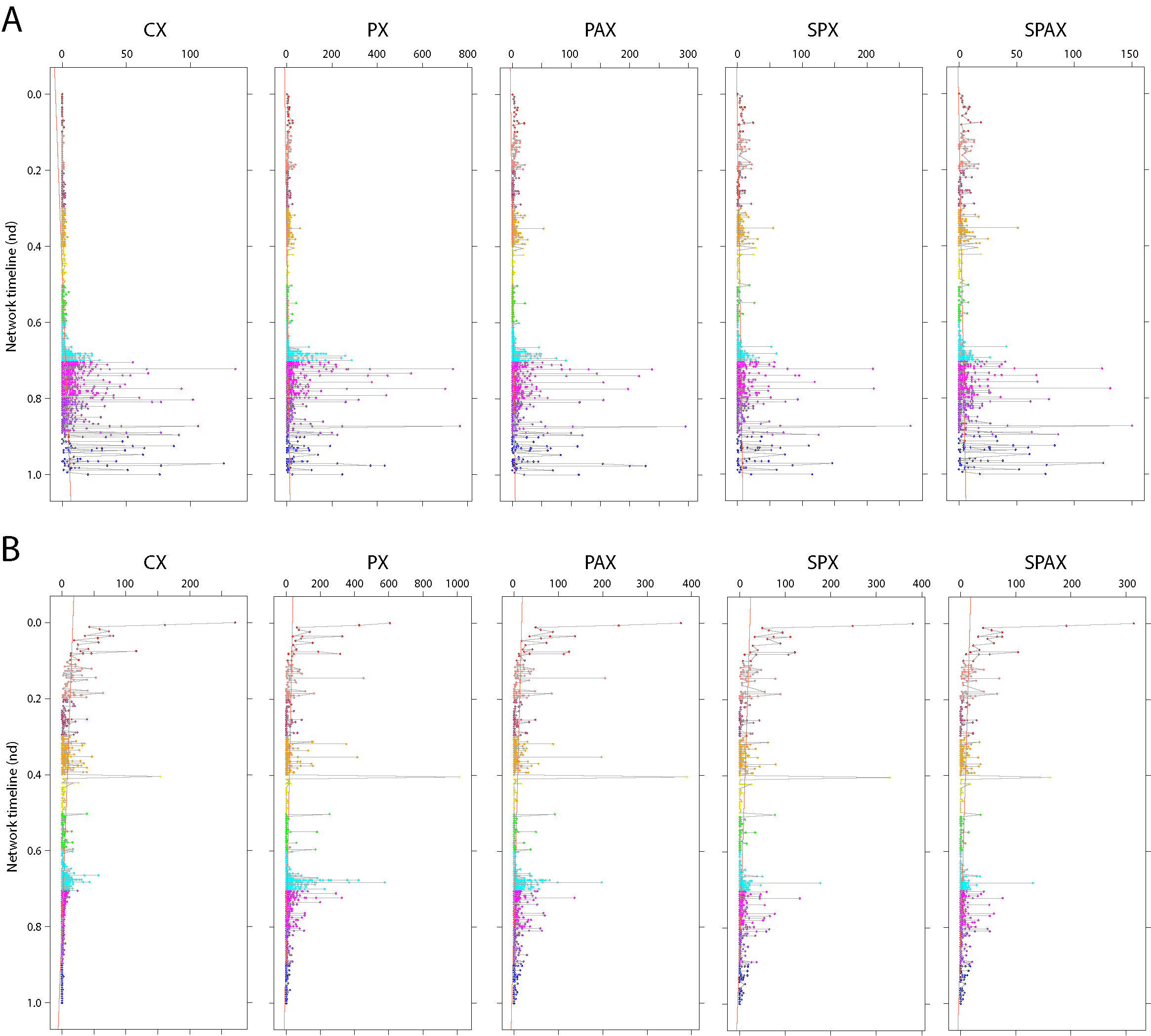


**Supplementary** **Figure S5. Connectivity of events along the timeline of evolving networks.** Data points describe indegree (**A**) and outdegree (**B**) of network nodes of different ages analyzed at present time (*nd* = 1). Symbols were color-coded according to node age. Linear regression lines (red) show a trend of growth in the embedding of old structures in emergent architectures (indegrees) and a decreasing trend of generating new architectures (outdegrees) as the process of protein cooption approaches modern times.


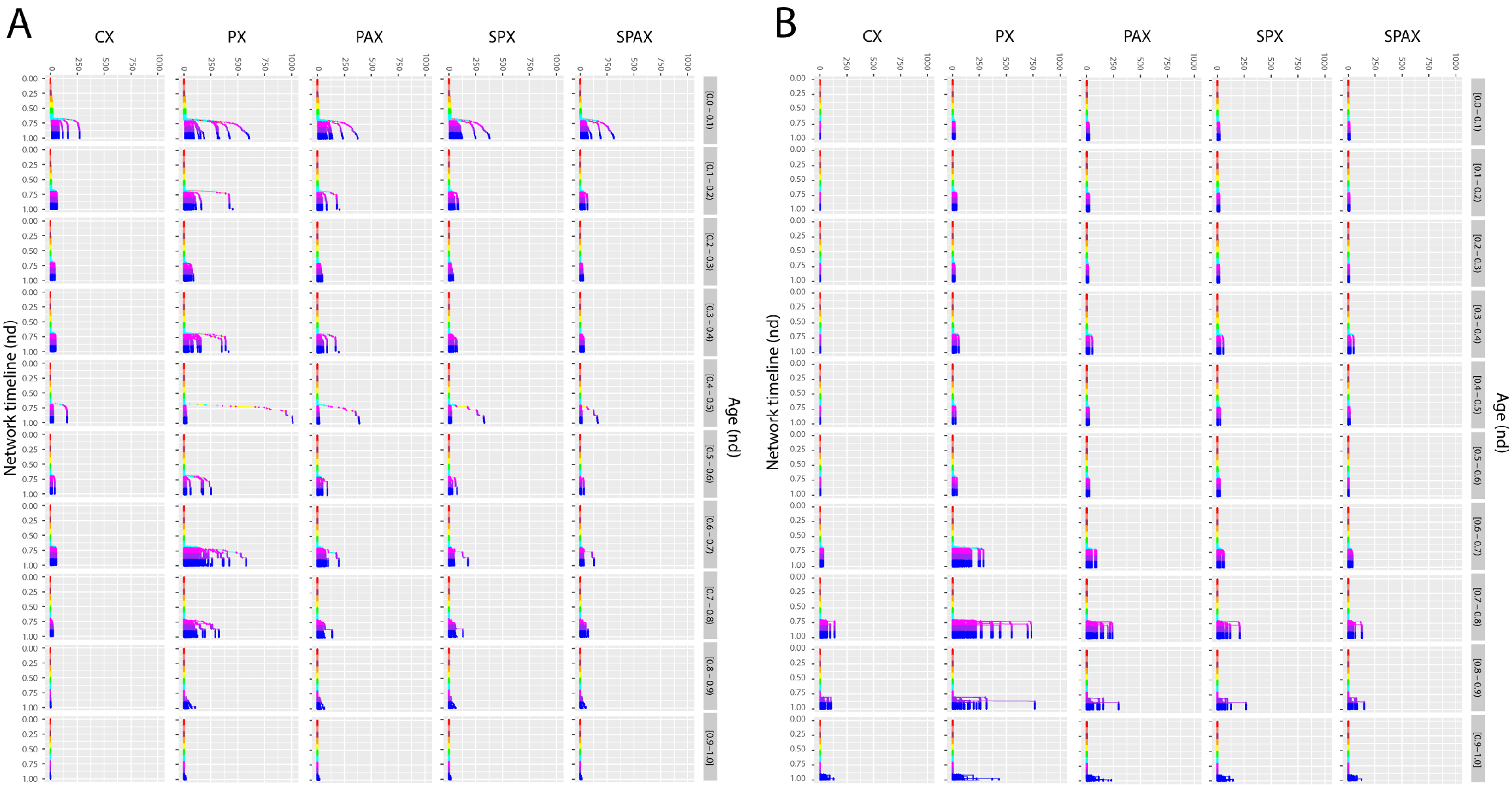


**Supplementary** **Figure S6. Scattergrams of accumulation of weighted outdegree (A) and indegree (B) over the evolutionary timeline.** The colored dots connected by individual lines (scattergrams) along the horizontal axis showcase degree accumulation in networks of protein domain organization distributed across the 169 (CX, PX and PAX) and 161 events (SPX and SPAX) of the evolutionary timeline, respectively. Scattergram panels from top to bottom follow the 10 age bins of domain organization. Their vertically oriented horizontal axes on the left represent normalized network timelines in relative 0-to-1 *nd* scales. The scattered degree points of each age-bin were apportioned from the entity sets of 6,162 architectures (CX, PX and PAX) and 1,643 single domains mapped to the entity set of 6,162 architectures (SPX and SPAX), respectively. The points are connected with curves, and both are color coded according to age (Supplementary Figs. S2 and S3) to illustrate source-sink relationships throughout the entire evolutionary timeline.


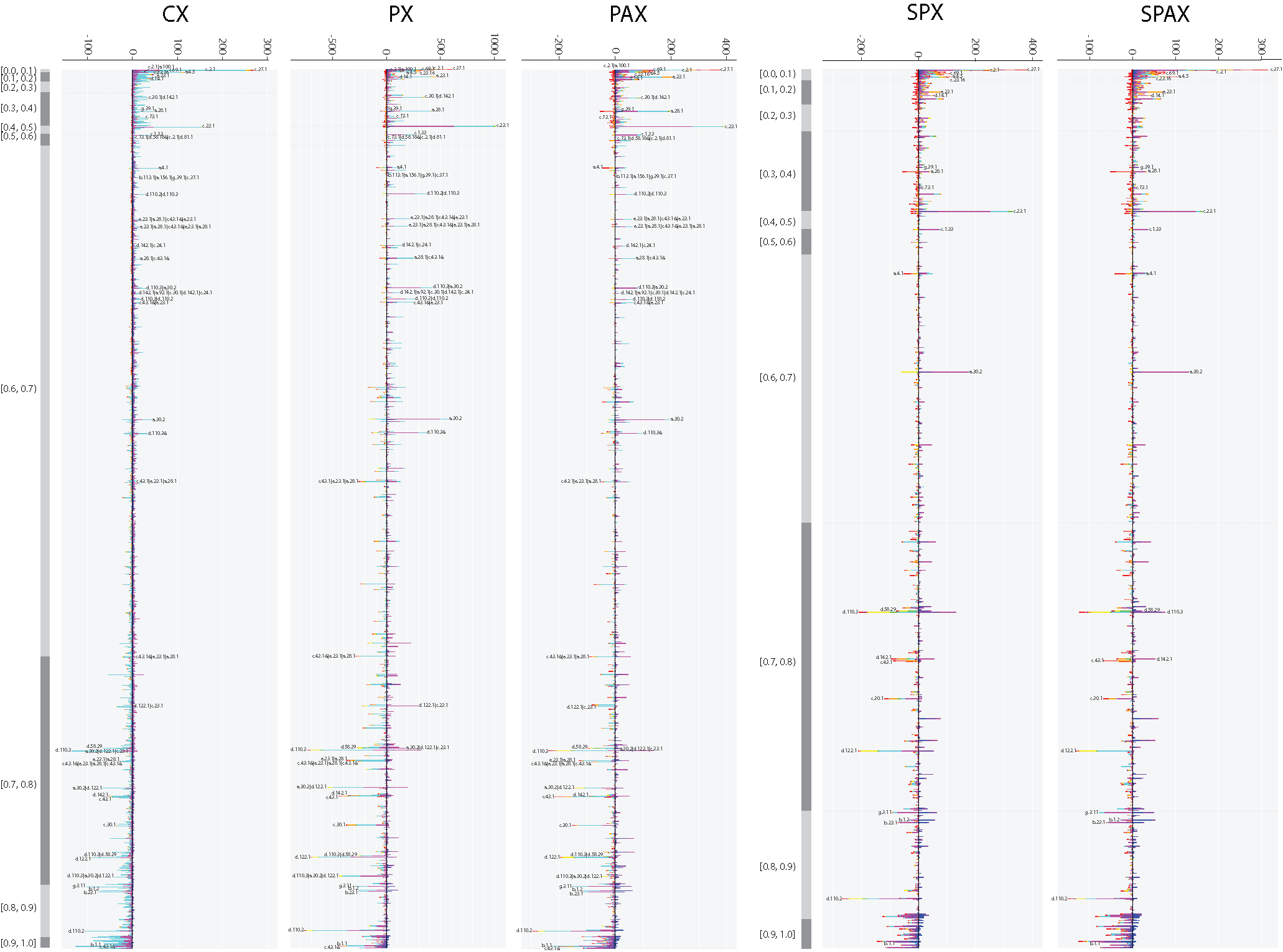


**Supplementary** **Figure S7. Chronological accumulation of connectivity in networks of protein domain organization.** The stacked bar charts depict the cumulative number of directed links (arcs) established in chronological order along the 169 or 161 events of architectural discovery that unfold along the evolutionary timeline of architectural innovation. This timeline, which spans a historical period from the origin (*nd* = 0) to the modern structural world of proteins (*nd* = 1), was coarse-grained into 10 age bins to help visualize the network data. Age bins of network nodes are illustrated on the left for the CX, PX and PAX networks and on the right for the SPX and SPAX networks. Not all architectures were labeled since they would not be legible. The labelled nodes include hub architectures within the 99.9th percentile of outdegree (Table1) or indegree (Table2) connectivity, and selected hub-like architectures (Supplementary Section 4 – Hubs in Network Evolution) with significantly high connectivity but below the 99.9th percentile. They total 49 and 25 representative nodes from the 6,162 and 1,643 nodes-sets, respectively. The various colored blocks of each stacked bar correspond to the 10 age bins (colored red-to-blue) of connectivity of an architecture in the evolving networks. The various sizes of stacked bars along the evolutionary timeline indicate node outdegree (positive values towards the right of the zero line) and indegree (negative values towards the left). This visualization that mimics a skyline of skyscrapers and could be termed as ‘degree-dispersion’ (in lieu of dispersion of light by a prism) showcases a comparative analysis of degree data of an evolving network. The stacked bar charts illustrate the sink-source relationships in architectural recruitment for the five evolving networks.


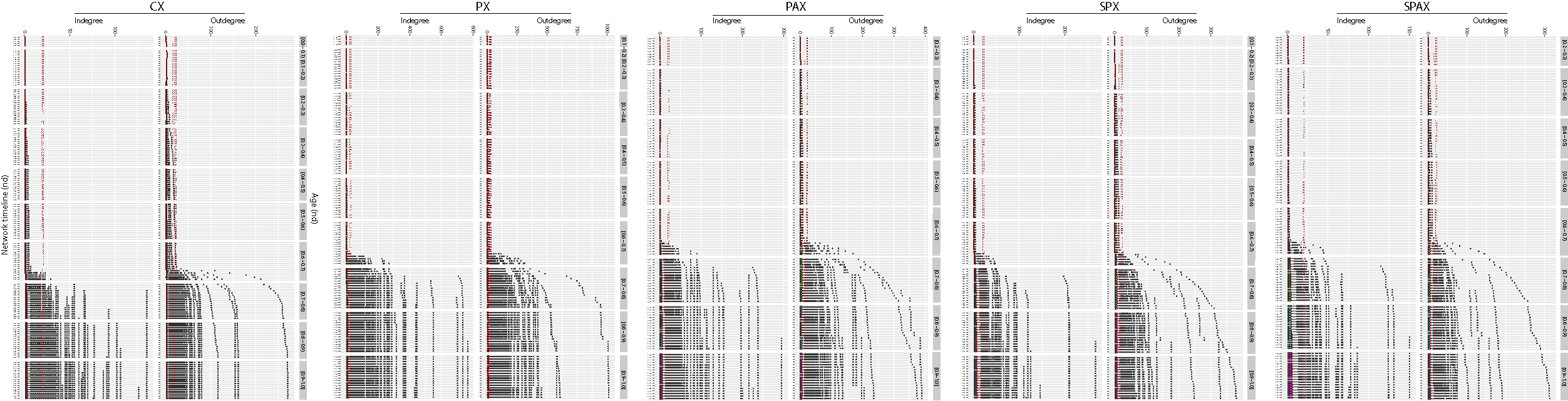


**Supplementary** **Figure S8. Measures of central tendency and dispersion in evolving networks.** The accumulation (and dispersion) of node degrees of evolving networks by age (*nd* value) was captured using Tukey boxplots with boxes (first and third quartiles bracketing the median), whiskers (values within ±1.5 × inner quartile range) and outliers (black dots). Each boxplot describes the span of cumulative indegrees or outdegrees along events of the evolutionary timeline by providing visualization of chronological accumulation of links with time. Average values of connectivity of architectures were mapped using red dots and reported with two digits of significance. Each of the 169 or 161 aggregate events along the timeline are labeled with their relative age in a 0-to-1 scale. The timeline of events was also coarse-grained into 10 age bins.


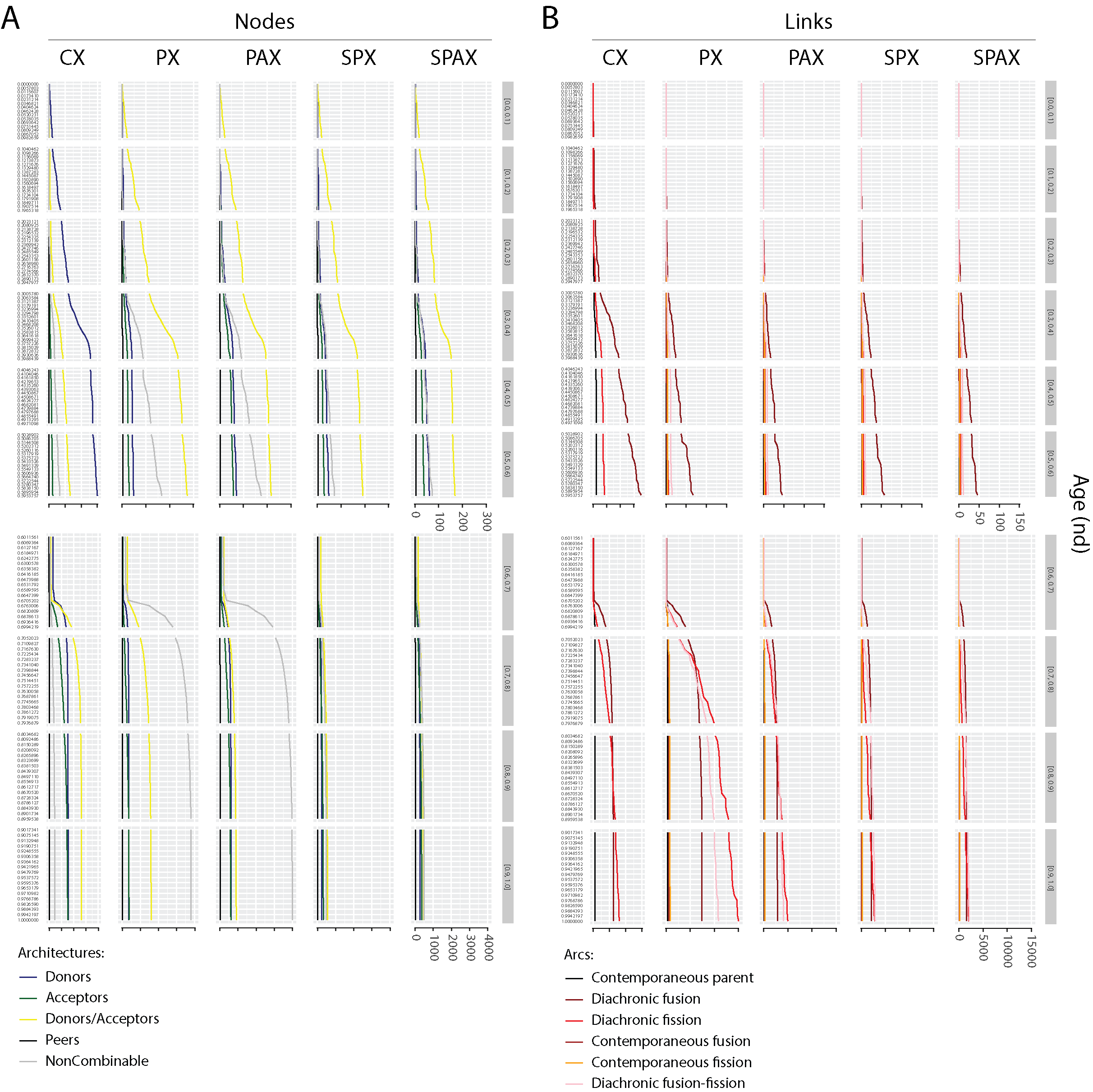


**Supplementary** **Figure S9. The accumulation of nodes (A) and links (B) in evolving networks.** The plots describe the cumulative count of nodes and arcs along the evolutionary timeline. Nodes represent architectures and arcs represent domain recruitment and rearrangement by fusion and fission processes (see inset of Supplementary Figure S2). Results illustrate how the accumulation of donors and the number of fusion events consistently increased during the initial evolutionary phase of domain organization. This trend shows dominance of the combinatorial interplay of domain organization in early molecular history. However, this fusion-based donor role was mostly confined to single domains and did not remain prominent during later evolutionary phases that followed the ‘big bang’ of domain combinations. During the later phases, multidomain proteins adopted the dual role of source and sink, primarily using fission processes to populate the modern eukaryotic world. A sigmoidal pattern around the big bang is vivid in all the plots. These patterns in the two phases of evolving networks confirms the fusional and fissional mechanics that tailored the structural history of proteins, as put forth by Wang and Caetano-Anollés (2009**)**.


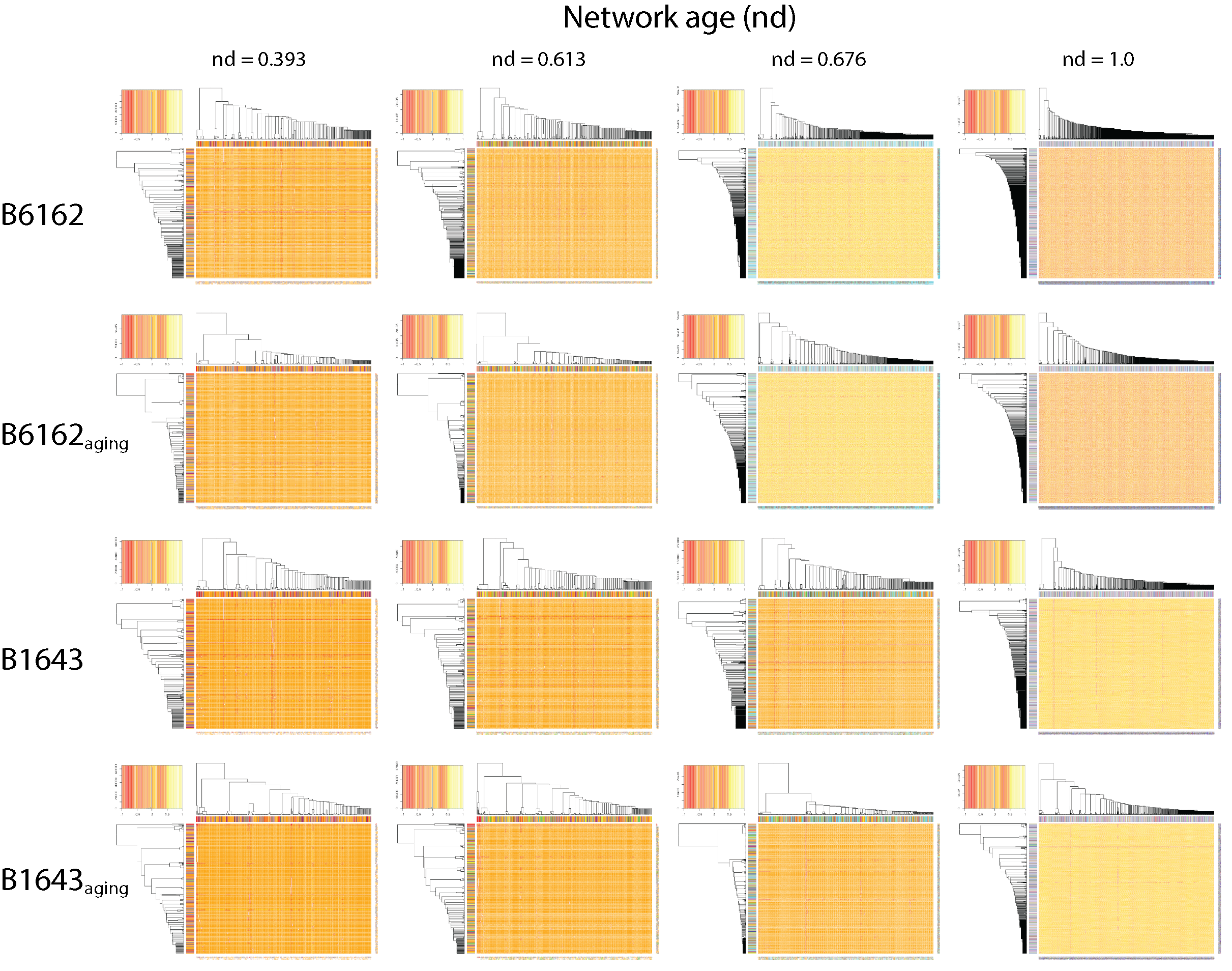


**Supplementary** **Figure S10.** **Modularity behavior observations of Barabási power-law controls over select events of the evolutionary timeline.** The connectivity of control networks was input to computation of NGage modularity matrices^65^ that were scaled by log10 of absolute network wide modularity values for calculation of Euclidean distance matrices^87^. The resulting heatmaps visually parametrize the distance matrices and show any modular strength between two nodes as compared to that of the rest of the network. The dendrograms were embedded using hierarchical clustering of the distance matrices with Ward’s minimum variance method^88^, where the height represents dissimilarity between clusters while the clades show the rearrangements of control nodes. The four comparative panels correspond to the approximate middle boundaries of the three epochs of evolutionary phylogenomic tree of domain organization at *nd* 0.393 and 0.612, the big bang of protein world at *nd* 0.676, and the fully-grown network at *nd* 1. In case of controls for SPX and SPAX, the node markers correspond to the 1,643 single domains mapped to the entity set of 6,162 architectures from the phylogenetic tree of proteins. The control nodes were age-sorted ascendingly within clusters. The color-coding of bands and labels is according to the age-based legend (Supplementary Fig. S2) and distinctively identifies their assigned ages. Both original Barabási and Barabási Aging controls elaborate modularity observations in power law networks with consistently systematic and predictable heatmap patterns, in comparison to those of the domain organization networks, with the signature long tail pattern of clustering becoming apparent in the control dendrograms as time progresses.

**Supplementary Tables**

Supplementary Table 1. Cumulative final counts per categories of nodes (domains, supradomains and multidomains) and connecting arcs in CX at age *nd* = 1. The composition network (CX) is a partial bipartite network that defines connectivity between domains, supradomains and multidomains. An algorithm was designed to mine the domains, supradomain and multidomain set for composition events. The algorithm’s time complexity was O(n^2) for comparisons across the domain, supradomain and multidomain categories, and O(n^2-n) for self-comparisons. The space complexity was <= 128MB and the algorithm took ~33 seconds to run. Please note that the appearance of a child domain/supradomain in a parent multidomain more than once was considered as only one presence per instance, e.g., c.36.1 in c.36.1|c.31.1|c.36.1 of 1a). Also, the appearance of a child domain in a parent multidomain with repeat showcases a double event in protein evolution with combination and repetition occurring at the same instance, e.g., c.37.1 in a.174.1|c.37.1& of 1c). Similarly, occurrence of a child supradomain in a parent multidomain with repeat represents evolutionarily a partial double event with combination and partial repetition (at the beginning or end of the child node) occurring at the same instance, e.g., c.2.1|a.100.1 in c.2.1|a.100.1& of 3a) and d.112.1&|d.94.1 in d.112.1&|d.94.1& of 4. Part of repeat matches happen when a carboxyl (C-terminus, 3' or the right most) or amino (N-terminus, 5' or the left most) end of a domain appears in a domain repeat (as single domain) or in a multidomain with repeats (as possibly part of a multidomain). Investigation of the part of repeat matches due to the appearance of a non-terminal domain in the non-terminal domain repeat of a parent multidomain were eluded because these matches failed to fulfill the contiguity condition of composition. Also, it should be noted that the presence of a complete match was given priority over that of a part of repeat match.

| 1a) Domains in multidomains with no repeats (1,513 x 3,484 = 5,271,292 comparisons) | | | |  |
| --- | --- | --- | --- | --- |
|  | Arcs with part of repeat match | Arcs with complete match | Arcs Total | |
| Contemporaneous | 0 | 133 | 133 | |
| Diachronic: Fusion | 0 | 3,701 c.37.1 (0.0000000) -> f.37.1\|c.37.1 (0.1271676) c.37.1 (0.0000000) -> c.37.1\|b.43.3\|d.58.11\|d.14.1\|d.58.11 (0.1791908) c.37.1 (0.0000000) -> c.37.1\|b.43.3\|b.44.1 (0.1965318) | 3,701 | |
| Fission | 0 | 4,049 c.3.1\|d.87.1 (0.1560694) -> c.3.1 (0.1849711) c.36.1\|c.31.1\|c.36.1 (0.2023121) -> c.31.1 (0.2543353) c.36.1\|c.31.1\|c.36.1 (0.2023121) -> c.36.1 (0.2601156) | 4,049 | |
| Sum total | 0 | 7,883 | 7,883 | |
| 1b) Domains in domain repeats (1,513 x 387 = 585,531 comparisons) | | |  | |
|  | Arcs with part of repeat match | Arcs with complete match | Arcs Total | |
| Contemporaneous | 13 | 0 | 13 | |
| Diachronic: Fusion | 168 c.37.1 (0.0000000) -> c.37.1& (0.1907514) c.2.1 (0.0057803) -> c.2.1& (0.7398844) c.67.1 (0.0115607) -> c.67.1& (0.6878613) | 0 | 168 | |
| Fission | 201 c.55.1& (0.0462428) -> c.55.1 (0.1907514) c.95.1& (0.1387283) -> c.95.1 (0.3583815) d.58.53& (0.6416185) -> d.58.53 (0.6473988) | 0 | 201 | |
| Sum total | 382 | 0 | 382 | |
| 1c) Domains in multidomains with repeats (1,513 x 778 = 1,177,114 comparisons) | | | |  |
|  | Arcs with part of repeat match | Arcs with complete match | Arcs Total | |
| Contemporaneous | 15 | 23 | 38 | |
| Diachronic: Fusion | 205 c.37.1 (0.0000000) -> a.174.1\|c.37.1& (0.3121387) c.37.1 (0.0000000) -> c.37.1&\|c.52.1 (0.4682081) c.37.1 (0.0000000) -> c.37.1&\|b.34.18\|c.37.1&\|d.315.1 (0.4913295) | 420 c.37.1 (0.0000000) -> c.37.1\|h.1.26& (0.6647399) c.37.1 (0.0000000) -> a.60.4&\|c.37.1 (0.6763006) c.37.1 (0.0000000) -> c.37.1\|c.55.1& (0.6763006) | 625 | |
| Fission | 541 c.36.1&\|c.48.1 (0.1849711) -> c.36.1 (0.2601156) a.4.1&\|d.60.1 (0.5491329) -> a.4.1 (0.6647399) b.82.4\|a.4.1& (0.5549133) -> a.4.1 (0.6647399) | 705 c.73.1\|d.58.18& (0.3179191) -> c.73.1 (0.3641618) c.37.1&\|b.34.18\|c.37.1&\|d.315.1 (0.4913295) -> d.315.1 (0.6763006) c.55.1&\|d.22.1 (0.6647399) -> d.22.1 (0.6763006) | 1,246 | |
| Sum total | 761 | 1,148 | 1,909 | |
| 2) Domain repeats in multidomains with repeats (387 x 778 = 301,086 comparisons) | | | |  |
|  | Arcs with part of repeat match | Arcs with complete match | Arcs Total | |
| Contemporaneous | 0 | 21 | 21 | |
| Diachronic: Fusion | 0 | 210 c.55.1& (0.0462428) -> c.55.1&\|a.211.1 (0.4450867) c.55.1& (0.0462428) -> c.55.1&\|d.22.1 (0.6647399) c.55.1& (0.0462428) -> c.66.1\|c.55.1& (0.6763006) | 210 | |
| Fission | 0 | 543 c.73.1\|d.58.18& (0.3179191) -> d.58.18& (0.3294798) a.4.1&\|d.60.1 (0.5491329) -> a.4.1& (0.6011561) b.82.4\|a.4.1& (0.5549133) -> a.4.1& (0.6011561) | 543 | |
| Sum total | 0 | 774 | 774 | |
| 3a) Multidomains with no repeats in themselves (3,484-1 x 3,484 = 12,134,772 comparisons) | | | |  |
|  | Arcs with part of repeat match | Arcs with complete match | Arcs Total | |
| Contemporaneous | 0 | 165 | 165 | |
| Diachronic: Fusion | 0 | 943 c.2.1\|a.100.1 (0.0693642) -> c.2.1\|a.100.1\|c.26.3 (0.3352601) c.2.1\|a.100.1 (0.0693642) -> c.37.1\|c.2.1\|a.100.1 (0.6705202) c.2.1\|a.100.1 (0.0693642) -> c.111.1\|c.2.1\|a.100.1 (0.6763006) | 943 | |
| Fission | 0 | 1,023 d.67.2\|c.26.1\|a.27.1 (0.2312139) -> c.26.1\|a.27.1 (0.2658960) c.30.1\|d.142.1\|a.92.1\|c.30.1\|d.142.1\|c.24.1 (0.3121387) -> c.30.1\|d.142.1 (0.3179191) c.23.1\|c.37.1\|a.4.1 (0.3699422) -> c.37.1\|a.4.1 (0.5780347) | 1,023 | |
| Sum total | 0 | 2,131 | 2,131 | |
| 3b) Multidomains with no repeats in multidomains with repeats (3,484 x 778 = 2,710,552 comparisons) | | | |  |
|  | Arcs with part of repeat match | Arcs with complete match | Arcs Total | |
| Contemporaneous | 84 | 36 | 120 | |
| Diachronic: Fusion | 246 c.2.1\|a.100.1 (0.0693642) -> c.2.1\|a.100.1& (0.5086705) c.2.1\|a.100.1 (0.0693642) -> c.14.1\|c.2.1\|a.100.1& (0.6416185) c.2.1\|a.100.1 (0.0693642) -> c.2.1\|a.100.1&\|c.14.1 (0.7167630) | 86 c.2.1\|d.81.1 (0.0867052) -> c.73.1\|d.58.18&\|c.2.1\|d.81.1 (0.5202312) c.2.1\|d.81.1 (0.0867052) -> c.2.1\|d.81.1\|c.73.1\|d.58.18& (0.6936416) c.3.1\|d.87.1 (0.1560694) -> b.84.1&\|a.9.1\|c.43.1\|c.3.1\|d.87.1 (0.6763006) | 332 | |
| Fission | 400 c.36.1&\|c.48.1 (0.1849711) -> c.36.1\|c.48.1 (0.2601156) a.177.1\|a.4.13& (0.3757226) -> a.177.1\|a.4.13 (0.3988439) c.37.1&\|c.52.1 (0.4682081) -> c.37.1\|c.52.1 (0.6705202) | 230 c.62.1\|g.3.11\|g.18.1& (0.6647399) -> c.62.1\|g.3.11 (0.6705202) d.110.3&\|d.110.2\|d.110.3\|d.110.2\|a.30.2\|d.122.1\|c.23.1 (0.6705202) -> d.110.2\|d.110.3 (0.6763006) d.110.3&\|d.110.2\|d.110.3\|a.30.2\|d.122.1 (0.6705202) -> d.110.2\|d.110.3 (0.6763006) | 630 | |
| Sum total | 730 | 352 | 1,082 | |
| 4) Multidomains with repeats in themselves (778-1 x 778 = 604,506 comparisons) | | |  | |
|  | Arcs with part of repeat match | Arcs with complete match | Arcs Total | |
| Contemporaneous | 27 | 94 | 121 | |
| Diachronic: Fusion | 42 d.112.1&\|d.94.1 (0.5433526) -> d.112.1&\|d.94.1& (0.6763006) b.23.1&\|g.12.1 (0.6589595) -> d.58.41\|b.23.1&\|g.12.1&\|b.47.1 (0.6994219) b.23.1&\|g.12.1 (0.6589595) -> b.23.1&\|g.12.1&\|b.47.1 (0.7167630) | 178 c.84.1&\|d.129.2 (0.1676301) -> c.68.1\|c.84.1&\|d.129.2 (0.6763006) c.84.1&\|d.129.2 (0.1676301) -> c.84.1&\|d.129.2\|c.68.1\|b.82.1 (0.6763006) c.84.1&\|d.129.2 (0.1676301) -> c.68.1\|b.81.1\|c.84.1&\|d.129.2 (0.6936416) | 220 | |
| Fission | 55 g.12.1&\|b.23.1& (0.6589595) -> g.12.1&\|b.23.1 (0.6647399) g.3.11&\|b.29.1&\|g.3.11& (0.6647399) -> b.29.1\|g.3.11& (0.6763006) g.12.1\|g.3.14\|g.12.1&\|g.3.11& (0.6647399) -> g.12.1&\|g.3.11 (0.6763006) | 286 c.37.1&\|b.34.18\|c.37.1&\|d.315.1 (0.4913295) -> c.37.1&\|b.34.18 (0.6763006) c.62.1\|g.3.11\|g.18.1& (0.6647399) -> g.3.11\|g.18.1& (0.6763006) g.10.1\|b.29.1\|g.3.11\|g.18.1&\|b.1.2&\|c.45.1& (0.6647399) -> g.3.11\|g.18.1& (0.6763006) | 341 | |
| Sum total | 124 | 558 | 682 | |
| Grand total | 1,997 | 12,846 | 14,843 | |
| 5) Domain Categories | | | | |
| Donors | 1,545 - c.37.1 (0.0000000); c.2.1 (0.0057803); c.67.1 (0.0115607) | | | |
| Acceptors | 1,518 - c.37.1\|c.23.16 (0.2369942); d.153.1\|c.80.1 (0.3005780); c.26.1\|d.66.1 (0.3179191) | | | |
| Donors/Acceptors | 2,628 - c.2.1\|a.100.1 (0.0693642); c.2.1\|d.81.1 (0.0867052); c.58.1\|c.2.1 (0.1098266) | | | |
| Peers | 21 - d.2.1& (0.6647399); a.172.1 (0.6763006); a.4.14 (0.6763006) | | | |
| Noncombinables | 450 - f.41.1 (0.2312139); a.75.1 (0.2427746); d.55.1 (0.2427746) | | | |

Supplementary Table 2: Cumulative final counts per categories of nodes (domains, supradomains and multidomains) and connecting arcs in PX at age *nd* = 1. The pairwise network (PX) is a projection-like of CX and defines connectivity between domains, supradomains and multidomains based on pairwise events in the same or other proteins. The algorithm designed to generate the domain/supradomain pairs originally had a time complexity of O(n^3). The time complexity was reduced to O((n^2-n)/2) by generating parent multidomain arrays and utilizing set intersection. The space complexity of the algorithm was <= 146MB and it took ~66 seconds to execute. A parent node was defined as a multidomain that was composed by at least two child domains/supradomains through a complete or a part of repeat match, with priority given to the complete match type. Additionally, both the child domains/supradomains in a pair were required to match completely in the parent multidomain to specify a complete match of the pair. However, a part of repeat match of a pair could be specified by part of repeat match of any child. A fusion event was specified when a parent multidomain was younger than or of the same age as the younger child in the domain/supradomain pair. Similarly, a fission event was specified when a parent multidomain was older than or of the same age as the older child in the domain/supradomain pair. However, a fusion/fission event was specified when a parent multidomain's age was between the ages of the two child domains/supradomains in the pair, possibly equal to any child node’s age. Self- pairwise events due to multiple appearances of a child domain/supradomain in a parent multidomain were not included as arcs because such duplications were considered as a single appearance in the timeline. Similarly, arcs that represented self-pairwise links of domains in domain repeats were considered redundant and hence excluded. Also, the additional composition events within the child domain/supradomain pairs represented the subset of arcs borrowed from CX and were considered as overlap. Furthermore, fusion overlaps were the overlap events when an older sibling domain/supradomain composed the younger sibling supradomain, while fission overlaps occurred when a younger sibling domain/supradomain composed the older sibling supradomain. The events when an overlap occurred in addition to an independent appearance of a child domain/supradomain pair in the parent multidomain were included in the network but not counted separately. Similarly, events of partial overlap of a child domain/supradomain at the carboxyl or amino end of the child supradomain in the pair were also not counted separately. Furthermore, both the "part of repeat" and "complete" match types were detected in each of the fusion and fission overlap events but were not counted separately. Noticeably, counting such events separately would require use of regular expressions and text alignments, an investigation considered as out of scope of the current study where we focus on mining global evolutionary patterns of structural motifs of domain organization.

| Self-set comparisons | | | |
| --- | --- | --- | --- |
| 1a) Domains in multidomains with no repeats (3,483 /\ 3,483 = 3,483 parents with 3,364 common) | | | |
|  | Arcs with part of repeat match | Arcs with complete match | Arcs Total |
| Contemporaneous: Fusion | 0 | 41 | 41 |
| Fission | 0 | 113 | 113 |
| Fusion/Fission | 0 | 4 | 4 |
| Diachronic: Fusion | 0 | 1,583 c.37.1 (0.0000000) -> d.14.1 (0.1791908): c.37.1\|b.43.3\|d.58.11\|d.14.1\|d.58.11 (0.1791908) c.37.1 (0.0000000) -> c.23.16 (0.0809249): c.37.1\|c.23.16 (0.2369942) c.37.1 (0.0000000) -> a.4.5 (0.0751445): c.37.1\|a.4.5 (0.2716763) | 1,583 |
| Fission | 0 | 1,928 c.31.1 (0.2543353) -> c.36.1 (0.2601156): c.36.1\|c.31.1\|c.36.1 (0.2023121) d.14.1 (0.1791908) -> b.43.3 (0.2890173): c.37.1\|b.43.3\|d.58.11\|d.14.1\|d.58.11 (0.1791908) c.116.1 (0.3526012) -> b.122.1 (0.3641618): b.122.1\|c.116.1 (0.3294798) | 1,928 |
| Fusion/Fission | 0 | 2,113 c.37.1 (0.0000000) -> b.43.3 (0.2890173): c.37.1\|b.43.3\|d.58.11\|d.14.1\|d.58.11 (0.1791908) c.37.1 (0.0000000) -> b.43.3 (0.2890173): c.37.1\|b.43.3\|b.44.1 (0.1965318) c.37.1 (0.0000000) -> g.39.1 (0.3468208): g.39.1\|c.37.1 (0.3121387) | 2,113 |
| Sum total | 0 | 5,782 | 5,782 |
| 1b) Domains in multidomains with repeats (777 /\ 777 = 777 parents with 765 common) | | | |
|  | Arcs with part of repeat match | Arcs with complete match | Arcs Total |
| Contemporaneous: Fusion | 4 | 2 | 6 |
| Fission | 30 | 13 | 43 |
| Fusion/Fission | 0 | 1 | 1 |
| Diachronic: Fusion | 158 b.40.4 (0.0520231) -> b.34.5 (0.1849711): b.34.5\|b.40.4& (0.3005780) c.55.1 (0.1907514) -> a.211.1 (0.2543353): c.55.1&\|a.211.1 (0.4450867) c.47.1 (0.0231214) -> c.3.1 (0.1849711): c.47.1&\|c.3.1 (0.4508671) | 108 c.2.1 (0.0057803) -> c.73.1 (0.3641618): c.73.1\|d.58.18&\|c.2.1\|d.81.1 (0.5202312) c.69.1 (0.0346821) -> e.23.1 (0.1445087): c.43.1&\|e.23.1\|a.28.1\|c.69.1 (0.5953757) c.69.1 (0.0346821) -> a.28.1 (0.3526012): c.43.1&\|e.23.1\|a.28.1\|c.69.1 (0.5953757) | 266 |
| Fission | 493 a.4.1 (0.6647399) -> b.82.4 (0.6878613): b.82.4\|a.4.1& (0.5549133) b.1.3 (0.6820809) -> c.6.1 (0.6878613): b.2.2\|b.1.3&\|c.6.1 (0.6820809) a.118.14 (0.6820809) -> d.37.1 (0.6936416): a.118.14\|d.37.1& (0.6763006) | 241 c.142.1 (0.6763006) -> d.15.13 (0.6820809): c.47.1&\|c.142.1\|d.15.13\|a.29.12 (0.6763006) a.29.12 (0.6763006) -> d.15.13 (0.6820809): c.47.1&\|c.142.1\|d.15.13\|a.29.12 (0.6763006) d.142.2 (0.6878613) -> a.60.2 (0.6936416): d.142.2\|b.40.4\|a.60.2\|c.15.1& (0.6878613) | 734 |
| Fusion/Fission | 392 c.37.1 (0.0000000) -> d.315.1 (0.6763006): c.37.1&\|b.34.18\|c.37.1&\|d.315.1 (0.4913295) c.55.1 (0.1907514) -> d.22.1 (0.6763006): c.55.1&\|d.22.1 (0.6647399) b.40.4 (0.0520231) -> a.60.4 (0.6820809): d.202.1\|b.40.4\|d.52.3&\|a.60.4& (0.4219653) | 198 c.23.1 (0.4046243) -> a.30.2 (0.6820809): d.110.3&\|d.110.2\|d.110.3\|d.110.2\|a.30.2\|d.122.1\|c.23.1 (0.6705202) c.23.1 (0.4046243) -> a.30.2 (0.6820809): d.110.3\|d.110.2&\|a.30.2\|d.122.1\|c.23.1 (0.6763006) c.23.1 (0.4046243) -> a.30.2 (0.6820809): d.110.2\|d.110.3&\|a.30.2\|d.122.1\|c.23.1 (0.6763006) | 590 |
| Sum total | 1,077 | 563 | 1,640 |
| 2) Domain repeats in multidomains with repeats (720 /\ 720 = 720 parents with 53 common) | | | |
|  | Arcs with part of repeat match | Arcs with complete match | Arcs Total |
| Contemporaneous: Fusion | 0 | 1 | 1 |
| Fission | 0 | 4 | 4 |
| Fusion/Fission | 0 | 0 | 0 |
| Diachronic: Fusion | 0 | 3 d.37.1& (0.3641618) -> d.110.3& (0.6820809): d.37.1&\|d.110.3&\|c.37.1\|a.4.1 (0.6820809) c.55.1& (0.0462428) -> a.8.4& (0.6647399): c.55.1&\|b.130.1\|a.8.4& (0.6878613) d.37.1& (0.3641618) -> c.26.2& (0.4682081): f.20.1\|d.37.1&\|c.26.2& (0.7225434) | 3 |
| Fission | 0 | 38 d.106.1& (0.6763006) -> d.38.1& (0.6820809): c.2.1\|d.38.1&\|d.106.1& (0.6647399) d.110.3& (0.6820809) -> c.23.1& (0.7283237): c.23.1&\|d.110.2\|d.110.3&\|d.58.29\|c.1.33 (0.6763006) d.110.3& (0.6820809) -> c.23.1& (0.7283237): c.23.1\|d.110.2\|d.110.3&\|a.30.2\|d.122.1\|c.23.1&\|a.24.10 (0.6763006) | 38 |
| Fusion/Fission | 0 | 9 d.37.1& (0.3641618) -> d.110.3& (0.6820809): d.37.1&\|d.110.3&\|d.58.29\|c.1.33 (0.6763006) d.110.3& (0.6820809) -> c.23.1& (0.7283237): c.23.1&\|d.110.3&\|a.30.2\|d.122.1 (0.6878613) d.110.3& (0.6820809) -> c.23.1& (0.7283237): c.23.1\|d.110.3&\|a.30.2\|d.122.1\|c.23.1& (0.6878613) | 9 |
| Sum total | 0 | 55 | 55 |
| 3a) Multidomains with no repeats in themselves (908 /\ 908 = 908 parents with 507 common) | | | |
|  | Arcs with part of repeat match | Arcs with complete match | Arcs Total |
| Contemporaneous: Fusion | 0 | 130 | 130 |
| Fission | 0 | 93 | 93 |
| Fusion/Fission | 0 | 36 | 36 |
| Overlap(s) | 131 | | |
| Diachronic: Fusion | 0 | 1,100 g.39.1\|c.37.1 (0.3121387) -> b.113.1\|a.156.1\|g.39.1 (0.3468208): b.113.1\|a.156.1\|g.39.1\|c.37.1 (0.6705202) d.79.4\|d.139.1 (0.3757226) -> d.139.1\|d.79.4 (0.6763006): d.79.4\|d.139.1\|d.79.4 (0.6763006) d.79.4\|d.139.1 (0.3757226) -> d.79.4\|d.139.1\|d.79.4 (0.6763006): a.5.10\|d.79.4\|d.139.1\|d.79.4 (0.6763006) | 1,100 |
| Fission | 0 | 1,560 c.30.1\|d.142.1 (0.3179191) -> a.92.1\|c.30.1\|d.142.1 (0.6705202): c.30.1\|d.142.1\|a.92.1\|c.30.1\|d.142.1\|c.24.1 (0.3121387) c.30.1\|d.142.1 (0.3179191) -> d.142.1\|c.24.1 (0.6763006): c.30.1\|d.142.1\|a.92.1\|c.30.1\|d.142.1\|c.24.1 (0.3121387) a.92.1\|c.30.1\|d.142.1 (0.6705202) -> d.142.1\|c.24.1 (0.6763006): c.30.1\|d.142.1\|a.92.1\|c.30.1\|d.142.1\|c.24.1 (0.3121387) | 1,560 |
| Fusion/Fission | 0 | 1,196 d.79.4\|d.139.1 (0.3757226) -> d.284.1\|a.5.10\|d.79.4\|d.139.1 (0.6705202): d.284.1\|a.5.10\|d.79.4\|d.139.1\|d.79.4\|d.139.1\|c.23.16 (0.4219653) d.14.1\|d.101.1 (0.2716763) -> d.14.1\|d.101.1\|d.51.1\|b.40.4 (0.6763006): d.14.1\|d.101.1\|a.4.9\|d.14.1\|d.101.1\|d.51.1\|b.40.4 (0.3352601) d.14.1\|d.101.1 (0.2716763) -> d.14.1\|d.101.1\|a.4.9\|d.14.1\|d.101.1\|d.51.1 (0.6763006): d.14.1\|d.101.1\|a.4.9\|d.14.1\|d.101.1\|d.51.1\|b.40.4 (0.3352601) | 1,196 |
| Overlap(s) | Fusion: 902 c.30.1\|d.142.1 (0.3179191) -> a.92.1\|c.30.1\|d.142.1 (0.6705202): c.30.1\|d.142.1\|a.92.1\|c.30.1\|d.142.1\|c.24.1 (0.3121387) d.79.4\|d.139.1 (0.3757226) -> d.284.1\|a.5.10\|d.79.4\|d.139.1 (0.6705202): d.284.1\|a.5.10\|d.79.4\|d.139.1\|d.79.4\|d.139.1\|c.23.16 (0.4219653) c.30.1\|d.142.1 (0.3179191) -> d.142.1\|a.92.1\|c.30.1\|d.142.1\|c.24.1 (0.6763006): c.30.1\|d.142.1\|a.92.1\|c.30.1\|d.142.1\|c.24.1 (0.3121387) | Fission: 771 b.113.1\|a.156.1\|g.39.1 (0.3468208) -> b.113.1\|a.156.1 (0.6763006): b.113.1\|a.156.1\|g.39.1\|c.37.1 (0.6705202) d.284.1\|a.5.10\|d.79.4\|d.139.1\|d.79.4\|d.139.1\|c.23.16 (0.4219653) -> d.284.1\|a.5.10\|d.79.4\|d.139.1 (0.6705202): c.2.1\|d.284.1\|a.5.10\|d.79.4\|d.139.1\|d.79.4\|d.139.1\|c.23.16 (0.6763006) d.284.1\|a.5.10\|d.79.4\|d.139.1\|d.79.4\|d.139.1\|c.23.16 (0.4219653) -> d.79.4\|d.139.1\|d.79.4 (0.6763006): c.2.1\|d.284.1\|a.5.10\|d.79.4\|d.139.1\|d.79.4\|d.139.1\|c.23.16 (0.6763006) | 1,673 |
| Sum total | 0 | 4,115 | 4,115 |
| 3b) Multidomains with no repeats in multidomains with repeats (535 /\ 535 = 535 parents with 219 common) | | | |
|  | Arcs with part of repeat match | Arcs with complete match | Arcs Total |
| Contemporaneous: Fusion | 26 | 8 | 34 |
| Fission | 43 | 14 | 57 |
| Fusion/Fission | 16 | 9 | 25 |
| Overlap(s) | 52 | | |
| Diachronic: Fusion | 141 c.3.1\|d.87.1 (0.1560694) -> b.84.1\|a.9.1\|c.43.1 (0.3930636): b.84.1&\|a.9.1\|c.43.1\|c.3.1\|d.87.1 (0.6763006) c.3.1\|d.87.1 (0.1560694) -> b.84.1\|a.9.1\|c.43.1\|c.3.1\|d.87.1 (0.6763006): b.84.1&\|a.9.1\|c.43.1\|c.3.1\|d.87.1 (0.6763006) b.84.1\|a.9.1\|c.43.1 (0.3930636) -> b.84.1\|a.9.1\|c.43.1\|c.3.1\|d.87.1 (0.6763006): b.84.1&\|a.9.1\|c.43.1\|c.3.1\|d.87.1 (0.6763006) | 25 c.30.1\|d.142.1 (0.3179191) -> c.30.1\|d.142.1\|b.84.2 (0.3526012): c.30.1\|d.142.1\|b.84.2\|b.84.1\|c.14.1& (0.6878613) c.30.1\|d.142.1 (0.3179191) -> d.142.1\|b.84.2 (0.6878613): c.30.1\|d.142.1\|b.84.2\|b.84.1\|c.14.1& (0.6878613) c.30.1\|d.142.1\|b.84.2 (0.3526012) -> d.142.1\|b.84.2 (0.6878613): c.30.1\|d.142.1\|b.84.2\|b.84.1\|c.14.1& (0.6878613) | 166 |
| Fission | 620 c.43.1\|e.23.1\|a.28.1\|c.69.1 (0.6763006) -> c.43.1\|e.23.1 (0.6820809): c.43.1&\|e.23.1\|a.28.1\|c.69.1 (0.5953757) c.43.1\|e.23.1\|a.28.1\|c.69.1 (0.6763006) -> e.23.1\|a.28.1\|c.69.1 (0.6820809): c.43.1&\|e.23.1\|a.28.1\|c.69.1 (0.5953757) c.43.1\|e.23.1\|a.28.1\|c.69.1 (0.6763006) -> a.28.1\|c.69.1 (0.6820809): c.43.1&\|e.23.1\|a.28.1\|c.69.1 (0.5953757) | 226 g.18.1\|b.23.1\|g.18.1 (0.6647399) -> b.23.1\|g.18.1\|b.23.1 (0.6763006): b.23.1\|g.18.1\|b.23.1\|g.18.1\|b.23.1\|g.18.1& (0.6647399) g.18.1\|b.23.1\|g.18.1 (0.6647399) -> b.23.1\|g.18.1\|b.23.1\|g.18.1\|b.23.1 (0.6763006): b.23.1\|g.18.1\|b.23.1\|g.18.1\|b.23.1\|g.18.1& (0.6647399) b.68.5\|g.3.11\|b.68.5 (0.6763006) -> b.68.5\|g.3.11 (0.6820809): b.68.5\|g.3.11\|b.68.5\|g.3.11\|g.12.1& (0.6763006) | 846 |
| Fusion/Fission | 218 b.84.1\|a.9.1\|c.43.1 (0.3930636) -> a.9.1\|c.43.1 (0.6820809): b.84.1&\|a.9.1\|c.43.1 (0.4739884) b.84.1\|a.9.1\|c.43.1 (0.3930636) -> a.9.1\|c.43.1 (0.6820809): b.84.1&\|a.9.1\|c.43.1\|c.3.1\|d.87.1 (0.6763006) c.26.1\|a.27.1 (0.2658960) -> d.67.2\|c.26.1 (0.6820809): d.67.2\|c.26.1&\|a.27.1 (0.6763006) | 62 c.3.1\|d.87.1 (0.1560694) -> a.9.1\|c.43.1 (0.6820809): b.84.1&\|a.9.1\|c.43.1\|c.3.1\|d.87.1 (0.6763006) d.110.3\|a.30.2 (0.6763006) -> d.58.29\|c.1.33 (0.6878613): c.23.1\|d.110.3\|a.30.2\|d.110.3&\|d.58.29\|c.1.33 (0.6820809) d.110.3\|a.30.2 (0.6763006) -> c.23.1\|d.110.3 (0.6994219): c.23.1\|d.110.3\|a.30.2\|d.122.1\|c.23.1& (0.6820809) | 280 |
| Overlap(s) | Fusion: 299 g.18.1\|b.23.1\|g.18.1 (0.6647399) -> b.23.1\|g.18.1\|b.23.1\|g.18.1\|b.23.1 (0.6763006): b.23.1\|g.18.1\|b.23.1\|g.18.1\|b.23.1\|g.18.1& (0.6647399) c.3.1\|d.87.1 (0.1560694) -> b.84.1\|a.9.1\|c.43.1\|c.3.1\|d.87.1 (0.6763006): b.84.1&\|a.9.1\|c.43.1\|c.3.1\|d.87.1 (0.6763006) b.84.1\|a.9.1\|c.43.1 (0.3930636) -> b.84.1\|a.9.1\|c.43.1\|c.3.1\|d.87.1 (0.6763006): b.84.1&\|a.9.1\|c.43.1\|c.3.1\|d.87.1 (0.6763006) | Fission: 326 g.3.11\|g.3.9\|g.3.11 (0.6589595) -> g.3.9\|g.3.11 (0.6763006): g.3.11\|g.23.1&\|g.3.11&\|g.3.9\|g.3.11 (0.6763006) b.84.1\|a.9.1\|c.43.1 (0.3930636) -> a.9.1\|c.43.1 (0.6820809): b.84.1&\|a.9.1\|c.43.1 (0.4739884) c.43.1\|e.23.1\|a.28.1\|c.69.1 (0.6763006) -> c.43.1\|e.23.1 (0.6820809): c.43.1&\|e.23.1\|a.28.1\|c.69.1 (0.5953757) | 625 |
| Sum total | 1,064 | 344 | 1,408 |
| 4) Multidomains with repeats in themselves (229 /\ 229 = 229 parents with 110 common) | | | |
|  | Arcs with part of repeat match | Arcs with complete match | Arcs Total |
| Contemporaneous: Fusion | 59 | 103 | 162 |
| Fission | 40 | 53 | 93 |
| Fusion/Fission | 49 | 114 | 163 |
| Overlap(s) | 353 | | |
| Diachronic: Fusion | 166 g.12.1&\|b.23.1& (0.6589595) -> g.12.1&\|b.23.1 (0.6647399): g.12.1&\|b.23.1&\|g.12.1 (0.6647399) b.23.1&\|g.12.1 (0.6589595) -> g.12.1&\|b.23.1 (0.6647399): g.12.1&\|b.23.1&\|g.12.1 (0.6647399) c.43.1&\|e.23.1\|a.28.1\|c.69.1 (0.5953757) -> c.43.1&\|e.23.1\|a.28.1\|c.43.1&\|e.23.1\|a.28.1\|c.43.1 (0.6763006): c.43.1&\|e.23.1\|a.28.1\|c.43.1&\|e.23.1\|a.28.1\|c.43.1&\|e.23.1\|a.28.1\|c.43.1&\|e.23.1\|a.28.1\|c.43.1&\|e.23.1\|a.28.1\|c.43.1&\|e.23.1\|a.28.1\|c.43.1&\|e.23.1\|a.28.1\|c.43.1&\|e.23.1\|a.28.1\|c.69.1 (0.6763006) | 287 c.43.1&\|e.23.1\|a.28.1\|c.69.1 (0.5953757) -> c.43.1&\|e.23.1\|a.28.1\|c.43.1&\|e.23.1\|a.28.1\|c.43.1&\|e.23.1\|a.28.1 (0.6763006): c.43.1&\|e.23.1\|a.28.1\|c.43.1&\|e.23.1\|a.28.1\|c.43.1&\|e.23.1\|a.28.1\|c.43.1&\|e.23.1\|a.28.1\|c.43.1&\|e.23.1\|a.28.1\|c.43.1&\|e.23.1\|a.28.1\|c.43.1&\|e.23.1\|a.28.1\|c.43.1&\|e.23.1\|a.28.1\|c.69.1 (0.6763006) c.43.1&\|e.23.1\|a.28.1\|c.69.1 (0.5953757) -> e.23.1\|a.28.1\|c.43.1&\|e.23.1 (0.6763006): c.43.1&\|e.23.1\|a.28.1\|c.43.1&\|e.23.1\|a.28.1\|c.43.1&\|e.23.1\|a.28.1\|c.43.1&\|e.23.1\|a.28.1\|c.43.1&\|e.23.1\|a.28.1\|c.43.1&\|e.23.1\|a.28.1\|c.43.1&\|e.23.1\|a.28.1\|c.43.1&\|e.23.1\|a.28.1\|c.69.1 (0.6763006) c.43.1&\|e.23.1\|a.28.1\|c.69.1 (0.5953757) -> e.23.1\|a.28.1\|c.43.1&\|e.23.1\|a.28.1 (0.6763006): c.43.1&\|e.23.1\|a.28.1\|c.43.1&\|e.23.1\|a.28.1\|c.43.1&\|e.23.1\|a.28.1\|c.43.1&\|e.23.1\|a.28.1\|c.43.1&\|e.23.1\|a.28.1\|c.43.1&\|e.23.1\|a.28.1\|c.43.1&\|e.23.1\|a.28.1\|c.43.1&\|e.23.1\|a.28.1\|c.69.1 (0.6763006) | 453 |
| Fission | 348 b.1.2&\|b.1.1\|b.1.2 (0.6763006) -> b.1.2&\|b.1.1\|b.1.2& (0.6820809): b.1.1\|b.1.2&\|b.1.1\|b.1.2&\|b.1.1\|b.1.2& (0.6589595) b.1.1\|b.1.2&\|b.1.1\|b.1.2 (0.6763006) -> b.1.2&\|b.1.1\|b.1.2& (0.6820809): b.1.1\|b.1.2&\|b.1.1\|b.1.2&\|b.1.1\|b.1.2& (0.6589595) b.1.2&\|b.1.1\|b.1.2&\|b.1.1\|b.1.2 (0.6763006) -> b.1.2&\|b.1.1\|b.1.2& (0.6820809): b.1.1\|b.1.2&\|b.1.1\|b.1.2&\|b.1.1\|b.1.2& (0.6589595) | 824 b.1.2&\|b.1.1\|b.1.2&\|b.1.1 (0.6763006) -> b.1.2&\|b.1.1\|b.1.2& (0.6820809): b.1.1\|b.1.2&\|b.1.1\|b.1.2&\|b.1.1\|b.1.2& (0.6589595) b.1.2&\|b.1.1\|b.1.2&\|b.1.1\|b.1.2& (0.6763006) -> b.1.2&\|b.1.1\|b.1.2& (0.6820809): b.1.1\|b.1.2&\|b.1.1\|b.1.2&\|b.1.1\|b.1.2& (0.6589595) e.23.1\|a.28.1\|c.43.1&\|e.23.1 (0.6763006) -> e.23.1\|a.28.1\|c.43.1& (0.6820809): c.43.1&\|e.23.1\|a.28.1\|c.43.1&\|e.23.1\|a.28.1\|c.43.1&\|e.23.1\|a.28.1 (0.6763006) | 1,172 |
| Fusion/Fission | 72 c.43.1&\|e.23.1\|a.28.1\|c.69.1 (0.5953757) -> c.43.1\|e.23.1\|a.28.1\|c.43.1& (0.6820809): c.43.1&\|e.23.1\|a.28.1\|c.43.1&\|e.23.1\|a.28.1\|c.43.1&\|e.23.1\|a.28.1\|c.43.1&\|e.23.1\|a.28.1\|c.43.1&\|e.23.1\|a.28.1\|c.43.1&\|e.23.1\|a.28.1\|c.43.1&\|e.23.1\|a.28.1\|c.43.1&\|e.23.1\|a.28.1\|c.69.1 (0.6763006) c.43.1&\|e.23.1\|a.28.1\|c.69.1 (0.5953757) -> c.43.1\|e.23.1\|a.28.1\|c.43.1& (0.6820809): c.43.1&\|e.23.1\|a.28.1\|c.43.1&\|e.23.1\|a.28.1\|c.43.1&\|e.23.1\|a.28.1\|c.43.1&\|e.23.1\|a.28.1\|c.43.1&\|e.23.1\|a.28.1\|c.43.1&\|e.23.1\|a.28.1\|c.43.1&\|e.23.1\|a.28.1\|c.43.1&\|e.23.1\|a.28.1\|c.43.1&\|e.23.1\|a.28.1\|c.69.1 (0.6763006) c.43.1&\|e.23.1\|a.28.1\|c.69.1 (0.5953757) -> c.43.1\|e.23.1\|a.28.1\|c.43.1& (0.6820809): c.43.1&\|e.23.1\|a.28.1\|c.43.1&\|e.23.1\|a.28.1\|c.43.1&\|e.23.1\|a.28.1\|c.43.1&\|e.23.1\|a.28.1\|c.43.1&\|e.23.1\|a.28.1\|c.69.1 (0.6763006) | 285 c.43.1&\|e.23.1\|a.28.1\|c.69.1 (0.5953757) -> c.43.1&\|e.23.1\|a.28.1\|c.43.1&\|e.23.1\|a.28.1\|c.69.1 (0.6820809): c.43.1&\|e.23.1\|a.28.1\|c.43.1&\|e.23.1\|a.28.1\|c.43.1&\|e.23.1\|a.28.1\|c.43.1&\|e.23.1\|a.28.1\|c.43.1&\|e.23.1\|a.28.1\|c.43.1&\|e.23.1\|a.28.1\|c.43.1&\|e.23.1\|a.28.1\|c.43.1&\|e.23.1\|a.28.1\|c.69.1 (0.6763006) c.43.1&\|e.23.1\|a.28.1\|c.69.1 (0.5953757) -> e.23.1\|a.28.1\|c.43.1& (0.6820809): c.43.1&\|e.23.1\|a.28.1\|c.43.1&\|e.23.1\|a.28.1\|c.43.1&\|e.23.1\|a.28.1\|c.43.1&\|e.23.1\|a.28.1\|c.43.1&\|e.23.1\|a.28.1\|c.43.1&\|e.23.1\|a.28.1\|c.43.1&\|e.23.1\|a.28.1\|c.43.1&\|e.23.1\|a.28.1\|c.69.1 (0.6763006) c.43.1&\|e.23.1\|a.28.1\|c.69.1 (0.5953757) -> a.28.1\|c.43.1&\|e.23.1\|a.28.1\|c.43.1&\|e.23.1\|a.28.1 (0.6820809): c.43.1&\|e.23.1\|a.28.1\|c.43.1&\|e.23.1\|a.28.1\|c.43.1&\|e.23.1\|a.28.1\|c.43.1&\|e.23.1\|a.28.1\|c.43.1&\|e.23.1\|a.28.1\|c.43.1&\|e.23.1\|a.28.1\|c.43.1&\|e.23.1\|a.28.1\|c.43.1&\|e.23.1\|a.28.1\|c.69.1 (0.6763006) | 357 |
| Overlap(s) | Fusion: 593 c.43.1&\|e.23.1\|a.28.1\|c.69.1 (0.5953757) -> c.43.1&\|e.23.1\|a.28.1\|c.43.1&\|e.23.1\|a.28.1\|c.43.1&\|e.23.1\|a.28.1\|c.43.1&\|e.23.1\|a.28.1\|c.43.1&\|e.23.1\|a.28.1\|c.69.1 (0.6763006): c.43.1&\|e.23.1\|a.28.1\|c.43.1&\|e.23.1\|a.28.1\|c.43.1&\|e.23.1\|a.28.1\|c.43.1&\|e.23.1\|a.28.1\|c.43.1&\|e.23.1\|a.28.1\|c.43.1&\|e.23.1\|a.28.1\|c.43.1&\|e.23.1\|a.28.1\|c.43.1&\|e.23.1\|a.28.1\|c.69.1 (0.6763006) c.43.1&\|e.23.1\|a.28.1\|c.69.1 (0.5953757) -> c.43.1&\|e.23.1\|a.28.1\|c.43.1&\|e.23.1\|a.28.1\|c.43.1&\|e.23.1\|a.28.1\|c.43.1&\|e.23.1\|a.28.1\|c.43.1&\|e.23.1\|a.28.1\|c.43.1&\|e.23.1\|a.28.1\|c.43.1&\|e.23.1\|a.28.1\|c.43.1&\|e.23.1\|a.28.1\|c.69.1 (0.6763006): c.43.1&\|e.23.1\|a.28.1\|c.43.1&\|e.23.1\|a.28.1\|c.43.1&\|e.23.1\|a.28.1\|c.43.1&\|e.23.1\|a.28.1\|c.43.1&\|e.23.1\|a.28.1\|c.43.1&\|e.23.1\|a.28.1\|c.43.1&\|e.23.1\|a.28.1\|c.43.1&\|e.23.1\|a.28.1\|c.43.1&\|e.23.1\|a.28.1\|c.69.1 (0.6763006) c.43.1&\|e.23.1\|a.28.1\|c.69.1 (0.5953757) -> c.43.1&\|e.23.1\|a.28.1\|c.43.1&\|e.23.1\|a.28.1\|c.43.1&\|e.23.1\|a.28.1\|c.43.1&\|e.23.1\|a.28.1\|c.43.1&\|e.23.1\|a.28.1\|c.69.1 (0.6763006): c.43.1&\|e.23.1\|a.28.1\|c.43.1&\|e.23.1\|a.28.1\|c.43.1&\|e.23.1\|a.28.1\|c.43.1&\|e.23.1\|a.28.1\|c.43.1&\|e.23.1\|a.28.1\|c.43.1&\|e.23.1\|a.28.1\|c.43.1&\|e.23.1\|a.28.1\|c.43.1&\|e.23.1\|a.28.1\|c.43.1&\|e.23.1\|a.28.1\|c.69.1 (0.6763006) | Fission: 716 g.12.1&\|b.23.1& (0.6589595) -> g.12.1&\|b.23.1 (0.6647399): g.12.1&\|b.23.1&\|g.12.1 (0.6647399) c.43.1&\|e.23.1\|a.28.1\|c.69.1 (0.5953757) -> c.43.1&\|e.23.1 (0.6763006): c.43.1&\|e.23.1\|a.28.1\|c.43.1&\|e.23.1\|a.28.1\|c.43.1&\|e.23.1\|a.28.1\|c.43.1&\|e.23.1\|a.28.1\|c.43.1&\|e.23.1\|a.28.1\|c.43.1&\|e.23.1\|a.28.1\|c.43.1&\|e.23.1\|a.28.1\|c.43.1&\|e.23.1\|a.28.1\|c.69.1 (0.6763006) c.43.1&\|e.23.1\|a.28.1\|c.69.1 (0.5953757) -> c.43.1&\|e.23.1 (0.6763006): c.43.1&\|e.23.1\|a.28.1\|c.43.1&\|e.23.1\|a.28.1\|c.43.1&\|e.23.1\|a.28.1\|c.43.1&\|e.23.1\|a.28.1\|c.43.1&\|e.23.1\|a.28.1\|c.43.1&\|e.23.1\|a.28.1\|c.43.1&\|e.23.1\|a.28.1\|c.43.1&\|e.23.1\|a.28.1\|c.43.1&\|e.23.1\|a.28.1\|c.69.1 (0.6763006) | 1,309 |
| Sum total | 734 | 1,666 | 2,400 |
| Paired set comparisons | | | |
| 5) Domains and domain repeats in multidomains with repeats (777 /\ 720 = 720 parents with 720 common) | | | |
|  | Arcs with part of repeat match | Arcs with complete match | Arcs Total |
| Contemporaneous: Fusion | 0 | 18 | 18 |
| Fission | 15 | 40 | 55 |
| Fusion/Fission | 0 | 2 | 2 |
| Overlap(s) | 16 | | |
| Diachronic: Fusion | 130 c.37.1 (0.0000000) -> c.37.1& (0.1907514): a.174.1\|c.37.1& (0.3121387) c.55.1& (0.0462428) -> c.55.1 (0.1907514): c.55.1&\|a.211.1 (0.4450867) c.37.1 (0.0000000) -> c.37.1& (0.1907514): c.37.1&\|c.52.1 (0.4682081) | 177 c.55.1& (0.0462428) -> a.211.1 (0.2543353): c.55.1&\|a.211.1 (0.4450867) c.37.1& (0.1907514) -> c.52.1 (0.3699422): c.37.1&\|c.52.1 (0.4682081) c.2.1 (0.0057803) -> d.58.18& (0.3294798): c.73.1\|d.58.18&\|c.2.1\|d.81.1 (0.5202312) | 307 |
| Fission | 484 a.4.1& (0.6011561) -> a.4.1 (0.6647399): a.4.1&\|d.60.1 (0.5491329) a.4.1& (0.6011561) -> a.4.1 (0.6647399): b.82.4\|a.4.1& (0.5549133) b.1.5& (0.6820809) -> b.1.5 (0.6878613): b.12.1\|b.1.18\|d.3.1\|b.1.5& (0.6647399) | 558 d.58.18& (0.3294798) -> c.73.1 (0.3641618): c.73.1\|d.58.18& (0.3179191) f.7.1& (0.6763006) -> a.118.4 (0.6820809): f.7.1\|a.118.4\|f.7.1& (0.6763006) a.4.1& (0.6011561) -> b.82.4 (0.6878613): b.82.4\|a.4.1& (0.5549133) | 1,042 |
| Fusion/Fission | 176 b.40.4 (0.0520231) -> b.40.4& (0.6358382): b.34.5\|b.40.4& (0.3005780) d.38.1 (0.1271676) -> d.106.1& (0.6763006): c.2.1\|d.38.1&\|d.106.1& (0.6647399) d.38.1 (0.1271676) -> d.38.1& (0.6820809): c.2.1\|d.38.1&\|d.106.1& (0.6647399) | 340 b.34.5 (0.1849711) -> b.40.4& (0.6358382): b.34.5\|b.40.4& (0.3005780) c.37.1& (0.1907514) -> d.315.1 (0.6763006): c.37.1&\|b.34.18\|c.37.1&\|d.315.1 (0.4913295) c.37.1 (0.0000000) -> h.1.26& (0.6763006): c.37.1\|h.1.26& (0.6647399) | 516 |
| Overlap(s) | Fusion: 322 c.37.1 (0.0000000) -> c.37.1& (0.1907514): a.174.1\|c.37.1& (0.3121387) c.37.1 (0.0000000) -> c.37.1& (0.1907514): c.37.1&\|c.52.1 (0.4682081) c.37.1 (0.0000000) -> c.37.1& (0.1907514): c.37.1&\|b.34.18\|c.37.1&\|d.315.1 (0.4913295) | Fission: 433 c.55.1& (0.0462428) -> c.55.1 (0.1907514): c.55.1&\|a.211.1 (0.4450867) a.4.1& (0.6011561) -> a.4.1 (0.6647399): a.4.1&\|d.60.1 (0.5491329) a.4.1& (0.6011561) -> a.4.1 (0.6647399): b.82.4\|a.4.1& (0.5549133) | 755 |
| Sum total | 805 | 1,135 | 1,940 |
| 6a) Domains and multidomains with no repeats in the latter (3,483 /\ 908 = 908 parents with 908 common) | | | |
|  | Arcs with part of repeat match | Arcs with complete match | Arcs Total |
| Contemporaneous: Fusion | 0 | 64 | 64 |
| Fission | 0 | 103 | 103 |
| Fusion/Fission | 0 | 13 | 13 |
| Overlap(s) | 127 | | |
| Diachronic: Fusion | 0 | 1,912 c.2.1 (0.0057803) -> c.2.1\|d.81.1 (0.0867052): c.2.1\|d.81.1\|a.69.3 (0.3294798) b.40.4 (0.0520231) -> d.14.1\|d.101.1 (0.2716763): d.14.1\|d.101.1\|a.4.9\|d.14.1\|d.101.1\|d.51.1\|b.40.4 (0.3352601) d.14.1 (0.1791908) -> d.14.1\|d.101.1 (0.2716763): d.14.1\|d.101.1\|a.4.9\|d.14.1\|d.101.1\|d.51.1\|b.40.4 (0.3352601) | 1,912 |
| Fission | 0 | 2,728 c.30.1\|d.142.1 (0.3179191) -> c.24.1 (0.5491329): c.30.1\|d.142.1\|a.92.1\|c.30.1\|d.142.1\|c.24.1 (0.3121387) c.23.1 (0.4046243) -> c.37.1\|a.4.1 (0.5780347): c.23.1\|c.37.1\|a.4.1 (0.3699422) c.37.1\|a.4.1 (0.5780347) -> a.4.1 (0.6647399): c.23.1\|c.37.1\|a.4.1 (0.3699422) | 2,728 |
| Fusion/Fission | 0 | 2,855 c.26.1 (0.1213873) -> c.26.1\|a.27.1 (0.2658960): d.67.2\|c.26.1\|a.27.1 (0.2312139) d.104.1\|c.51.1 (0.2312139) -> d.104.1 (0.3757226): d.15.10\|d.67.1\|d.104.1\|c.51.1 (0.3468208) c.37.1 (0.0000000) -> c.37.1\|a.4.1 (0.5780347): c.23.1\|c.37.1\|a.4.1 (0.3699422) | 2,855 |
| Overlap(s) | Fusion: 1,825 c.26.1 (0.1213873) -> c.26.1\|a.27.1 (0.2658960): d.67.2\|c.26.1\|a.27.1 (0.2312139) c.2.1 (0.0057803) -> c.2.1\|d.81.1 (0.0867052): c.2.1\|d.81.1\|a.69.3 (0.3294798) d.14.1 (0.1791908) -> d.14.1\|d.101.1 (0.2716763): d.14.1\|d.101.1\|a.4.9\|d.14.1\|d.101.1\|d.51.1\|b.40.4 (0.3352601) | Fission: 2,703 d.104.1\|c.51.1 (0.2312139) -> d.104.1 (0.3757226): d.15.10\|d.67.1\|d.104.1\|c.51.1 (0.3468208) c.37.1\|a.4.1 (0.5780347) -> a.4.1 (0.6647399): c.23.1\|c.37.1\|a.4.1 (0.3699422) g.39.1\|c.37.1 (0.3121387) -> g.39.1 (0.3468208): b.113.1\|a.156.1\|g.39.1\|c.37.1 (0.6705202) | 4,528 |
| Sum total | 0 | 7,675 | 7,675 |
| 6b) Domains and multidomains with no repeats in multidomains with repeats (777 /\ 535 = 535 parents with 535 common) | | | |
|  | Arcs with part of repeat match | Arcs with complete match | Arcs Total |
| Contemporaneous: Fusion | 7 | 3 | 10 |
| Fission | 43 | 16 | 59 |
| Fusion/Fission | 6 | 0 | 6 |
| Overlap(s) | 50 | | |
| Diachronic: Fusion | 388 c.2.1 (0.0057803) -> c.2.1\|a.100.1 (0.0693642): c.2.1\|a.100.1& (0.5086705) c.2.1 (0.0057803) -> c.2.1\|a.100.1 (0.0693642): c.14.1\|c.2.1\|a.100.1& (0.6416185) c.2.1\|a.100.1 (0.0693642) -> c.14.1 (0.1387283): c.14.1\|c.2.1\|a.100.1& (0.6416185) | 148 c.2.1 (0.0057803) -> c.2.1\|d.81.1 (0.0867052): c.73.1\|d.58.18&\|c.2.1\|d.81.1 (0.5202312) c.2.1\|d.81.1 (0.0867052) -> c.73.1 (0.3641618): c.73.1\|d.58.18&\|c.2.1\|d.81.1 (0.5202312) c.68.1 (0.0693642) -> c.68.1\|b.82.1 (0.6127167): c.84.1&\|d.129.2\|c.68.1\|b.82.1 (0.6763006) | 536 |
| Fission | 1,124 d.315.1 (0.6763006) -> c.37.1\|d.315.1 (0.6820809): c.37.1&\|b.34.18\|c.37.1&\|d.315.1 (0.4913295) d.110.3\|d.110.2 (0.6763006) -> a.30.2 (0.6820809): d.110.3&\|d.110.2\|d.110.3\|a.30.2\|d.122.1 (0.6705202) d.110.3\|d.110.2 (0.6763006) -> a.30.2 (0.6820809): d.110.3\|d.110.2&\|a.30.2\|d.122.1\|c.23.1 (0.6763006) | 537 d.110.2\|d.110.3 (0.6763006) -> a.30.2 (0.6820809): d.110.3&\|d.110.2\|d.110.3\|d.110.2\|a.30.2\|d.122.1\|c.23.1 (0.6705202) d.110.3\|d.110.2 (0.6763006) -> a.30.2 (0.6820809): d.110.3&\|d.110.2\|d.110.3\|d.110.2\|a.30.2\|d.122.1\|c.23.1 (0.6705202) d.110.3\|d.110.2\|a.30.2\|d.122.1 (0.6763006) -> a.30.2 (0.6820809): d.110.3&\|d.110.2\|d.110.3\|d.110.2\|a.30.2\|d.122.1\|c.23.1 (0.6705202) | 1,661 |
| Fusion/Fission | 854 c.37.1 (0.0000000) -> c.37.1\|c.52.1 (0.6705202): c.37.1&\|c.52.1 (0.4682081) c.52.1 (0.3699422) -> c.37.1\|c.52.1 (0.6705202): c.37.1&\|c.52.1 (0.4682081) c.55.1 (0.1907514) -> c.55.1\|a.211.1 (0.6763006): c.55.1&\|a.211.1 (0.4450867) | 367 c.89.1 (0.3410405) -> g.3.11\|d.169.1 (0.6763006): d.170.1\|g.3.11\|d.169.1\|g.3.11&\|c.89.1 (0.6647399) c.23.1 (0.4046243) -> d.110.2\|d.110.3 (0.6763006): d.110.3&\|d.110.2\|d.110.3\|d.110.2\|a.30.2\|d.122.1\|c.23.1 (0.6705202) c.23.1 (0.4046243) -> d.110.3\|d.110.2 (0.6763006): d.110.3&\|d.110.2\|d.110.3\|d.110.2\|a.30.2\|d.122.1\|c.23.1 (0.6705202) | 1,221 |
| Overlap(s) | Fusion: 938 c.2.1 (0.0057803) -> c.2.1\|a.100.1 (0.0693642): c.2.1\|a.100.1& (0.5086705) c.2.1 (0.0057803) -> c.2.1\|d.81.1 (0.0867052): c.73.1\|d.58.18&\|c.2.1\|d.81.1 (0.5202312) c.2.1 (0.0057803) -> c.2.1\|a.100.1 (0.0693642): c.14.1\|c.2.1\|a.100.1& (0.6416185) | Fission: 1,462 d.58.42\|b.34.5 (0.3352601) -> d.58.42 (0.6763006): d.58.42&\|b.34.5 (0.6763006) c.3.1\|d.87.1 (0.1560694) -> c.3.1 (0.1849711): b.84.1&\|a.9.1\|c.43.1\|c.3.1\|d.87.1 (0.6763006) c.142.1\|d.15.13\|a.29.12 (0.6416185) -> c.142.1 (0.6763006): c.47.1&\|c.142.1\|d.15.13\|a.29.12 (0.6763006) | 2,400 |
| Sum total | 2,422 | 1,071 | 3,493 |
| 7) Domains and multidomains with repeats in the latter (777 /\ 229 = 229 parents with 229 common) | | | |
|  | Arcs with part of repeat match | Arcs with complete match | Arcs Total |
| Contemporaneous: Fusion | 4 | 3 | 7 |
| Fission | 13 | 10 | 23 |
| Fusion/Fission | 0 | 0 | 0 |
| Overlap(s) | 22 | | |
| Diachronic: Fusion | 73 e.23.1 (0.1445087) -> c.43.1&\|e.23.1\|a.28.1\|c.43.1&\|e.23.1\|a.28.1\|c.43.1 (0.6763006): c.43.1&\|e.23.1\|a.28.1\|c.43.1&\|e.23.1\|a.28.1\|c.43.1&\|e.23.1\|a.28.1 (0.6763006) a.28.1 (0.3526012) -> c.43.1&\|e.23.1\|a.28.1\|c.43.1&\|e.23.1\|a.28.1\|c.43.1 (0.6763006): c.43.1&\|e.23.1\|a.28.1\|c.43.1&\|e.23.1\|a.28.1\|c.43.1&\|e.23.1\|a.28.1 (0.6763006) c.69.1 (0.0346821) -> c.43.1&\|e.23.1\|a.28.1\|c.43.1&\|e.23.1\|a.28.1\|c.43.1 (0.6763006): c.43.1&\|e.23.1\|a.28.1\|c.43.1&\|e.23.1\|a.28.1\|c.43.1&\|e.23.1\|a.28.1\|c.43.1&\|e.23.1\|a.28.1\|c.43.1&\|e.23.1\|a.28.1\|c.43.1&\|e.23.1\|a.28.1\|c.43.1&\|e.23.1\|a.28.1\|c.43.1&\|e.23.1\|a.28.1\|c.69.1 (0.6763006) | 403 c.2.1 (0.0057803) -> c.73.1\|d.58.18& (0.3179191): c.73.1\|d.58.18&\|c.2.1\|d.81.1 (0.5202312) c.73.1\|d.58.18& (0.3179191) -> c.73.1 (0.3641618): c.73.1\|d.58.18&\|c.2.1\|d.81.1 (0.5202312) c.2.1 (0.0057803) -> c.2.1\|a.100.1& (0.5086705): c.14.1\|c.2.1\|a.100.1& (0.6416185) | 476 |
| Fission | 456 d.110.2\|d.110.3&\|a.30.2\|d.122.1\|c.23.1 (0.6763006) -> a.30.2 (0.6820809): c.23.1\|d.110.2\|d.110.3&\|a.30.2\|d.122.1\|c.23.1&\|a.24.10 (0.6763006) d.110.3\|a.30.2\|d.122.1\|c.23.1&\|a.24.10 (0.6763006) -> a.30.2 (0.6820809): c.23.1\|d.110.2\|d.110.3&\|a.30.2\|d.122.1\|c.23.1&\|a.24.10 (0.6763006) g.23.1 (0.6763006) -> g.23.1&\|g.3.11 (0.6878613): g.3.11\|g.23.1&\|g.3.11&\|g.3.9\|g.3.11 (0.6763006) | 437 d.110.2\|d.110.3&\|a.30.2\|d.122.1\|c.23.1 (0.6763006) -> a.30.2 (0.6820809): d.110.3\|d.110.2\|d.110.3&\|a.30.2\|d.122.1\|c.23.1 (0.6763006) d.110.3&\|d.110.2\|a.30.2\|d.122.1 (0.6763006) -> a.30.2 (0.6820809): c.23.1\|d.110.3&\|d.110.2\|a.30.2\|d.122.1 (0.6763006) c.23.1\|d.110.3&\|d.110.2 (0.6763006) -> a.30.2 (0.6820809): c.23.1\|d.110.3&\|d.110.2\|a.30.2\|d.122.1 (0.6763006) | 893 |
| Fusion/Fission | 267 c.37.1 (0.0000000) -> c.37.1&\|b.34.18 (0.6763006): c.37.1&\|b.34.18\|c.37.1&\|d.315.1 (0.4913295) d.38.1 (0.1271676) -> c.2.1\|d.38.1& (0.6763006): c.2.1\|d.38.1&\|d.106.1& (0.6647399) e.23.1 (0.1445087) -> c.43.1\|e.23.1\|a.28.1\|c.43.1& (0.6820809): c.43.1&\|e.23.1\|a.28.1\|c.43.1&\|e.23.1\|a.28.1\|c.43.1&\|e.23.1\|a.28.1 (0.6763006) | 533 c.69.1 (0.0346821) -> c.43.1&\|e.23.1 (0.6763006): c.43.1&\|e.23.1\|a.28.1\|c.69.1 (0.5953757) e.23.1 (0.1445087) -> c.43.1&\|e.23.1 (0.6763006): c.43.1&\|e.23.1\|a.28.1\|c.69.1 (0.5953757) a.28.1 (0.3526012) -> c.43.1&\|e.23.1 (0.6763006): c.43.1&\|e.23.1\|a.28.1\|c.69.1 (0.5953757) | 800 |
| Overlap(s) | Fusion: 690 c.2.1 (0.0057803) -> c.2.1\|a.100.1& (0.5086705): c.14.1\|c.2.1\|a.100.1& (0.6416185) c.37.1 (0.0000000) -> c.37.1&\|b.34.18 (0.6763006): c.37.1&\|b.34.18\|c.37.1&\|d.315.1 (0.4913295) e.23.1 (0.1445087) -> c.43.1&\|e.23.1 (0.6763006): c.43.1&\|e.23.1\|a.28.1\|c.69.1 (0.5953757) | Fission: 1,019 c.73.1\|d.58.18& (0.3179191) -> c.73.1 (0.3641618): c.73.1\|d.58.18&\|c.2.1\|d.81.1 (0.5202312) c.73.1\|d.58.18& (0.3179191) -> c.73.1 (0.3641618): d.58.4\|c.73.1\|d.58.18& (0.6763006) c.36.1&\|c.48.1 (0.1849711) -> c.36.1 (0.2601156): c.36.1&\|c.48.1\|c.1.10 (0.6763006) | 1,709 |
| Sum total | 813 | 1,386 | 2,199 |
| 8) Domain repeats and multidomains with no repeats in multidomains with repeats (720 /\ 535 = 486 parents with 486 common) | | | |
|  | Arcs with part of repeat match | Arcs with complete match | Arcs Total |
| Contemporaneous: Fusion | 2 | 1 | 3 |
| Fission | 7 | 1 | 8 |
| Fusion/Fission | 3 | 0 | 3 |
| Diachronic: Fusion | 113 a.4.1& (0.6011561) -> a.4.1\|d.32.1 (0.6763006): a.4.1&\|d.32.1 (0.6763006) b.40.4& (0.6358382) -> a.170.1\|b.40.4\|a.171.1\|b.40.4 (0.6763006): a.170.1\|b.40.4\|a.171.1\|b.40.4& (0.6763006) d.110.3\|d.110.2 (0.6763006) -> d.110.2& (0.6820809): d.110.3\|d.110.2&\|d.58.29 (0.6820809) | 37 c.2.1\|d.81.1 (0.0867052) -> d.58.18& (0.3294798): c.73.1\|d.58.18&\|c.2.1\|d.81.1 (0.5202312) c.47.1\|a.45.1 (0.3179191) -> a.93.1& (0.5202312): c.47.1\|a.45.1\|a.93.1& (0.6763006) b.40.4& (0.6358382) -> a.170.1\|b.40.4 (0.6763006): a.170.1\|b.40.4\|a.171.1\|b.40.4& (0.6763006) | 150 |
| Fission | 395 a.177.1\|a.4.13 (0.3988439) -> a.4.13& (0.6763006): a.177.1\|a.4.13& (0.3757226) c.2.1\|d.38.1 (0.6763006) -> d.38.1& (0.6820809): c.2.1\|d.38.1&\|d.106.1& (0.6647399) d.110.3\|d.110.2 (0.6763006) -> d.110.3& (0.6820809): d.110.3&\|d.110.2\|d.110.3\|a.30.2\|d.122.1 (0.6705202) | 220 d.110.2\|d.110.3 (0.6763006) -> d.110.3& (0.6820809): d.110.3&\|d.110.2\|d.110.3\|d.110.2\|a.30.2\|d.122.1\|c.23.1 (0.6705202) d.110.3\|d.110.2 (0.6763006) -> d.110.3& (0.6820809): d.110.3&\|d.110.2\|d.110.3\|d.110.2\|a.30.2\|d.122.1\|c.23.1 (0.6705202) d.110.3\|d.110.2\|a.30.2\|d.122.1 (0.6763006) -> d.110.3& (0.6820809): d.110.3&\|d.110.2\|d.110.3\|d.110.2\|a.30.2\|d.122.1\|c.23.1 (0.6705202) | 615 |
| Fusion/Fission | 228 c.37.1& (0.1907514) -> c.37.1\|c.52.1 (0.6705202): c.37.1&\|c.52.1 (0.4682081) c.55.1& (0.0462428) -> c.55.1\|a.211.1 (0.6763006): c.55.1&\|a.211.1 (0.4450867) c.37.1& (0.1907514) -> c.37.1\|d.315.1 (0.6820809): c.37.1&\|b.34.18\|c.37.1&\|d.315.1 (0.4913295) | 93 d.37.1& (0.3641618) -> d.58.29\|c.1.33 (0.6878613): d.37.1&\|d.110.3&\|d.58.29\|c.1.33 (0.6763006) b.43.4\|c.25.1 (0.3352601) -> c.23.5& (0.6878613): c.23.5&\|b.43.4\|c.25.1 (0.6820809) c.68.1\|b.82.1 (0.6127167) -> c.84.1& (0.6936416): c.84.1&\|d.129.2\|c.68.1\|b.82.1 (0.6763006) | 321 |
| Sum total | 748 | 352 | 1,100 |
| 9) Domain repeats and multidomains with repeats in the latter (720 /\ 229 = 220 parents with 220 common) | | | |
|  | Arcs with part of repeat match | Arcs with complete match | Arcs Total |
| Contemporaneous: Fusion | 0 | 3 | 3 |
| Fission | 1 | 9 | 10 |
| Fusion/Fission | 0 | 2 | 2 |
| Overlap(s) | 10 | | |
| Diachronic: Fusion | 4 c.37.1& (0.1907514) -> b.34.13\|c.37.1& (0.6763006): b.34.13&\|c.37.1& (0.6878613) b.14.1& (0.6820809) -> a.7.14\|d.3.1\|b.14.1& (0.7052023): a.7.14&\|d.3.1\|b.14.1& (0.7167630) a.7.1&\|a.39.1 (0.7052023) -> a.7.1& (0.7283237): a.40.1\|a.7.1&\|a.39.1& (0.7514451) | 36 c.73.1\|d.58.18& (0.3179191) -> d.58.18& (0.3294798): c.73.1\|d.58.18&\|c.2.1\|d.81.1 (0.5202312) c.73.1\|d.58.18& (0.3179191) -> d.58.18& (0.3294798): d.58.4\|c.73.1\|d.58.18& (0.6763006) d.110.3&\|d.110.2\|a.30.2\|d.122.1 (0.6763006) -> d.110.3& (0.6820809): c.23.1\|d.110.3&\|d.110.2\|d.110.3&\|d.110.2\|a.30.2\|d.122.1 (0.6820809) | 40 |
| Fission | 110 d.110.2\|d.110.3&\|a.30.2\|d.122.1\|c.23.1 (0.6763006) -> d.110.3& (0.6820809): c.23.1\|d.110.2\|d.110.3&\|a.30.2\|d.122.1\|c.23.1&\|a.24.10 (0.6763006) d.110.3\|a.30.2\|d.122.1\|c.23.1&\|a.24.10 (0.6763006) -> d.110.3& (0.6820809): c.23.1\|d.110.2\|d.110.3&\|a.30.2\|d.122.1\|c.23.1&\|a.24.10 (0.6763006) d.106.1& (0.6763006) -> d.38.1&\|d.106.1 (0.6878613): c.2.1\|d.38.1&\|d.106.1& (0.6647399) | 365 c.2.1\|d.38.1& (0.6763006) -> d.38.1& (0.6820809): c.2.1\|d.38.1&\|d.106.1& (0.6647399) d.110.2\|d.110.3&\|a.30.2\|d.122.1\|c.23.1 (0.6763006) -> d.110.3& (0.6820809): d.110.3\|d.110.2\|d.110.3&\|a.30.2\|d.122.1\|c.23.1 (0.6763006) d.110.3&\|d.110.2\|a.30.2\|d.122.1 (0.6763006) -> d.110.3& (0.6820809): c.23.1\|d.110.3&\|d.110.2\|a.30.2\|d.122.1 (0.6763006) | 475 |
| Fusion/Fission | 60 c.37.1& (0.1907514) -> c.37.1\|b.34.18\|c.37.1&\|d.315.1 (0.6936416): c.37.1&\|b.34.18\|c.37.1&\|d.315.1 (0.4913295) d.110.3& (0.6820809) -> c.23.1\|d.110.3& (0.6994219): c.23.1&\|d.110.3&\|a.30.2\|d.122.1 (0.6878613) d.110.3& (0.6820809) -> d.110.3\|a.30.2\|d.122.1\|c.23.1& (0.6994219): c.23.1\|d.110.3&\|a.30.2\|d.122.1\|c.23.1& (0.6878613) | 187 c.37.1& (0.1907514) -> c.37.1&\|b.34.18 (0.6763006): c.37.1&\|b.34.18\|c.37.1&\|d.315.1 (0.4913295) c.2.1\|a.100.1& (0.5086705) -> a.100.1& (0.6878613): c.14.1\|c.2.1\|a.100.1& (0.6416185) c.37.1& (0.1907514) -> b.34.18\|c.37.1&\|d.315.1 (0.6936416): c.37.1&\|b.34.18\|c.37.1&\|d.315.1 (0.4913295) | 247 |
| Overlap(s) | Fusion: 115 c.37.1& (0.1907514) -> c.37.1&\|b.34.18 (0.6763006): c.37.1&\|b.34.18\|c.37.1&\|d.315.1 (0.4913295) d.38.1& (0.6820809) -> d.38.1&\|d.106.1 (0.6878613): c.2.1\|d.38.1&\|d.106.1& (0.6647399) d.110.3& (0.6820809) -> c.23.1\|d.110.3\|d.110.2\|d.110.3& (0.6878613): c.23.1\|d.110.3\|d.110.2\|d.110.3&\|a.30.2\|d.122.1 (0.6763006) | Fission: 553 c.73.1\|d.58.18& (0.3179191) -> d.58.18& (0.3294798): c.73.1\|d.58.18&\|c.2.1\|d.81.1 (0.5202312) c.73.1\|d.58.18& (0.3179191) -> d.58.18& (0.3294798): d.58.4\|c.73.1\|d.58.18& (0.6763006) c.2.1\|d.38.1& (0.6763006) -> d.38.1& (0.6820809): c.2.1\|d.38.1&\|d.106.1& (0.6647399) | 668 |
| Sum total | 175 | 602 | 777 |
| 10) Multidomains with no repeats and multidomains with repeats in the latter (535 /\ 229 = 215 parents with 215 common) | | | |
|  | Arcs with part of repeat match | Arcs with complete match | Arcs Total |
| Contemporaneous: Fusion | 75 | 25 | 100 |
| Fission | 93 | 55 | 148 |
| Fusion/Fission | 52 | 10 | 62 |
| Overlap(s) | 160 | | |
| Diachronic: Fusion | 292 c.2.1\|a.100.1 (0.0693642) -> c.2.1\|a.100.1& (0.5086705): c.14.1\|c.2.1\|a.100.1& (0.6416185) b.84.1\|a.9.1\|c.43.1 (0.3930636) -> b.84.1&\|a.9.1\|c.43.1 (0.4739884): b.84.1&\|a.9.1\|c.43.1\|c.3.1\|d.87.1 (0.6763006) b.84.1&\|a.9.1\|c.43.1 (0.4739884) -> b.84.1\|a.9.1\|c.43.1\|c.3.1\|d.87.1 (0.6763006): b.84.1&\|a.9.1\|c.43.1\|c.3.1\|d.87.1 (0.6763006) | 142 c.2.1\|d.81.1 (0.0867052) -> c.73.1\|d.58.18& (0.3179191): c.73.1\|d.58.18&\|c.2.1\|d.81.1 (0.5202312) c.84.1&\|d.129.2 (0.1676301) -> c.68.1\|b.82.1 (0.6127167): c.84.1&\|d.129.2\|c.68.1\|b.82.1 (0.6763006) c.3.1\|d.87.1 (0.1560694) -> b.84.1&\|a.9.1\|c.43.1 (0.4739884): b.84.1&\|a.9.1\|c.43.1\|c.3.1\|d.87.1 (0.6763006) | 434 |
| Fission | 882 b.23.1&\|g.12.1 (0.6589595) -> b.23.1\|g.12.1 (0.6763006): b.23.1&\|g.12.1\|b.47.1 (0.6589595) g.12.1&\|b.23.1 (0.6647399) -> b.23.1\|g.12.1 (0.6763006): g.12.1&\|b.23.1&\|g.12.1 (0.6647399) g.12.1\|g.3.14\|g.12.1 (0.6647399) -> g.12.1&\|g.3.11 (0.6763006): g.12.1\|g.3.14\|g.12.1&\|g.3.11& (0.6647399) | 468 g.18.1\|b.23.1\|g.18.1 (0.6647399) -> b.23.1\|g.18.1\|b.23.1\|g.18.1& (0.6763006): b.23.1\|g.18.1\|b.23.1\|g.18.1\|b.23.1\|g.18.1& (0.6647399) c.62.1\|g.3.11 (0.6705202) -> g.3.11\|g.18.1& (0.6763006): c.62.1\|g.3.11\|g.18.1& (0.6647399) c.43.1&\|e.23.1 (0.6763006) -> e.23.1\|a.28.1\|c.69.1 (0.6820809): c.43.1&\|e.23.1\|a.28.1\|c.69.1 (0.5953757) | 1,350 |
| Fusion/Fission | 199 b.23.1&\|g.12.1 (0.6589595) -> b.23.1\|g.12.1 (0.6763006): g.18.1&\|b.23.1&\|g.12.1 (0.6647399) g.12.1&\|b.23.1& (0.6589595) -> b.23.1\|g.12.1 (0.6763006): g.12.1&\|b.23.1&\|g.12.1 (0.6647399) b.23.1&\|g.12.1 (0.6589595) -> b.23.1\|g.12.1 (0.6763006): g.12.1&\|b.23.1&\|g.12.1 (0.6647399) | 143 b.84.1&\|a.9.1\|c.43.1 (0.4739884) -> a.9.1\|c.43.1 (0.6820809): b.84.1&\|a.9.1\|c.43.1\|c.3.1\|d.87.1 (0.6763006) c.43.1&\|e.23.1\|a.28.1\|c.69.1 (0.5953757) -> e.23.1\|a.28.1\|c.69.1 (0.6820809): c.43.1&\|e.23.1\|a.28.1\|c.43.1&\|e.23.1\|a.28.1\|c.43.1&\|e.23.1\|a.28.1\|c.43.1&\|e.23.1\|a.28.1\|c.43.1&\|e.23.1\|a.28.1\|c.43.1&\|e.23.1\|a.28.1\|c.43.1&\|e.23.1\|a.28.1\|c.43.1&\|e.23.1\|a.28.1\|c.69.1 (0.6763006) c.43.1&\|e.23.1\|a.28.1\|c.69.1 (0.5953757) -> a.28.1\|c.69.1 (0.6820809): c.43.1&\|e.23.1\|a.28.1\|c.43.1&\|e.23.1\|a.28.1\|c.43.1&\|e.23.1\|a.28.1\|c.43.1&\|e.23.1\|a.28.1\|c.43.1&\|e.23.1\|a.28.1\|c.43.1&\|e.23.1\|a.28.1\|c.43.1&\|e.23.1\|a.28.1\|c.43.1&\|e.23.1\|a.28.1\|c.69.1 (0.6763006) | 342 |
| Overlap(s) | Fusion: 425 c.2.1\|a.100.1 (0.0693642) -> c.2.1\|a.100.1& (0.5086705): c.14.1\|c.2.1\|a.100.1& (0.6416185) g.18.1\|b.23.1\|g.18.1 (0.6647399) -> b.23.1\|g.18.1\|b.23.1\|g.18.1& (0.6763006): b.23.1\|g.18.1\|b.23.1\|g.18.1\|b.23.1\|g.18.1& (0.6647399) b.84.1\|a.9.1\|c.43.1 (0.3930636) -> b.84.1&\|a.9.1\|c.43.1 (0.4739884): b.84.1&\|a.9.1\|c.43.1\|c.3.1\|d.87.1 (0.6763006) | Fission: 827 b.23.1&\|g.12.1 (0.6589595) -> b.23.1\|g.12.1 (0.6763006): b.23.1&\|g.12.1\|b.47.1 (0.6589595) b.23.1&\|g.12.1 (0.6589595) -> b.23.1\|g.12.1 (0.6763006): g.18.1&\|b.23.1&\|g.12.1 (0.6647399) b.23.1&\|g.12.1 (0.6589595) -> b.23.1\|g.12.1 (0.6763006): g.12.1&\|b.23.1&\|g.12.1 (0.6647399) | 1,252 |
| Sum total | 1,593 | 843 | 2,436 |
| Grand total | 9,431 | 25,589 | 35,020 |
| 11) Domain Categories | | | |
| Donors | 371 - c.37.1 (0.0000000); e.7.1 (0.1329480); a.128.1 (0.1387283) | | |
| Acceptors | 383 - c.56.2 (0.1907514); c.12.1 (0.2312139); c.18.1 (0.2369942) | | |
| Donors/Acceptors | 1,599 - c.2.1 (0.0057803); c.67.1 (0.0115607); c.66.1 (0.0173410) | | |
| Peers | 12 - c.114.1 (0.3583815); f.25.1 (0.3641618); d.52.4 (0.6763006) | | |
| Noncombinables | 3,797 - d.41.2\|c.1.17 (0.1734104); c.37.1\|b.43.3\|d.58.11\|d.14.1\|d.58.11 (0.1791908); g.41.8 (0.1965318) | | |

**Supplementary Table 3: Cumulative final counts per categories of nodes (domains, supradomains and multidomains) and connecting arcs in PAX at age *nd* = 1.** The pairwise adjacency network (PAX) is a subset of PX and defines connectivity between domains, supradomains and multidomains based on contiguously adjacent pairwise events in the same or other proteins. The pairwise events were not differentiated based on a left-to-right or right-to-left permutation. The algorithm designed to generate the adjacent domain/supradomain pairs had the same time complexity (and execution time) as that in the case of PX because the adjacency constraint was placed beyond the step that determined the parent multidomain arrays. The space complexity of the algorithm, however, was reduced to <= 128MB. Definitions of a parent multidomain and of the fusion, fission and fusion/fission events were borrowed from PX. Also, possible self-pairs that represented adjacent recurrence of a domain in a domain repeat or that of a domain/supradomain in a parent multidomain were not included. In order to determine a part of repeat match, the contiguous domain/supradomain pair was considered as a whole, instead of individual child nodes. This ensured the contiguity of the domain/supradomain pair because the domains at the carboxyl or amino end of the whole pair (permuted either of the two ways) occurred in a parent multidomain with repeats for a part of repeat match. However, if available, priority was given to a complete match type. Notably, there were no possible overlaps defined in the connectivity between any adjacent domains/supradomains.

| **Self-set comparisons** | | | |
| --- | --- | --- | --- |
| **1a) Domains in multidomains with no repeats (3,483 /\ 3,483 = 3,483 parents with 3,364 common)** | | | |
|  | Arcs with part of repeat match | Arcs with complete match | Arcs Total |
| **Contemporaneous: Fusion** | 0 | 30 | 30 |
| **Fission** | 0 | 94 | 94 |
| **Fusion/Fission** | 0 | 3 | 3 |
| **Diachronic: Fusion** | 0 | 1,230 c.37.1 (0.0000000) -> c.23.16 (0.0809249): c.37.1\|c.23.16 (0.2369942) c.37.1 (0.0000000) -> a.4.5 (0.0751445): c.37.1\|a.4.5 (0.2716763) d.153.1 (0.1445087) -> c.80.1 (0.1618497): d.153.1\|c.80.1 (0.3005780) | 1,230 |
| **Fission** | 0 | 1,438 c.31.1 (0.2543353) -> c.36.1 (0.2601156): c.36.1\|c.31.1\|c.36.1 (0.2023121) c.116.1 (0.3526012) -> b.122.1 (0.3641618): b.122.1\|c.116.1 (0.3294798) d.307.1 (0.6531792) -> e.18.1 (0.6589595): d.307.1\|e.18.1 (0.5895954) | 1,438 |
| **Fusion/Fission** | 0 | 1,570 c.37.1 (0.0000000) -> b.43.3 (0.2890173): c.37.1\|b.43.3\|d.58.11\|d.14.1\|d.58.11 (0.1791908) c.37.1 (0.0000000) -> b.43.3 (0.2890173): c.37.1\|b.43.3\|b.44.1 (0.1965318) c.37.1 (0.0000000) -> g.39.1 (0.3468208): g.39.1\|c.37.1 (0.3121387) | 1,570 |
| **Sum total** | 0 | 4,365 | 4,365 |
| **1b) Domains in multidomains with repeats (777 /\ 777 = 777 parents with 765 common)** | | | |
|  | Arcs with part of repeat match | Arcs with complete match | Arcs Total |
| **Contemporaneous: Fusion** | 3 | 2 | 5 |
| **Fission** | 27 | 9 | 36 |
| **Fusion/Fission** | 0 | 0 | 0 |
| **Diachronic: Fusion** | 118 b.40.4 (0.0520231) -> b.34.5 (0.1849711): b.34.5\|b.40.4& (0.3005780) c.55.1 (0.1907514) -> a.211.1 (0.2543353): c.55.1&\|a.211.1 (0.4450867) c.47.1 (0.0231214) -> c.3.1 (0.1849711): c.47.1&\|c.3.1 (0.4508671) | 59 c.69.1 (0.0346821) -> a.28.1 (0.3526012): c.43.1&\|e.23.1\|a.28.1\|c.69.1 (0.5953757) e.23.1 (0.1445087) -> a.28.1 (0.3526012): c.43.1&\|e.23.1\|a.28.1\|c.69.1 (0.5953757) c.2.1 (0.0057803) -> c.14.1 (0.1387283): c.14.1\|c.2.1\|a.100.1& (0.6416185) | 177 |
| **Fission** | 385 a.4.1 (0.6647399) -> b.82.4 (0.6878613): b.82.4\|a.4.1& (0.5549133) b.1.3 (0.6820809) -> c.6.1 (0.6878613): b.2.2\|b.1.3&\|c.6.1 (0.6820809) a.118.14 (0.6820809) -> d.37.1 (0.6936416): a.118.14\|d.37.1& (0.6763006) | 129 c.142.1 (0.6763006) -> d.15.13 (0.6820809): c.47.1&\|c.142.1\|d.15.13\|a.29.12 (0.6763006) a.29.12 (0.6763006) -> d.15.13 (0.6820809): c.47.1&\|c.142.1\|d.15.13\|a.29.12 (0.6763006) a.102.1 (0.6936416) -> b.2.2 (0.6994219): a.102.1\|b.2.2\|b.1.18&\|b.2.2 (0.6763006) | 514 |
| **Fusion/Fission** | 296 c.37.1 (0.0000000) -> d.315.1 (0.6763006): c.37.1&\|b.34.18\|c.37.1&\|d.315.1 (0.4913295) c.55.1 (0.1907514) -> d.22.1 (0.6763006): c.55.1&\|d.22.1 (0.6647399) c.37.1 (0.0000000) -> a.60.4 (0.6820809): a.60.4&\|c.37.1 (0.6763006) | 106 b.40.4 (0.0520231) -> a.171.1 (0.6820809): a.170.1\|b.40.4\|a.171.1\|b.40.4& (0.6763006) b.40.4 (0.0520231) -> a.170.1 (0.6820809): a.170.1\|b.40.4\|a.171.1\|b.40.4& (0.6763006) c.68.1 (0.0693642) -> d.129.2 (0.6878613): c.84.1&\|d.129.2\|c.68.1\|b.82.1 (0.6763006) | 402 |
| **Sum total** | 829 | 305 | 1,134 |
| **2) Domain repeats in multidomains with repeats (720 /\ 720 = 720 parents with 53 common)** | | | |
|  | Arcs with part of repeat match | Arcs with complete match | Arcs Total |
| **Contemporaneous: Fusion** | 0 | 1 | 1 |
| **Fission** | 0 | 4 | 4 |
| **Fusion/Fission** | 0 | 0 | 0 |
| **Diachronic: Fusion** | 0 | 2 d.37.1& (0.3641618) -> d.110.3& (0.6820809): d.37.1&\|d.110.3&\|c.37.1\|a.4.1 (0.6820809) d.37.1& (0.3641618) -> c.26.2& (0.4682081): f.20.1\|d.37.1&\|c.26.2& (0.7225434) | 2 |
| **Fission** | 0 | 31 d.106.1& (0.6763006) -> d.38.1& (0.6820809): c.2.1\|d.38.1&\|d.106.1& (0.6647399) g.3.15& (0.7225434) -> g.8.1& (0.7398844): g.8.1&\|g.3.15& (0.7109827) d.209.1& (0.7398844) -> c.62.1& (0.7687861): d.209.1&\|c.62.1& (0.6763006) | 31 |
| **Fusion/Fission** | 0 | 7 d.37.1& (0.3641618) -> d.110.3& (0.6820809): d.37.1&\|d.110.3&\|d.58.29\|c.1.33 (0.6763006) d.110.3& (0.6820809) -> c.23.1& (0.7283237): c.23.1&\|d.110.3&\|a.30.2\|d.122.1 (0.6878613) g.27.1& (0.6878613) -> g.60.1& (0.7919075): g.27.1&\|g.60.1&\|g.27.1 (0.7109827) | 7 |
| **Sum total** | 0 | 45 | 45 |
| **3a) Multidomains with no repeats in themselves (908 /\ 908 = 908 parents with 507 common)** | | | |
|  | Arcs with part of repeat match | Arcs with complete match | Arcs Total |
| **Contemporaneous: Fusion** | 0 | 19 | 19 |
| **Fission** | 0 | 14 | 14 |
| **Fusion/Fission** | 0 | 2 | 2 |
| **Diachronic: Fusion** | 0 | 132 d.79.4\|d.139.1 (0.3757226) -> d.284.1\|a.5.10\|d.79.4\|d.139.1 (0.6705202): c.2.1\|d.284.1\|a.5.10\|d.79.4\|d.139.1\|d.79.4\|d.139.1\|c.23.16 (0.6763006) b.43.4\|c.25.1 (0.3352601) -> c.81.1\|b.52.2 (0.3468208): c.81.1\|b.52.2\|b.43.4\|c.25.1\|d.15.4 (0.6763006) c.8.3\|c.23.16 (0.3121387) -> c.30.1\|d.142.1 (0.3179191): c.8.3\|c.23.16\|c.30.1\|d.142.1 (0.6763006) | 132 |
| **Fission** | 0 | 197 c.30.1\|d.142.1 (0.3179191) -> a.92.1\|c.30.1\|d.142.1 (0.6705202): c.30.1\|d.142.1\|a.92.1\|c.30.1\|d.142.1\|c.24.1 (0.3121387) d.58.1\|c.36.1 (0.6763006) -> c.23.5\|b.43.4 (0.6820809): c.36.1\|c.48.1\|c.64.1\|d.58.1\|c.36.1\|c.23.5\|b.43.4\|c.25.1 (0.6763006) d.142.1\|c.24.1 (0.6763006) -> d.142.1\|a.92.1\|c.30.1 (0.6878613): c.30.1\|d.142.1\|a.92.1\|c.30.1\|d.142.1\|c.24.1 (0.3121387) | 197 |
| **Fusion/Fission** | 0 | 146 d.79.4\|d.139.1 (0.3757226) -> d.284.1\|a.5.10\|d.79.4\|d.139.1 (0.6705202): d.284.1\|a.5.10\|d.79.4\|d.139.1\|d.79.4\|d.139.1\|c.23.16 (0.4219653) d.14.1\|d.101.1 (0.2716763) -> a.4.9\|d.14.1\|d.101.1\|d.51.1\|b.40.4 (0.6763006): d.14.1\|d.101.1\|a.4.9\|d.14.1\|d.101.1\|d.51.1\|b.40.4 (0.3352601) d.104.1\|c.51.1 (0.2312139) -> d.15.10\|d.67.1 (0.6763006): d.15.10\|d.67.1\|d.104.1\|c.51.1 (0.3468208) | 146 |
| **Sum total** | 0 | 510 | 510 |
| **3b) Multidomains with no repeats in multidomains with repeats (535 /\ 535 = 535 parents with 219 common)** | | | |
|  | Arcs with part of repeat match | Arcs with complete match | Arcs Total |
| **Contemporaneous: Fusion** | 3 | 1 | 4 |
| **Fission** | 5 | 0 | 5 |
| **Fusion/Fission** | 1 | 1 | 2 |
| **Diachronic: Fusion** | 8 c.3.1\|d.87.1 (0.1560694) -> b.84.1\|a.9.1\|c.43.1 (0.3930636): b.84.1&\|a.9.1\|c.43.1\|c.3.1\|d.87.1 (0.6763006) d.110.2\|d.110.3 (0.6763006) -> d.58.29\|c.1.33 (0.6878613): d.110.2&\|d.110.3\|d.58.29\|c.1.33 (0.6878613) d.110.3\|a.30.2 (0.6763006) -> d.122.1\|c.23.1 (0.7109827): c.23.1\|d.110.3&\|a.30.2\|d.122.1\|c.23.1 (0.7109827) | 3 b.68.5\|g.3.11\|b.68.5 (0.6763006) -> b.68.5\|g.3.11 (0.6820809): g.12.1&\|g.3.11&\|b.68.5\|g.3.11\|b.68.5\|g.3.11\|b.68.5\|g.3.11\|b.68.5 (0.6994219) b.68.5\|g.3.11\|b.68.5 (0.6763006) -> g.3.11\|b.68.5 (0.6994219): g.12.1&\|g.3.11&\|b.68.5\|g.3.11\|b.68.5\|g.3.11\|b.68.5\|g.3.11\|b.68.5 (0.6994219) b.68.5\|g.3.11 (0.6820809) -> b.68.5\|g.3.11\|b.68.5\|g.3.11\|b.68.5 (0.6994219): g.12.1&\|g.3.11&\|b.68.5\|g.3.11\|b.68.5\|g.3.11\|b.68.5\|g.3.11\|b.68.5 (0.6994219) | 11 |
| **Fission** | 26 d.110.2\|d.110.3 (0.6763006) -> c.23.1\|d.110.3 (0.6994219): c.23.1\|d.110.3\|d.110.2\|d.110.3&\|a.30.2\|d.122.1 (0.6763006) d.110.2\|d.110.3 (0.6763006) -> c.23.1\|d.110.3 (0.6994219): c.23.1&\|d.110.3\|d.110.2\|d.110.3\|d.58.29\|c.1.33 (0.6763006) d.58.29\|c.1.33 (0.6878613) -> c.23.1\|d.110.3 (0.6994219): c.23.1&\|d.110.3\|d.58.29\|c.1.33 (0.6878613) | 15 d.110.2\|d.110.3 (0.6763006) -> d.110.2\|a.30.2\|d.122.1\|c.23.1 (0.6878613): d.110.3&\|d.110.2\|d.110.3\|d.110.2\|a.30.2\|d.122.1\|c.23.1 (0.6705202) d.110.2\|d.110.3 (0.6763006) -> d.58.29\|c.1.33 (0.6878613): c.23.1&\|d.110.3\|d.110.2\|d.110.3\|d.58.29\|c.1.33 (0.6763006) d.110.3\|d.110.2 (0.6763006) -> d.110.3\|d.58.29\|c.1.33 (0.7052023): c.23.1&\|d.110.3\|d.110.2\|d.110.3\|d.58.29\|c.1.33 (0.6763006) | 41 |
| **Fusion/Fission** | 20 d.110.3\|d.110.2 (0.6763006) -> d.58.29\|c.1.33 (0.6878613): d.110.3&\|d.110.2\|d.58.29\|c.1.33 (0.6820809) d.110.2\|d.110.3 (0.6763006) -> c.23.1\|d.110.3 (0.6994219): c.23.1\|d.110.3\|d.110.2\|d.110.3& (0.6878613) c.2.1\|d.81.1 (0.0867052) -> c.73.1\|d.58.18 (0.7052023): c.2.1\|d.81.1\|c.73.1\|d.58.18& (0.6936416) | 6 c.3.1\|d.87.1 (0.1560694) -> a.9.1\|c.43.1 (0.6820809): b.84.1&\|a.9.1\|c.43.1\|c.3.1\|d.87.1 (0.6763006) d.110.3\|a.30.2 (0.6763006) -> d.122.1\|c.23.1 (0.7109827): c.23.1&\|d.110.3\|a.30.2\|d.122.1\|c.23.1 (0.6820809) d.110.3\|a.30.2 (0.6763006) -> d.122.1\|c.23.1 (0.7109827): d.110.2&\|d.110.3\|a.30.2\|d.122.1\|c.23.1 (0.7052023) | 26 |
| **Sum total** | 63 | 26 | 89 |
| **4) Multidomains with repeats in themselves (229 /\ 229 = 229 parents with 110 common)** | | | |
|  | Arcs with part of repeat match | Arcs with complete match | Arcs Total |
| **Contemporaneous: Fusion** | 8 | 32 | 40 |
| **Fission** | 4 | 9 | 13 |
| **Fusion/Fission** | 9 | 41 | 50 |
| **Diachronic: Fusion** | 28 b.1.1\|b.1.2&\|b.1.1\|b.1.2&\|b.1.1\|b.1.2& (0.6589595) -> b.1.1\|b.1.2&\|b.1.1\|b.1.2 (0.6763006): b.1.1\|b.1.2&\|b.1.1\|b.1.2&\|b.1.1\|b.1.2&\|b.1.1\|b.1.2&\|b.1.1\|b.1.2& (0.6763006) b.1.1\|b.1.2&\|b.1.1\|b.1.2 (0.6763006) -> b.1.2&\|b.1.1\|b.1.2& (0.6820809): b.1.2&\|b.1.1\|b.1.2&\|b.1.1\|b.1.2&\|b.1.1\|b.1.2&\|b.1.1\|b.1.2& (0.6820809) e.23.1\|a.28.1\|c.43.1&\|e.23.1 (0.6763006) -> c.43.1\|e.23.1\|a.28.1\|c.43.1& (0.6820809): c.43.1&\|e.23.1\|a.28.1\|c.43.1&\|e.23.1\|a.28.1\|c.43.1&\|e.23.1\|a.28.1\|c.43.1&\|e.23.1\|a.28.1\|c.69.1 (0.6878613) | 79 c.43.1&\|e.23.1\|a.28.1\|c.69.1 (0.5953757) -> c.43.1&\|e.23.1\|a.28.1\|c.43.1&\|e.23.1\|a.28.1\|c.43.1&\|e.23.1\|a.28.1 (0.6763006): c.43.1&\|e.23.1\|a.28.1\|c.43.1&\|e.23.1\|a.28.1\|c.43.1&\|e.23.1\|a.28.1\|c.43.1&\|e.23.1\|a.28.1\|c.43.1&\|e.23.1\|a.28.1\|c.43.1&\|e.23.1\|a.28.1\|c.43.1&\|e.23.1\|a.28.1\|c.43.1&\|e.23.1\|a.28.1\|c.69.1 (0.6763006) c.43.1&\|e.23.1\|a.28.1\|c.69.1 (0.5953757) -> e.23.1\|a.28.1\|c.43.1&\|e.23.1\|a.28.1 (0.6763006): c.43.1&\|e.23.1\|a.28.1\|c.43.1&\|e.23.1\|a.28.1\|c.43.1&\|e.23.1\|a.28.1\|c.43.1&\|e.23.1\|a.28.1\|c.43.1&\|e.23.1\|a.28.1\|c.43.1&\|e.23.1\|a.28.1\|c.43.1&\|e.23.1\|a.28.1\|c.43.1&\|e.23.1\|a.28.1\|c.69.1 (0.6763006) c.43.1&\|e.23.1\|a.28.1\|c.69.1 (0.5953757) -> c.43.1&\|e.23.1\|a.28.1\|c.43.1&\|e.23.1\|a.28.1\|c.43.1&\|e.23.1\|a.28.1 (0.6763006): c.43.1&\|e.23.1\|a.28.1\|c.43.1&\|e.23.1\|a.28.1\|c.43.1&\|e.23.1\|a.28.1\|c.43.1&\|e.23.1\|a.28.1\|c.43.1&\|e.23.1\|a.28.1\|c.43.1&\|e.23.1\|a.28.1\|c.43.1&\|e.23.1\|a.28.1\|c.43.1&\|e.23.1\|a.28.1\|c.43.1&\|e.23.1\|a.28.1\|c.69.1 (0.6763006) | 107 |
| **Fission** | 47 e.23.1\|a.28.1\|c.43.1&\|e.23.1 (0.6763006) -> c.43.1\|e.23.1\|a.28.1\|c.43.1& (0.6820809): c.43.1&\|e.23.1\|a.28.1\|c.43.1&\|e.23.1\|a.28.1\|c.43.1&\|e.23.1\|a.28.1 (0.6763006) e.23.1\|a.28.1\|c.43.1&\|e.23.1\|a.28.1 (0.6763006) -> c.43.1\|e.23.1\|a.28.1\|c.43.1& (0.6820809): c.43.1&\|e.23.1\|a.28.1\|c.43.1&\|e.23.1\|a.28.1\|c.43.1&\|e.23.1\|a.28.1 (0.6763006) c.43.1&\|e.23.1\|a.28.1\|c.43.1&\|e.23.1\|a.28.1\|c.43.1 (0.6763006) -> a.28.1\|c.43.1&\|e.23.1\|a.28.1\|c.43.1&\|e.23.1\|a.28.1 (0.6820809): c.43.1&\|e.23.1\|a.28.1\|c.43.1&\|e.23.1\|a.28.1\|c.43.1&\|e.23.1\|a.28.1\|c.43.1&\|e.23.1\|a.28.1\|c.43.1&\|e.23.1\|a.28.1\|c.43.1&\|e.23.1\|a.28.1\|c.43.1&\|e.23.1\|a.28.1\|c.43.1&\|e.23.1\|a.28.1\|c.69.1 (0.6763006) | 243 e.23.1\|a.28.1\|c.43.1&\|e.23.1 (0.6763006) -> e.23.1\|a.28.1\|c.43.1& (0.6820809): c.43.1&\|e.23.1\|a.28.1\|c.43.1&\|e.23.1\|a.28.1\|c.43.1&\|e.23.1\|a.28.1 (0.6763006) e.23.1\|a.28.1\|c.43.1&\|e.23.1\|a.28.1 (0.6763006) -> e.23.1\|a.28.1\|c.43.1& (0.6820809): c.43.1&\|e.23.1\|a.28.1\|c.43.1&\|e.23.1\|a.28.1\|c.43.1&\|e.23.1\|a.28.1 (0.6763006) a.28.1\|c.43.1& (0.6763006) -> e.23.1\|a.28.1\|c.43.1& (0.6820809): c.43.1&\|e.23.1\|a.28.1\|c.43.1&\|e.23.1\|a.28.1\|c.43.1&\|e.23.1\|a.28.1 (0.6763006) | 290 |
| **Fusion/Fission** | 13 b.1.2&\|b.1.1\|b.1.2 (0.6763006) -> b.1.2&\|b.1.1 (0.6936416): b.1.2&\|b.1.1\|b.1.2&\|b.1.1\|b.1.2&\|b.1.1\|b.1.2&\|b.1.1\|b.1.2& (0.6820809) b.1.2&\|b.1.1\|b.1.2&\|b.1.1\|b.1.2 (0.6763006) -> b.1.2&\|b.1.1 (0.6936416): b.1.2&\|b.1.1\|b.1.2&\|b.1.1\|b.1.2&\|b.1.1\|b.1.2&\|b.1.1\|b.1.2& (0.6820809) b.1.1\|b.1.2&\|b.1.1\|b.1.2&\|b.1.1\|b.1.2& (0.6589595) -> b.1.2\|b.1.1\|b.1.2& (0.6994219): b.1.1\|b.1.2&\|b.1.1\|b.1.2&\|b.1.1\|b.1.2&\|b.1.1\|b.1.2&\|b.1.1\|b.1.2& (0.6763006) | 62 c.43.1&\|e.23.1\|a.28.1\|c.69.1 (0.5953757) -> a.28.1\|c.43.1&\|e.23.1\|a.28.1\|c.43.1&\|e.23.1\|a.28.1 (0.6820809): c.43.1&\|e.23.1\|a.28.1\|c.43.1&\|e.23.1\|a.28.1\|c.43.1&\|e.23.1\|a.28.1\|c.43.1&\|e.23.1\|a.28.1\|c.43.1&\|e.23.1\|a.28.1\|c.43.1&\|e.23.1\|a.28.1\|c.43.1&\|e.23.1\|a.28.1\|c.43.1&\|e.23.1\|a.28.1\|c.69.1 (0.6763006) c.43.1&\|e.23.1\|a.28.1\|c.69.1 (0.5953757) -> a.28.1\|c.43.1&\|e.23.1\|a.28.1\|c.43.1&\|e.23.1\|a.28.1 (0.6820809): c.43.1&\|e.23.1\|a.28.1\|c.43.1&\|e.23.1\|a.28.1\|c.43.1&\|e.23.1\|a.28.1\|c.43.1&\|e.23.1\|a.28.1\|c.43.1&\|e.23.1\|a.28.1\|c.43.1&\|e.23.1\|a.28.1\|c.43.1&\|e.23.1\|a.28.1\|c.43.1&\|e.23.1\|a.28.1\|c.43.1&\|e.23.1\|a.28.1\|c.69.1 (0.6763006) b.1.1\|b.1.2&\|b.1.1\|b.1.2&\|b.1.1\|b.1.2& (0.6589595) -> b.1.2&\|b.1.1\|b.1.2& (0.6820809): b.1.1\|b.1.2&\|b.1.1\|b.1.2&\|b.1.1\|b.1.2&\|b.1.1\|b.1.2&\|b.1.1\|b.1.2& (0.6763006) | 75 |
| **Sum total** | 109 | 466 | 575 |
| **Paired set comparisons** | | | |
| **5) Domains and domain repeats in multidomains with repeats (777 /\ 720 = 720 parents with 720 common)** | | | |
|  | Arcs with part of repeat match | Arcs with complete match | Arcs Total |
| **Contemporaneous: Fusion** | 0 | 14 | 14 |
| **Fission** | 1 | 29 | 30 |
| **Fusion/Fission** | 0 | 1 | 1 |
| **Diachronic: Fusion** | 4 c.23.1 (0.4046243) -> d.110.3& (0.6820809): c.23.1&\|d.110.3&\|a.30.2\|d.122.1 (0.6878613) a.4.5 (0.0751445) -> a.142.1& (0.6878613): a.4.5&\|a.142.1&\|c.44.2\|d.112.1 (0.6936416) c.26.2 (0.1271676) -> d.37.1& (0.3641618): f.20.1\|d.37.1&\|c.26.2& (0.7225434) | 128 c.55.1& (0.0462428) -> a.211.1 (0.2543353): c.55.1&\|a.211.1 (0.4450867) c.37.1& (0.1907514) -> c.52.1 (0.3699422): c.37.1&\|c.52.1 (0.4682081) c.2.1 (0.0057803) -> d.58.18& (0.3294798): c.73.1\|d.58.18&\|c.2.1\|d.81.1 (0.5202312) | 132 |
| **Fission** | 65 d.110.3& (0.6820809) -> d.37.1 (0.6936416): d.37.1&\|d.110.3&\|d.58.29\|c.1.33 (0.6763006) d.110.3& (0.6820809) -> d.37.1 (0.6936416): d.37.1&\|d.110.3&\|c.37.1\|a.4.1 (0.6820809) d.110.2& (0.6820809) -> d.110.3 (0.7225434): d.110.3&\|d.110.2&\|a.211.1 (0.6763006) | 393 d.58.18& (0.3294798) -> c.73.1 (0.3641618): c.73.1\|d.58.18& (0.3179191) f.7.1& (0.6763006) -> a.118.4 (0.6820809): f.7.1\|a.118.4\|f.7.1& (0.6763006) a.4.1& (0.6011561) -> b.82.4 (0.6878613): b.82.4\|a.4.1& (0.5549133) | 458 |
| **Fusion/Fission** | 15 d.38.1 (0.1271676) -> d.106.1& (0.6763006): c.2.1\|d.38.1&\|d.106.1& (0.6647399) d.37.1& (0.3641618) -> d.110.3 (0.7225434): d.37.1&\|d.110.3&\|d.58.29\|c.1.33 (0.6763006) d.37.1& (0.3641618) -> d.110.3 (0.7225434): d.37.1&\|d.110.3&\|c.37.1\|a.4.1 (0.6820809) | 243 b.34.5 (0.1849711) -> b.40.4& (0.6358382): b.34.5\|b.40.4& (0.3005780) c.37.1& (0.1907514) -> d.315.1 (0.6763006): c.37.1&\|b.34.18\|c.37.1&\|d.315.1 (0.4913295) c.37.1 (0.0000000) -> h.1.26& (0.6763006): c.37.1\|h.1.26& (0.6647399) | 258 |
| **Sum total** | 85 | 808 | 893 |
| **6a) Domains and multidomains with no repeats in the latter (3,483 /\ 908 = 908 parents with 908 common)** | | | |
|  | Arcs with part of repeat match | Arcs with complete match | Arcs Total |
| **Contemporaneous: Fusion** | 0 | 13 | 13 |
| **Fission** | 0 | 30 | 30 |
| **Fusion/Fission** | 0 | 4 | 4 |
| **Diachronic: Fusion** | 0 | 641 c.23.16 (0.0809249) -> d.79.4\|d.139.1 (0.3757226): d.284.1\|a.5.10\|d.79.4\|d.139.1\|d.79.4\|d.139.1\|c.23.16 (0.4219653) c.2.1 (0.0057803) -> c.65.1\|b.46.1 (0.3410405): c.65.1\|b.46.1\|c.2.1 (0.5780347) c.23.16 (0.0809249) -> a.46.2\|c.27.1 (0.3236994): c.23.16\|a.46.2\|c.27.1 (0.5953757) | 641 |
| **Fission** | 0 | 853 c.30.1\|d.142.1 (0.3179191) -> c.24.1 (0.5491329): c.30.1\|d.142.1\|a.92.1\|c.30.1\|d.142.1\|c.24.1 (0.3121387) c.23.1 (0.4046243) -> c.37.1\|a.4.1 (0.5780347): c.23.1\|c.37.1\|a.4.1 (0.3699422) c.24.1 (0.5491329) -> a.92.1\|c.30.1\|d.142.1 (0.6705202): c.30.1\|d.142.1\|a.92.1\|c.30.1\|d.142.1\|c.24.1 (0.3121387) | 853 |
| **Fusion/Fission** | 0 | 899 b.40.4 (0.0520231) -> d.14.1\|d.101.1\|a.4.9\|d.14.1\|d.101.1\|d.51.1 (0.6763006): d.14.1\|d.101.1\|a.4.9\|d.14.1\|d.101.1\|d.51.1\|b.40.4 (0.3352601) d.14.1 (0.1791908) -> d.101.1\|a.4.9\|d.14.1 (0.6763006): d.14.1\|d.101.1\|a.4.9\|d.14.1\|d.101.1\|d.51.1\|b.40.4 (0.3352601) c.23.16 (0.0809249) -> d.284.1\|a.5.10\|d.79.4\|d.139.1\|d.79.4\|d.139.1 (0.6763006): d.284.1\|a.5.10\|d.79.4\|d.139.1\|d.79.4\|d.139.1\|c.23.16 (0.4219653) | 899 |
| **Sum total** | 0 | 2,440 | 2,440 |
| **6b) Domains and multidomains with no repeats in multidomains with repeats (777 /\ 535 = 535 parents with 535 common)** | | | |
|  | Arcs with part of repeat match | Arcs with complete match | Arcs Total |
| **Contemporaneous: Fusion** | 0 | 0 | 0 |
| **Fission** | 13 | 3 | 16 |
| **Fusion/Fission** | 1 | 0 | 1 |
| **Diachronic: Fusion** | 63 c.2.1\|a.100.1 (0.0693642) -> c.14.1 (0.1387283): c.14.1\|c.2.1\|a.100.1& (0.6416185) c.3.1 (0.1849711) -> b.84.1\|a.9.1\|c.43.1 (0.3930636): b.84.1&\|a.9.1\|c.43.1\|c.3.1\|d.87.1 (0.6763006) c.23.1 (0.4046243) -> d.110.2\|d.110.3 (0.6763006): c.23.1&\|d.110.2\|d.110.3&\|d.58.29\|c.1.33 (0.6763006) | 23 c.23.1 (0.4046243) -> d.110.3\|d.110.2 (0.6763006): c.23.1\|d.110.3\|d.110.2\|d.110.3&\|a.30.2\|d.122.1 (0.6763006) c.1.33 (0.5028902) -> d.110.3\|d.110.2\|d.110.3\|d.58.29 (0.6763006): c.23.1&\|d.110.3\|d.110.2\|d.110.3\|d.58.29\|c.1.33 (0.6763006) c.23.1 (0.4046243) -> d.110.3\|a.30.2 (0.6763006): c.23.1\|d.110.3\|a.30.2\|d.122.1\|c.23.1&\|a.24.10 (0.6763006) | 86 |
| **Fission** | 202 d.110.3\|d.110.2 (0.6763006) -> a.30.2 (0.6820809): d.110.3&\|d.110.2\|a.30.2\|d.122.1 (0.6763006) d.110.3\|d.110.2 (0.6763006) -> a.30.2 (0.6820809): c.23.1\|d.110.3&\|d.110.2\|a.30.2\|d.122.1 (0.6763006) c.66.1\|d.108.1 (0.6763006) -> c.78.2 (0.6878613): c.66.1\|d.108.1\|c.78.2& (0.6763006) | 106 d.110.3\|d.110.2 (0.6763006) -> a.30.2 (0.6820809): d.110.3&\|d.110.2\|d.110.3\|d.110.2\|a.30.2\|d.122.1\|c.23.1 (0.6705202) d.110.2\|d.110.3 (0.6763006) -> a.30.2 (0.6820809): d.110.3&\|d.110.2\|d.110.3\|a.30.2\|d.122.1 (0.6705202) a.170.1\|b.40.4 (0.6763006) -> a.171.1 (0.6820809): a.170.1\|b.40.4\|a.171.1\|b.40.4& (0.6763006) | 308 |
| **Fusion/Fission** | 145 a.28.1 (0.3526012) -> c.43.1\|e.23.1 (0.6820809): c.43.1&\|e.23.1\|a.28.1\|c.69.1 (0.5953757) a.28.1 (0.3526012) -> c.43.1\|e.23.1 (0.6820809): c.43.1&\|e.23.1\|a.28.1\|c.43.1&\|e.23.1\|a.28.1\|c.43.1&\|e.23.1\|a.28.1 (0.6763006) a.28.1 (0.3526012) -> c.43.1\|e.23.1 (0.6820809): e.23.1\|a.28.1\|c.43.1&\|e.23.1\|a.28.1\|c.43.1&\|e.3.1 (0.6763006) | 77 c.23.1 (0.4046243) -> d.110.3\|d.110.2\|a.30.2\|d.122.1 (0.6763006): d.110.3&\|d.110.2\|d.110.3\|d.110.2\|a.30.2\|d.122.1\|c.23.1 (0.6705202) e.23.1 (0.1445087) -> a.28.1\|c.69.1 (0.6820809): c.43.1&\|e.23.1\|a.28.1\|c.69.1 (0.5953757) c.3.1 (0.1849711) -> a.9.1\|c.43.1 (0.6820809): b.84.1&\|a.9.1\|c.43.1\|c.3.1\|d.87.1 (0.6763006) | 222 |
| **Sum total** | 424 | 209 | 633 |
| **7) Domains and multidomains with repeats in the latter (777 /\ 229 = 229 parents with 229 common)** | | | |
|  | Arcs with part of repeat match | Arcs with complete match | Arcs Total |
| **Contemporaneous: Fusion** | 0 | 2 | 2 |
| **Fission** | 0 | 5 | 5 |
| **Fusion/Fission** | 0 | 0 | 0 |
| **Diachronic: Fusion** | 16 a.28.1 (0.3526012) -> c.43.1&\|e.23.1\|a.28.1\|c.43.1&\|e.23.1\|a.28.1\|c.43.1 (0.6763006): c.43.1&\|e.23.1\|a.28.1\|c.43.1&\|e.23.1\|a.28.1\|c.43.1&\|e.23.1\|a.28.1\|c.43.1&\|e.23.1\|a.28.1\|c.43.1&\|e.23.1\|a.28.1\|c.43.1&\|e.23.1\|a.28.1\|c.43.1&\|e.23.1\|a.28.1\|c.43.1&\|e.23.1\|a.28.1\|c.69.1 (0.6763006) a.28.1 (0.3526012) -> c.43.1&\|e.23.1\|a.28.1\|c.43.1&\|e.23.1\|a.28.1\|c.43.1 (0.6763006): c.43.1&\|e.23.1\|a.28.1\|c.43.1&\|e.23.1\|a.28.1\|c.43.1&\|e.23.1\|a.28.1\|c.43.1&\|e.23.1\|a.28.1\|c.43.1&\|e.23.1\|a.28.1\|c.43.1&\|e.23.1\|a.28.1\|c.43.1&\|e.23.1\|a.28.1\|c.43.1&\|e.23.1\|a.28.1\|c.43.1&\|e.23.1\|a.28.1\|c.69.1 (0.6763006) a.28.1 (0.3526012) -> c.43.1&\|e.23.1\|a.28.1\|c.43.1&\|e.23.1\|a.28.1\|c.43.1 (0.6763006): c.43.1&\|e.23.1\|a.28.1\|c.43.1&\|e.23.1\|a.28.1\|c.43.1&\|e.23.1\|a.28.1\|c.43.1&\|e.23.1\|a.28.1\|c.43.1&\|e.23.1\|a.28.1\|c.69.1 (0.6763006) | 153 c.2.1 (0.0057803) -> c.73.1\|d.58.18& (0.3179191): c.73.1\|d.58.18&\|c.2.1\|d.81.1 (0.5202312) c.14.1 (0.1387283) -> c.2.1\|a.100.1& (0.5086705): c.14.1\|c.2.1\|a.100.1& (0.6416185) a.28.1 (0.3526012) -> e.23.1\|a.28.1\|c.43.1&\|e.23.1 (0.6763006): c.43.1&\|e.23.1\|a.28.1\|c.43.1&\|e.23.1\|a.28.1\|c.43.1&\|e.23.1\|a.28.1 (0.6763006) | 169 |
| **Fission** | 135 d.37.1 (0.6936416) -> d.110.3&\|c.37.1\|a.4.1 (0.6994219): d.37.1&\|d.110.3&\|c.37.1\|a.4.1 (0.6820809) b.23.1\|g.3.11& (0.6763006) -> b.61.1 (0.7052023): b.47.1\|b.23.1&\|g.3.11&\|b.61.1 (0.6763006) d.37.1 (0.6936416) -> d.110.3&\|d.58.29\|c.1.33 (0.7109827): d.37.1&\|d.110.3&\|d.58.29\|c.1.33 (0.6763006) | 163 c.23.1\|d.110.3&\|d.110.2 (0.6763006) -> a.30.2 (0.6820809): c.23.1\|d.110.3&\|d.110.2\|a.30.2\|d.122.1 (0.6763006) a.30.2 (0.6820809) -> c.23.1\|d.110.3\|d.110.2\|d.110.3& (0.6878613): c.23.1\|d.110.3\|d.110.2\|d.110.3&\|a.30.2\|d.122.1 (0.6763006) b.23.1\|g.3.11& (0.6763006) -> b.61.1 (0.7052023): g.3.11\|b.23.1\|g.3.11&\|b.61.1 (0.6763006) | 298 |
| **Fusion/Fission** | 66 c.37.1 (0.0000000) -> c.37.1&\|b.34.18 (0.6763006): c.37.1&\|b.34.18\|c.37.1&\|d.315.1 (0.4913295) e.23.1 (0.1445087) -> c.43.1\|e.23.1\|a.28.1\|c.43.1& (0.6820809): c.43.1&\|e.23.1\|a.28.1\|c.43.1&\|e.23.1\|a.28.1\|c.43.1&\|e.23.1\|a.28.1 (0.6763006) e.3.1 (0.3872832) -> c.43.1\|e.23.1\|a.28.1\|c.43.1& (0.6820809): e.23.1\|a.28.1\|c.43.1&\|e.23.1\|a.28.1\|c.43.1&\|e.3.1 (0.6763006) | 237 a.28.1 (0.3526012) -> c.43.1&\|e.23.1 (0.6763006): c.43.1&\|e.23.1\|a.28.1\|c.69.1 (0.5953757) e.23.1 (0.1445087) -> e.23.1\|a.28.1\|c.43.1& (0.6820809): c.43.1&\|e.23.1\|a.28.1\|c.43.1&\|e.23.1\|a.28.1\|c.43.1&\|e.23.1\|a.28.1 (0.6763006) e.23.1 (0.1445087) -> a.28.1\|c.43.1&\|e.23.1\|a.28.1\|c.43.1&\|e.23.1\|a.28.1 (0.6820809): c.43.1&\|e.23.1\|a.28.1\|c.43.1&\|e.23.1\|a.28.1\|c.43.1&\|e.23.1\|a.28.1 (0.6763006) | 303 |
| **Sum total** | 217 | 560 | 777 |
| **8) Domain repeats and multidomains with no repeats in multidomains with repeats (720 /\ 535 = 486 parents with 486 common)** | | | |
|  | Arcs with part of repeat match | Arcs with complete match | Arcs Total |
| **Contemporaneous: Fusion** | 0 | 1 | 1 |
| **Fission** | 0 | 1 | 1 |
| **Fusion/Fission** | 0 | 0 | 0 |
| **Diachronic: Fusion** | 6 d.110.2\|d.110.3 (0.6763006) -> d.110.3& (0.6820809): c.23.1\|d.110.3&\|d.110.2\|d.110.3&\|d.110.2\|a.30.2\|d.122.1 (0.6820809) d.110.3\|d.110.2 (0.6763006) -> d.110.3& (0.6820809): c.23.1\|d.110.3&\|d.110.2\|d.110.3&\|d.110.2\|a.30.2\|d.122.1 (0.6820809) d.110.2\|d.110.3 (0.6763006) -> d.110.3& (0.6820809): c.23.1\|d.110.3&\|d.110.2\|d.110.3& (0.6878613) | 30 c.2.1\|d.81.1 (0.0867052) -> d.58.18& (0.3294798): c.73.1\|d.58.18&\|c.2.1\|d.81.1 (0.5202312) c.47.1\|a.45.1 (0.3179191) -> a.93.1& (0.5202312): c.47.1\|a.45.1\|a.93.1& (0.6763006) c.78.2& (0.3468208) -> c.66.1\|d.108.1 (0.6763006): c.66.1\|d.108.1\|c.78.2& (0.6763006) | 36 |
| **Fission** | 38 d.110.3& (0.6820809) -> c.23.1\|d.110.2 (0.7167630): c.23.1&\|d.110.2\|d.110.3&\|d.58.29\|c.1.33 (0.6763006) d.110.3& (0.6820809) -> a.30.2\|d.122.1\|c.23.1 (0.7225434): c.23.1\|d.110.2\|d.110.3&\|a.30.2\|d.122.1\|c.23.1&\|a.24.10 (0.6763006) d.110.2\|d.110.3 (0.6763006) -> c.23.1& (0.7283237): c.23.1&\|d.110.2\|d.110.3&\|d.58.29\|c.1.33 (0.6763006) | 152 d.110.2\|d.110.3 (0.6763006) -> d.110.3& (0.6820809): d.110.3&\|d.110.2\|d.110.3\|d.110.2\|a.30.2\|d.122.1\|c.23.1 (0.6705202) d.110.2\|d.110.3 (0.6763006) -> d.110.3& (0.6820809): d.110.3&\|d.110.2\|d.110.3\|a.30.2\|d.122.1 (0.6705202) d.110.3\|d.110.2 (0.6763006) -> d.110.3& (0.6820809): d.110.3\|d.110.2\|d.110.3&\|a.30.2\|d.122.1\|c.23.1 (0.6763006) | 190 |
| **Fusion/Fission** | 12 a.8.4& (0.6647399) -> c.55.1\|b.130.1 (0.7052023): c.55.1&\|b.130.1\|a.8.4& (0.6878613) c.55.1& (0.0462428) -> b.130.1\|a.8.4 (0.7052023): c.55.1&\|b.130.1\|a.8.4& (0.6878613) d.110.3& (0.6820809) -> a.30.2\|d.122.1\|c.23.1 (0.7225434): c.23.1\|d.110.3&\|a.30.2\|d.122.1\|c.23.1& (0.6878613) | 59 b.43.4\|c.25.1 (0.3352601) -> c.23.5& (0.6878613): c.23.5&\|b.43.4\|c.25.1 (0.6820809) d.142.2\|b.40.4\|a.60.2 (0.5895954) -> c.15.1& (0.6936416): d.142.2\|b.40.4\|a.60.2\|c.15.1& (0.6878613) b.40.4& (0.6358382) -> d.68.2\|c.37.1 (0.6936416): d.68.2\|c.37.1\|b.40.4& (0.6878613) | 71 |
| **Sum total** | 56 | 243 | 299 |
| **9) Domain repeats and multidomains with repeats in the latter (720 /\ 229 = 220 parents with 220 common)** | | | |
|  | Arcs with part of repeat match | Arcs with complete match | Arcs Total |
| **Contemporaneous: Fusion** | 0 | 0 | 0 |
| **Fission** | 0 | 5 | 5 |
| **Fusion/Fission** | 0 | 0 | 0 |
| **Diachronic: Fusion** | 0 | 3 c.23.1\|d.110.3&\|d.110.2 (0.6763006) -> d.110.3& (0.6820809): c.23.1\|d.110.3&\|d.110.2\|d.110.3&\|d.110.2\|a.30.2\|d.122.1 (0.6820809) c.23.1\|d.110.3&\|d.110.2 (0.6763006) -> d.110.3& (0.6820809): c.23.1\|d.110.3&\|d.110.2\|d.110.3& (0.6878613) c.26.2& (0.4682081) -> f.20.1\|d.37.1& (0.7052023): f.20.1\|d.37.1&\|c.26.2& (0.7225434) | 3 |
| **Fission** | 6 g.3.11\|b.29.1& (0.7109827) -> g.3.11& (0.7976879): g.3.11&\|b.29.1&\|g.3.11& (0.6647399) b.1.1\|b.1.2&\|b.1.1\|b.1.2 (0.6763006) -> b.1.2& (0.8728324): b.1.1\|b.1.2&\|b.1.1\|b.1.2&\|b.1.1\|b.1.2& (0.6589595) b.1.1\|b.1.2&\|b.1.1\|b.1.2 (0.6763006) -> b.1.2& (0.8728324): b.1.1\|b.1.2&\|b.1.1\|b.1.2&\|b.1.1\|b.1.2&\|b.1.1\|b.1.2&\|b.1.1\|b.1.2& (0.6763006) | 124 a.30.2\|d.122.1\|c.23.1&\|a.24.10 (0.6763006) -> d.110.3& (0.6820809): c.23.1\|d.110.2\|d.110.3&\|a.30.2\|d.122.1\|c.23.1&\|a.24.10 (0.6763006) d.110.3& (0.6820809) -> a.30.2\|d.122.1\|c.23.1& (0.6994219): c.23.1\|d.110.2\|d.110.3&\|a.30.2\|d.122.1\|c.23.1&\|a.24.10 (0.6763006) d.110.2\|d.110.3&\|d.58.29\|c.1.33 (0.6820809) -> c.23.1& (0.7283237): c.23.1&\|d.110.2\|d.110.3&\|d.58.29\|c.1.33 (0.6763006) | 130 |
| **Fusion/Fission** | 4 b.1.1\|b.1.2&\|b.1.1\|b.1.2 (0.6763006) -> b.1.2& (0.8728324): b.1.2&\|b.1.1\|b.1.2&\|b.1.1\|b.1.2&\|b.1.1\|b.1.2&\|b.1.1\|b.1.2& (0.6820809) b.1.1\|b.1.2&\|b.1.1\|b.1.2 (0.6763006) -> b.1.2& (0.8728324): b.1.2&\|b.1.1\|b.1.2&\|b.1.1\|b.1.2&\|b.1.1 (0.7109827) b.1.1\|b.1.2&\|b.1.1\|b.1.2 (0.6763006) -> b.1.2& (0.8728324): b.1.2\|b.1.1\|b.1.2&\|b.1.1\|b.1.2&\|b.1.1\|b.1.2&\|b.1.1\|b.1.2&\|b.1.1\|b.1.2&\|b.1.1\|b.1.2&\|b.1.1\|b.1.2&\|b.1.1\|b.1.2&\|b.1.1\|b.1.2&\|b.1.1\|b.1.2&\|b.1.1\|b.1.2&\|b.1.1\|b.1.2&\|b.1.1\|b.1.2&\|b.1.1\|b.1.2&\|b.1.1\|b.1.2&\|b.1.1\|b.1.2&\|b.1.1\|b.1.2&\|b.1.1\|b.1.2&\|b.1.1\|b.1.2& (0.7225434) | 55 c.37.1& (0.1907514) -> c.37.1&\|b.34.18 (0.6763006): c.37.1&\|b.34.18\|c.37.1&\|d.315.1 (0.4913295) c.37.1& (0.1907514) -> b.34.18\|c.37.1&\|d.315.1 (0.6936416): c.37.1&\|b.34.18\|c.37.1&\|d.315.1 (0.4913295) d.110.3& (0.6820809) -> d.110.2&\|a.30.2\|d.122.1 (0.6936416): d.110.2\|d.110.3&\|d.110.2&\|a.30.2\|d.122.1 (0.6878613) | 59 |
| **Sum total** | 10 | 187 | 197 |
| **10) Multidomains with no repeats and multidomains with repeats in the latter (535 /\ 229 = 215 parents with 215 common)** | | | |
|  | Arcs with part of repeat match | Arcs with complete match | Arcs Total |
| **Contemporaneous: Fusion** | 6 | 7 | 13 |
| **Fission** | 13 | 22 | 35 |
| **Fusion/Fission** | 2 | 1 | 3 |
| **Diachronic: Fusion** | 48 a.28.1\|c.43.1& (0.6763006) -> c.43.1\|e.23.1 (0.6820809): c.43.1&\|e.23.1\|a.28.1\|c.43.1&\|e.23.1\|a.28.1\|c.69.1 (0.6820809) b.1.2&\|b.1.1\|b.1.2&\|b.1.1 (0.6763006) -> b.1.2\|b.1.1 (0.6820809): b.1.2&\|b.1.1\|b.1.2&\|b.1.1\|b.1.2&\|b.1.1\|b.1.2&\|b.1.1\|b.1.2& (0.6820809) b.1.2&\|b.1.1\|b.1.2 (0.6763006) -> b.1.2\|b.1.1 (0.6820809): b.1.2&\|b.1.1\|b.1.2&\|b.1.1\|b.1.2&\|b.1.1\|b.1.2&\|b.1.1\|b.1.2& (0.6820809) | 45 c.2.1\|d.81.1 (0.0867052) -> c.73.1\|d.58.18& (0.3179191): c.73.1\|d.58.18&\|c.2.1\|d.81.1 (0.5202312) c.84.1&\|d.129.2 (0.1676301) -> c.68.1\|b.82.1 (0.6127167): c.84.1&\|d.129.2\|c.68.1\|b.82.1 (0.6763006) c.3.1\|d.87.1 (0.1560694) -> b.84.1&\|a.9.1\|c.43.1 (0.4739884): b.84.1&\|a.9.1\|c.43.1\|c.3.1\|d.87.1 (0.6763006) | 93 |
| **Fission** | 109 b.1.2&\|b.1.1\|b.1.2 (0.6763006) -> b.1.2\|b.1.1 (0.6820809): b.1.1\|b.1.2&\|b.1.1\|b.1.2&\|b.1.1\|b.1.2& (0.6589595) a.28.1\|c.43.1& (0.6763006) -> c.43.1\|e.23.1 (0.6820809): c.43.1&\|e.23.1\|a.28.1\|c.43.1&\|e.23.1\|a.28.1\|c.43.1&\|e.23.1\|a.28.1 (0.6763006) a.28.1\|c.43.1& (0.6763006) -> c.43.1\|e.23.1 (0.6820809): e.23.1\|a.28.1\|c.43.1&\|e.23.1\|a.28.1\|c.43.1&\|e.3.1 (0.6763006) | 133 c.43.1&\|e.23.1 (0.6763006) -> a.28.1\|c.69.1 (0.6820809): c.43.1&\|e.23.1\|a.28.1\|c.69.1 (0.5953757) a.28.1\|c.43.1& (0.6763006) -> e.23.1\|a.28.1\|c.69.1 (0.6820809): c.43.1&\|e.23.1\|a.28.1\|c.43.1&\|e.23.1\|a.28.1\|c.43.1&\|e.23.1\|a.28.1\|c.43.1&\|e.23.1\|a.28.1\|c.43.1&\|e.23.1\|a.28.1\|c.43.1&\|e.23.1\|a.28.1\|c.43.1&\|e.23.1\|a.28.1\|c.43.1&\|e.23.1\|a.28.1\|c.69.1 (0.6763006) e.23.1\|a.28.1\|c.43.1&\|e.23.1\|a.28.1\|c.43.1& (0.6763006) -> e.23.1\|a.28.1\|c.69.1 (0.6820809): c.43.1&\|e.23.1\|a.28.1\|c.43.1&\|e.23.1\|a.28.1\|c.43.1&\|e.23.1\|a.28.1\|c.43.1&\|e.23.1\|a.28.1\|c.43.1&\|e.23.1\|a.28.1\|c.43.1&\|e.23.1\|a.28.1\|c.43.1&\|e.23.1\|a.28.1\|c.43.1&\|e.23.1\|a.28.1\|c.69.1 (0.6763006) | 242 |
| **Fusion/Fission** | 20 c.43.1&\|e.23.1\|a.28.1\|c.69.1 (0.5953757) -> c.43.1\|e.23.1\|a.28.1 (0.6878613): c.43.1&\|e.23.1\|a.28.1\|c.43.1&\|e.23.1\|a.28.1\|c.43.1&\|e.23.1\|a.28.1\|c.43.1&\|e.23.1\|a.28.1\|c.43.1&\|e.23.1\|a.28.1\|c.43.1&\|e.23.1\|a.28.1\|c.43.1&\|e.23.1\|a.28.1\|c.43.1&\|e.23.1\|a.28.1\|c.69.1 (0.6763006) c.43.1&\|e.23.1\|a.28.1\|c.69.1 (0.5953757) -> c.43.1\|e.23.1\|a.28.1 (0.6878613): c.43.1&\|e.23.1\|a.28.1\|c.43.1&\|e.23.1\|a.28.1\|c.43.1&\|e.23.1\|a.28.1\|c.43.1&\|e.23.1\|a.28.1\|c.43.1&\|e.23.1\|a.28.1\|c.43.1&\|e.23.1\|a.28.1\|c.43.1&\|e.23.1\|a.28.1\|c.43.1&\|e.23.1\|a.28.1\|c.43.1&\|e.23.1\|a.28.1\|c.69.1 (0.6763006) c.43.1&\|e.23.1\|a.28.1\|c.69.1 (0.5953757) -> c.43.1\|e.23.1\|a.28.1 (0.6878613): c.43.1&\|e.23.1\|a.28.1\|c.43.1&\|e.23.1\|a.28.1\|c.43.1&\|e.23.1\|a.28.1\|c.43.1&\|e.23.1\|a.28.1\|c.43.1&\|e.23.1\|a.28.1\|c.69.1 (0.6763006) | 48 c.30.1\|d.142.1\|b.84.2 (0.3526012) -> b.84.1\|c.14.1& (0.6994219): c.30.1\|d.142.1\|b.84.2\|b.84.1\|c.14.1& (0.6878613) d.129.1\|a.96.1 (0.6011561) -> g.48.1\|a.4.1& (0.6994219): g.48.1\|a.4.1&\|d.129.1\|a.96.1 (0.6936416) d.110.3\|a.30.2 (0.6763006) -> d.110.3&\|d.58.29\|c.1.33 (0.7109827): c.23.1\|d.110.3\|a.30.2\|d.110.3&\|d.58.29\|c.1.33 (0.6820809) | 68 |
| **Sum total** | 198 | 256 | 454 |
| **Grand total** | 1,991 | 10,420 | 12,411 |
| **11) Domain Categories** | | | |
| **Donors** | 625 - c.37.1 (0.0000000); e.7.1 (0.1329480); a.128.1 (0.1387283) | |  |
| **Acceptors** | 611 - c.36.1&\|c.48.1 (0.1849711); c.56.2 (0.1907514); d.14.1\|d.58.26 (0.1907514) | | |
| **Donors/Acceptors** | 909 - c.2.1 (0.0057803); c.67.1 (0.0115607); c.66.1 (0.0173410) | |  |
| **Peers** | 22 - c.114.1 (0.3583815); f.25.1 (0.3641618); d.52.4 (0.6763006) | |  |
| **Noncombinables** | 3,995 - d.41.2\|c.1.17 (0.1734104); c.37.1\|b.43.3\|d.58.11\|d.14.1\|d.58.11 (0.1791908); g.41.8 (0.1965318) | | |

**Supplementary Table 4: Cumulative final counts per categories of nodes (domains) and connecting arcs in SPX at age *nd* = 1.** The spliced pairwise network (SPX) is a partial subset of PX. Its nodes-set comprises of 1,643 single and spliced domains. In this smaller data set, 1,513 were single domains. The age assignments from the original data set of domain organization were retained for these single domains. The rest of the 130 domains were spliced domains extracted from the domain repeats (5), multidomains with no repeats (116) and multidomains with repeats (9) subgroups, in that order. The domains extracted from these structural subgroups were unique across the subgroups, in the order defined above. In case of multiple occurrences of a domain in parent multidomains within a subgroup, a spliced domain was assigned the age (and other attributes) of the most ancient multidomain it was spliced from. Given the above defined order of structural groups for extraction of the SPX domains-set and the scheme for assignment of ages, eight of the 169 unique time events of the original 6,162 domains, supradomains and multidomains data set were left unassigned in the SPX 1,643 domains data set. SPX defines connectivity among the single and spliced domains as pairwise events in the same or other proteins, a criterion similar to that of PX. It was noticed that the structural subgroup of multidomains with repeats was fourth and last in the order of extraction of unique spliced domains. Also, these domains were mutually exclusive to the unique spliced domains extracted until the third subgroup of multidomains with no repeats. Therefore, no pairwise events of domains from the fourth group were expected in the parent multidomains of the third group. The algorithm designed to generate the domain pairs had a time complexity that was reduced to O((n^2-n)/2) from O(n^3) by pre-generating parent multidomain arrays and utilizing set intersection function. The space complexity of the algorithm was <= 128MB and the run time was ~18 seconds. A parent node was defined as a multidomain that was composed by at least two single or spliced child domains through a complete or a part of repeat match, with priority given to the complete match type. Also, both the child single or spliced domains of the pair needed to match completely in the parent multidomain to specify a complete match of the pair. However, a part of repeat match of a pair could be specified by part of repeat match of any child. A fusion event was specified when a parent multidomain was younger than or of the same age as the younger child in the single or spliced domain pair. Similarly, a fission event was specified when a parent multidomain was older than or of the same age as the older child in the single or spliced domain pair. However, a fusion/fission event was specified when a parent multidomain's age was between the ages of the two single or spliced child domains in the pair, possibly equal to any child node’s age. Self- pairwise events due to multiple appearances of a single or spliced child domain in a parent multidomain were not included as arcs because such duplications were considered as a single appearance in the timeline. Similarly, arcs that represented self-pairwise links of single or spliced domains in domain repeats were considered redundant and hence excluded. Since the data set was exclusive of any supradomains, no overlaps could be considered.

| Self-set comparisons | | | |
| --- | --- | --- | --- |
| 1a) Single domains in multidomains with no repeats (3,483 /\ 3,483 = 3,483 parents with 3,364 common) | | | |
|  | Arcs with part of repeat match | Arcs with complete match | Arcs Total |
| Contemporaneous: Fusion | 0 | 41 | 41 |
| Fission | 0 | 113 | 113 |
| Fusion/Fission | 0 | 4 | 4 |
| Diachronic: Fusion | 0 | 1,583  c.37.1 (0.0000000) -> d.14.1 (0.1791908): c.37.1\|b.43.3\|d.58.11\|d.14.1\|d.58.11 (0.1791908)  c.37.1 (0.0000000) -> c.23.16 (0.0809249): c.37.1\|c.23.16 (0.2369942)  c.37.1 (0.0000000) -> a.4.5 (0.0751445): c.37.1\|a.4.5 (0.2716763) | 1,583 |
| Fission | 0 | 1,928  c.31.1 (0.2543353) -> c.36.1 (0.2601156): c.36.1\|c.31.1\|c.36.1 (0.2023121)  d.14.1 (0.1791908) -> b.43.3 (0.2890173): c.37.1\|b.43.3\|d.58.11\|d.14.1\|d.58.11 (0.1791908)  c.116.1 (0.3526012) -> b.122.1 (0.3641618): b.122.1\|c.116.1 (0.3294798) | 1,928 |
| Fusion/Fission | 0 | 2,113  c.37.1 (0.0000000) -> b.43.3 (0.2890173): c.37.1\|b.43.3\|d.58.11\|d.14.1\|d.58.11 (0.1791908)  c.37.1 (0.0000000) -> b.43.3 (0.2890173): c.37.1\|b.43.3\|b.44.1 (0.1965318)  c.37.1 (0.0000000) -> g.39.1 (0.3468208): g.39.1\|c.37.1 (0.3121387) | 2,113 |
| Sum total | 0 | 5,782 | 5,782 |
| 1b) Single domains in multidomains with repeats (777 /\ 777 = 777 parents with 765 common) | | | |
|  | Arcs with part of repeat match | Arcs with complete match | Arcs Total |
| Contemporaneous: Fusion | 4 | 2 | 6 |
| Fission | 30 | 13 | 43 |
| Fusion/Fission | 0 | 1 | 1 |
| Diachronic: Fusion | 158  b.40.4 (0.0520231) -> b.34.5 (0.1849711): b.34.5\|b.40.4& (0.3005780)  c.55.1 (0.1907514) -> a.211.1 (0.2543353): c.55.1&\|a.211.1 (0.4450867)  c.47.1 (0.0231214) -> c.3.1 (0.1849711): c.47.1&\|c.3.1 (0.4508671) | 108  c.2.1 (0.0057803) -> c.73.1 (0.3641618): c.73.1\|d.58.18&\|c.2.1\|d.81.1 (0.5202312)  c.69.1 (0.0346821) -> e.23.1 (0.1445087): c.43.1&\|e.23.1\|a.28.1\|c.69.1 (0.5953757)  c.69.1 (0.0346821) -> a.28.1 (0.3526012): c.43.1&\|e.23.1\|a.28.1\|c.69.1 (0.5953757) | 266 |
| Fission | 493  a.4.1 (0.6647399) -> b.82.4 (0.6878613): b.82.4\|a.4.1& (0.5549133)  b.1.3 (0.6820809) -> c.6.1 (0.6878613): b.2.2\|b.1.3&\|c.6.1 (0.6820809)  a.118.14 (0.6820809) -> d.37.1 (0.6936416): a.118.14\|d.37.1& (0.6763006) | 241  c.142.1 (0.6763006) -> d.15.13 (0.6820809): c.47.1&\|c.142.1\|d.15.13\|a.29.12 (0.6763006)  a.29.12 (0.6763006) -> d.15.13 (0.6820809): c.47.1&\|c.142.1\|d.15.13\|a.29.12 (0.6763006)  d.142.2 (0.6878613) -> a.60.2 (0.6936416): d.142.2\|b.40.4\|a.60.2\|c.15.1& (0.6878613) | 734 |
| Fusion/Fission | 392  c.37.1 (0.0000000) -> d.315.1 (0.6763006): c.37.1&\|b.34.18\|c.37.1&\|d.315.1 (0.4913295)  c.55.1 (0.1907514) -> d.22.1 (0.6763006): c.55.1&\|d.22.1 (0.6647399)  b.40.4 (0.0520231) -> a.60.4 (0.6820809): d.202.1\|b.40.4\|d.52.3&\|a.60.4& (0.4219653) | 198  c.23.1 (0.4046243) -> a.30.2 (0.6820809): d.110.3&\|d.110.2\|d.110.3\|d.110.2\|a.30.2\|d.122.1\|c.23.1 (0.6705202)  c.23.1 (0.4046243) -> a.30.2 (0.6820809): d.110.3\|d.110.2&\|a.30.2\|d.122.1\|c.23.1 (0.6763006)  c.23.1 (0.4046243) -> a.30.2 (0.6820809): d.110.2\|d.110.3&\|a.30.2\|d.122.1\|c.23.1 (0.6763006) | 590 |
| Sum total | 1,077 | 563 | 1,640 |
| 2a) Domains from domain repeats in multidomains with no repeats (4 /\ 4 = 4 parents with 0 common) | | | |
|  | Arcs with part of repeat match | Arcs with complete match | Arcs Total |
| Contemporaneous: Fusion | 0 | 0 | 0 |
| Fission | 0 | 0 | 0 |
| Fusion/Fission | 0 | 0 | 0 |
| Diachronic: Fusion | 0 | 0 | 0 |
| Fission | 0 | 0 | 0 |
| Fusion/Fission | 0 | 0 | 0 |
| Sum total | 0 | 0 | 0 |
| 2b) Domains from domain repeats in multidomains with repeats (3 /\ 3 = 3 parents with 0 common) | | | |
|  | Arcs with part of repeat match | Arcs with complete match | Arcs Total |
| Contemporaneous: Fusion | 0 | 0 | 0 |
| Fission | 0 | 0 | 0 |
| Fusion/Fission | 0 | 0 | 0 |
| Diachronic: Fusion | 0 | 0 | 0 |
| Fission | 0 | 0 | 0 |
| Fusion/Fission | 0 | 0 | 0 |
| Sum total | 0 | 0 | 0 |
| 3a) Domains from multidomains with no repeats in multidomains with no repeats (248 /\ 248 = 248 parents with 3 common) | | | |
|  | Arcs with part of repeat match | Arcs with complete match | Arcs Total |
| Contemporaneous: Fusion | 0 | 0 | 0 |
| Fission | 0 | 0 | 0 |
| Fusion/Fission | 0 | 3 | 3 |
| Diachronic: Fusion | 0 | 0 | 0 |
| Fission | 0 | 0 | 0 |
| Fusion/Fission | 0 | 0 | 0 |
| Sum total | 0 | 3 | 3 |
| 3b) Domains from multidomains with no repeats in multidomains with repeats (16 /\ 16 = 16 parents with 0 common) | | | |
|  | Arcs with part of repeat match | Arcs with complete match | Arcs Total |
| Contemporaneous: Fusion | 0 | 0 | 0 |
| Fission | 0 | 0 | 0 |
| Fusion/Fission | 0 | 0 | 0 |
| Diachronic: Fusion | 0 | 0 | 0 |
| Fission | 0 | 0 | 0 |
| Fusion/Fission | 0 | 0 | 0 |
| Sum total | 0 | 0 | 0 |
| 4) Domains from multidomains with repeats in multidomains with repeats (13 /\ 13 = 13 parents with 1 common) | | | |
|  | Arcs with part of repeat match | Arcs with complete match | Arcs Total |
| Contemporaneous: Fusion | 0 | 0 | 0 |
| Fission | 0 | 0 | 0 |
| Fusion/Fission | 1 | 0 | 1 |
| Diachronic: Fusion | 0 | 0 | 0 |
| Fission | 0 | 0 | 0 |
| Fusion/Fission | 0 | 0 | 0 |
| Sum total | 1 | 0 | 1 |
| Paired set comparisons | | | |
| 5a) Single domains and domains from domain repeats in multidomains with no repeats (3,483 /\ 4 = 4 parents with 4 common) | | | |
|  | Arcs with part of repeat match | Arcs with complete match | Arcs Total |
| Contemporaneous: Fusion | 0 | 0 | 0 |
| Fission | 0 | 0 | 0 |
| Fusion/Fission | 0 | 0 | 0 |
| Diachronic: Fusion | 0 | 2  a.118.19 (0.6763006) -> b.131.1 (0.7630058): c.62.1\|b.131.1\|a.118.19 (0.8265896)  a.118.19 (0.6763006) -> c.62.1 (0.7976879): c.62.1\|b.131.1\|a.118.19 (0.8265896) | 2 |
| Fission | 0 | 1  g.41.11 (0.8092486) -> d.58.7 (0.9306358): g.41.11\|d.58.7 (0.6763006) | 1 |
| Fusion/Fission | 0 | 2  a.118.19 (0.6763006) -> b.131.1 (0.7630058): b.131.1\|a.118.19 (0.7109827)  d.223.1 (0.6820809) -> d.144.1 (0.9710982): d.144.1\|d.223.1 (0.6936416) | 2 |
| Sum total | 0 | 5 | 5 |
| 5b) Single domains and domains from domain repeats in multidomains with repeats (777 /\ 3 = 3 parents with 3 common) | | | |
|  | Arcs with part of repeat match | Arcs with complete match | Arcs Total |
| Contemporaneous: Fusion | 0 | 0 | 0 |
| Fission | 0 | 0 | 0 |
| Fusion/Fission | 0 | 0 | 0 |
| Diachronic: Fusion | 1  b.23.2 (0.6820809) -> d.82.1 (0.7283237): b.23.2&\|d.82.1 (0.7283237) | 0 | 1 |
| Fission | 1  b.23.2 (0.6820809) -> a.86.1 (0.7630058): a.86.1\|b.23.2& (0.6763006) | 0 | 1 |
| Fusion/Fission | 1  d.223.1 (0.6820809) -> d.144.1 (0.9710982): d.144.1\|d.223.1& (0.8439307) | 0 | 1 |
| Sum total | 3 | 0 | 3 |
| 6a) Single domains and domains from multidomains with no repeats in multidomains with no repeats (3,483 /\ 248 = 247 parents with 247 common) | | | |
|  | Arcs with part of repeat match | Arcs with complete match | Arcs Total |
| Contemporaneous: Fusion | 0 | 6 | 6 |
| Fission | 0 | 0 | 0 |
| Fusion/Fission | 0 | 4 | 4 |
| Diachronic: Fusion | 0 | 211  c.37.1 (0.0000000) -> a.36.1 (0.2716763): a.24.13\|c.37.1\|a.36.1 (0.2716763)  b.40.4 (0.0520231) -> a.5.1 (0.3236994): b.40.4\|a.60.2\|a.5.1 (0.3236994)  c.2.1 (0.0057803) -> a.69.3 (0.3294798): c.2.1\|d.81.1\|a.69.3 (0.3294798) | 211 |
| Fission | 0 | 116  b.105.1 (0.3815029) -> e.3.1 (0.3872832): e.3.1\|b.105.1 (0.3815029) a.108.1 (0.3352601) -> d.45.1 (0.4624277): a.108.1\|d.45.1 (0.3352601)  a.92.1 (0.3121387) -> c.24.1 (0.5491329): c.30.1\|d.142.1\|a.92.1\|c.30.1\|d.142.1\|c.24.1 (0.3121387) | 116 |
| Fusion/Fission | 0 | 139  g.81.1 (0.3641618) -> d.193.1 (0.6820809): d.193.1\|g.81.1\|c.51.5 (0.6763006)  c.98.1 (0.3294798) -> c.59.1 (0.6820809): c.108.1\|c.98.1\|c.72.2\|c.59.1 (0.6763006)  a.5.10 (0.4219653) -> d.79.4 (0.6878613): d.284.1\|a.5.10\|d.79.4\|d.139.1 (0.6705202) | 139 |
| Sum total | 0 | 476 | 476 |
| 6b) Single domains and domains from multidomains with no repeats in multidomains with repeats (777 /\ 16 = 16 parents with 16 common) | | | |
|  | Arcs with part of repeat match | Arcs with complete match | Arcs Total |
| Contemporaneous: Fusion | 0 | 0 | 0 |
| Fission | 0 | 0 | 0 |
| Fusion/Fission | 0 | 0 | 0 |
| Diachronic: Fusion | 4  c.2.1 (0.0057803) -> d.58.39 (0.4450867): d.58.39\|c.2.1& (0.6763006)  h.1.7 (0.6763006) -> g.75.1 (0.6820809): b.29.1\|h.1.7\|g.3.11&\|g.75.1&\|b.29.1 (0.6878613)  c.37.1 (0.0000000) -> a.2.9 (0.6878613): c.37.1&\|a.2.9 (0.6994219) | 5  c.3.1 (0.1849711) -> a.9.1 (0.3930636): b.84.1&\|a.9.1\|c.43.1\|c.3.1\|d.87.1 (0.6763006)  c.23.16 (0.0809249) -> a.5.10 (0.4219653): d.284.1\|a.5.10\|d.79.4&\|d.139.1\|c.23.16 (0.6820809)  e.3.1 (0.3872832) -> d.175.1 (0.6936416): d.175.1\|e.3.1\|d.11.1& (0.7052023) | 9 |
| Fission | 2  h.1.6 (0.6820809) -> g.3.11 (0.8034682): c.62.1\|g.3.11&\|h.1.6 (0.6763006)  h.1.6 (0.6820809) -> g.3.11 (0.8034682): c.62.1\|g.3.11&\|c.62.1\|h.1.6 (0.6820809) | 2  h.1.6 (0.6820809) -> c.62.1 (0.7976879): c.62.1\|g.3.11&\|h.1.6 (0.6763006)  h.1.6 (0.6820809) -> c.62.1 (0.7976879): c.62.1\|g.3.11&\|c.62.1\|h.1.6 (0.6820809) | 4 |
| Fusion/Fission | 11  a.4.1 (0.6647399) -> b.48.1 (0.6763006): a.4.1&\|c.55.3\|b.48.1 (0.6705202)  a.5.10 (0.4219653) -> d.79.4 (0.6878613): d.284.1\|a.5.10\|d.79.4&\|d.139.1\|c.23.16 (0.6820809)  a.9.1 (0.3930636) -> c.43.1 (0.7398844): b.84.1\|a.9.1&\|c.43.1 (0.6820809) | 8  c.55.3 (0.0809249) -> b.48.1 (0.6763006): a.4.1&\|c.55.3\|b.48.1 (0.6705202)  a.9.1 (0.3930636) -> d.87.1 (0.7052023): b.84.1&\|a.9.1\|c.43.1\|c.3.1\|d.87.1 (0.6763006)  b.149.1 (0.6763006) -> b.71.1 (0.7052023): b.71.1\|b.149.1\|b.18.1& (0.6994219) | 19 |
| Sum total | 17 | 15 | 32 |
| 7) Single domains and domains from multidomains with repeats in multidomains with repeats (777 /\ 13 = 12 parents with 12 common) | | | |
|  | Arcs with part of repeat match | Arcs with complete match | Arcs Total |
| Contemporaneous: Fusion | 0 | 0 | 0 |
| Fission | 0 | 0 | 0 |
| Fusion/Fission | 0 | 0 | 0 |
| Diachronic: Fusion | 6  c.37.1 (0.0000000) -> b.34.18 (0.4913295): c.37.1&\|b.34.18\|c.37.1&\|d.315.1 (0.4913295)  c.37.1 (0.0000000) -> b.34.18 (0.4913295): c.37.1&\|b.34.18 (0.6763006)  a.3.1 (0.2890173) -> b.61.4 (0.6878613): a.3.1&\|b.61.4\|b.1.18& (0.6878613) | 7  b.40.4 (0.0520231) -> d.202.1 (0.4219653): d.202.1\|b.40.4\|d.52.3&\|a.60.4& (0.4219653)  b.34.18 (0.4913295) -> d.315.1 (0.6763006): b.34.18\|c.37.1&\|d.315.1 (0.6936416)  c.37.1 (0.0000000) -> b.34.18 (0.4913295): c.37.1\|b.34.18\|c.37.1&\|d.315.1 (0.6936416) | 13 |
| Fission | 5  d.202.1 (0.4219653) -> a.60.4 (0.6820809): d.202.1\|b.40.4\|d.52.3&\|a.60.4& (0.4219653)  g.41.13 (0.6820809) -> d.37.1 (0.6936416): d.37.1&\|g.41.13 (0.6820809)  a.7.15 (0.4450867) -> d.136.1 (0.7167630): a.7.15\|d.322.1\|d.136.1& (0.4450867) | 2  b.34.18 (0.4913295) -> d.315.1 (0.6763006): c.37.1&\|b.34.18\|c.37.1&\|d.315.1 (0.4913295)  a.7.15 (0.4450867) -> d.322.1 (0.8092486): a.7.15\|d.322.1\|d.136.1& (0.4450867) | 7 |
| Fusion/Fission | 2  d.202.1 (0.4219653) -> d.52.3 (0.7745665): d.202.1\|b.40.4\|d.52.3&\|a.60.4 (0.7167630)  d.202.1 (0.4219653) -> d.52.3 (0.7745665): d.202.1\|b.40.4\|d.52.3& (0.7687861) | 0 | 2 |
| Sum total | 13 | 9 | 22 |
| 8a) Domains from both domain repeats and multidomains with no repeats in multidomains with no repeats (4 /\ 248 = 0 parents with 0 common) | | | |
|  | Arcs with part of repeat match | Arcs with complete match | Arcs Total |
| Contemporaneous: Fusion | 0 | 0 | 0 |
| Fission | 0 | 0 | 0 |
| Fusion/Fission | 0 | 0 | 0 |
| Diachronic: Fusion | 0 | 0 | 0 |
| Fission | 0 | 0 | 0 |
| Fusion/Fission | 0 | 0 | 0 |
| Sum total | 0 | 0 | 0 |
| 8b) Domains from both domain repeats and multidomains with no repeats in multidomains with repeats (3 /\ 16 = 0 parents with 0 common) | | | |
|  | Arcs with part of repeat match | Arcs with complete match | Arcs Total |
| Contemporaneous: Fusion | 0 | 0 | 0 |
| Fission | 0 | 0 | 0 |
| Fusion/Fission | 0 | 0 | 0 |
| Diachronic: Fusion | 0 | 0 | 0 |
| Fission | 0 | 0 | 0 |
| Fusion/Fission | 0 | 0 | 0 |
| Sum total | 0 | 0 | 0 |
| 9) Domains from both domain repeats and multidomains with repeats in multidomains with repeats (3 /\ 13 = 0 parents with 0 common) | | | |
|  | Arcs with part of repeat match | Arcs with complete match | Arcs Total |
| Contemporaneous: Fusion | 0 | 0 | 0 |
| Fission | 0 | 0 | 0 |
| Fusion/Fission | 0 | 0 | 0 |
| Diachronic: Fusion | 0 | 0 | 0 |
| Fission | 0 | 0 | 0 |
| Fusion/Fission | 0 | 0 | 0 |
| Sum total | 0 | 0 | 0 |
| 10) Domains from both multidomains with no repeats and multidomains with repeats in multidomains with repeats (16 /\ 13 = 1 parents with 1 common) | | | |
|  | Arcs with part of repeat match | Arcs with complete match | Arcs Total |
| Contemporaneous: Fusion | 0 | 0 | 0 |
| Fission | 0 | 0 | 0 |
| Fusion/Fission | 0 | 0 | 0 |
| Diachronic: Fusion | 1  d.175.1 (0.6936416) -> d.11.1 (0.7052023): d.175.1\|e.3.1\|d.11.1& (0.7052023) | 0 | 1 |
| Fission | 0 | 0 | 0 |
| Fusion/Fission | 0 | 0 | 0 |
| Sum total | 1 | 0 | 1 |
| Grand total | 1,112 | 6,853 | 7,965 |
| 11) Domain Categories | | | |
| Donors | 232 - c.37.1 (0.0000000); e.7.1 (0.1329480); a.128.1 (0.1387283) | | |
| Acceptors | 331 - c.56.2 (0.1907514); c.12.1 (0.2312139); c.18.1 (0.2369942) | | |
| Donors/Acceptors | 566 - c.2.1 (0.0057803); c.67.1 (0.0115607); c.66.1 (0.0173410) | | |
| Peers | 18 - c.114.1 (0.3583815); f.25.1 (0.3641618); d.52.4 (0.6763006) | | |
| Noncombinables | 496 - g.41.8 (0.1965318); f.41.1 (0.2312139); a.75.1 (0.2427746) | | |

**Supplementary Table 5: Cumulative final counts per categories of nodes (domains) and connecting arcs in SPAX at age *nd* = 1.** The spliced pairwise adjacency network (SPAX) is a subset of SPX and a partial subset of PAX. Its nodes-set also comprises of 1,643 single and spliced domains, in accordance to SPX. Given the predefined order of structural groups for extraction of the SPX/SPAX domains-set and the scheme for assignment of ages, eight of the 169 unique time events of the original 6,162 domains, supradomains and multidomains data set were left unassigned in the SPX/SPAX 1,643 domains data set. SPAX defines connectivity among the single and spliced domains as contiguously adjacent pairwise events in the same or other proteins. These pairwise events were not differentiated based on a left-to-right or right-to-left permutation. SPAX connectivity criterion intersects those of SPX and PAX. The algorithm designed to generate the adjacent domain pairs had the same time complexity (and execution time) as that of SPX because adjacency constraint was placed beyond the step that determined the parent multidomain arrays. The space complexity of the algorithm, however, was reduced to <= 110MB. Definitions of a parent multidomain and of the fusion, fission and fusion/fission events were borrowed from SPX. Also, possible self-pairs that represented adjacent recurrence of a domain in a parent multidomain or a domain repeat were not included. In order to determine a part of repeat match, the contiguous domain pair was considered as a whole, instead of individual child nodes. This ensured the contiguity of the domain pair because the domains at the carboxyl or amino end of the whole pair (permuted either of the two ways) occurred in a parent multidomain with repeats for a part of repeat match. However, if available, priority was given to the complete match type. Notably, there were no possible overlaps defined in the connectivity between any adjacent domains.

| Self-set comparisons | | | |
| --- | --- | --- | --- |
| 1a) Single domains in multidomains with no repeats (3,483 /\ 3,483 = 3,483 parents with 3,364 common) | | | |
|  | Arcs with part of repeat match | Arcs with complete match | Arcs Total |
| Contemporaneous: Fusion | 0 | 30 | 30 |
| Fission | 0 | 94 | 94 |
| Fusion/Fission | 0 | 3 | 3 |
| Diachronic: Fusion | 0 | 1,230 c.37.1 (0.0000000) -> c.23.16 (0.0809249): c.37.1\|c.23.16 (0.2369942) c.37.1 (0.0000000) -> a.4.5 (0.0751445): c.37.1\|a.4.5 (0.2716763) d.153.1 (0.1445087) -> c.80.1 (0.1618497): d.153.1\|c.80.1 (0.3005780) | 1,230 |
| Fission | 0 | 1,438 c.31.1 (0.2543353) -> c.36.1 (0.2601156): c.36.1\|c.31.1\|c.36.1 (0.2023121) c.116.1 (0.3526012) -> b.122.1 (0.3641618): b.122.1\|c.116.1 (0.3294798) d.307.1 (0.6531792) -> e.18.1 (0.6589595): d.307.1\|e.18.1 (0.5895954) | 1,438 |
| Fusion/Fission | 0 | 1,570 c.37.1 (0.0000000) -> b.43.3 (0.2890173): c.37.1\|b.43.3\|d.58.11\|d.14.1\|d.58.11 (0.1791908) c.37.1 (0.0000000) -> b.43.3 (0.2890173): c.37.1\|b.43.3\|b.44.1 (0.1965318) c.37.1 (0.0000000) -> g.39.1 (0.3468208): g.39.1\|c.37.1 (0.3121387) | 1,570 |
| Sum total | 0 | 4,365 | 4,365 |
| 1b) Single domains in multidomains with repeats (777 /\ 777 = 777 parents with 765 common) | | | |
|  | Arcs with part of repeat match | Arcs with complete match | Arcs Total |
| Contemporaneous: Fusion | 3 | 2 | 5 |
| Fission | 27 | 9 | 36 |
| Fusion/Fission | 0 | 0 | 0 |
| Diachronic: Fusion | 118 b.40.4 (0.0520231) -> b.34.5 (0.1849711): b.34.5\|b.40.4& (0.3005780) c.55.1 (0.1907514) -> a.211.1 (0.2543353): c.55.1&\|a.211.1 (0.4450867) c.47.1 (0.0231214) -> c.3.1 (0.1849711): c.47.1&\|c.3.1 (0.4508671) | 59 c.69.1 (0.0346821) -> a.28.1 (0.3526012): c.43.1&\|e.23.1\|a.28.1\|c.69.1 (0.5953757) e.23.1 (0.1445087) -> a.28.1 (0.3526012): c.43.1&\|e.23.1\|a.28.1\|c.69.1 (0.5953757) c.2.1 (0.0057803) -> c.14.1 (0.1387283): c.14.1\|c.2.1\|a.100.1& (0.6416185) | 177 |
| Fission | 385 a.4.1 (0.6647399) -> b.82.4 (0.6878613): b.82.4\|a.4.1& (0.5549133) b.1.3 (0.6820809) -> c.6.1 (0.6878613): b.2.2\|b.1.3&\|c.6.1 (0.6820809) a.118.14 (0.6820809) -> d.37.1 (0.6936416): a.118.14\|d.37.1& (0.6763006) | 129 c.142.1 (0.6763006) -> d.15.13 (0.6820809): c.47.1&\|c.142.1\|d.15.13\|a.29.12 (0.6763006) a.29.12 (0.6763006) -> d.15.13 (0.6820809): c.47.1&\|c.142.1\|d.15.13\|a.29.12 (0.6763006) a.102.1 (0.6936416) -> b.2.2 (0.6994219): a.102.1\|b.2.2\|b.1.18&\|b.2.2 (0.6763006) | 514 |
| Fusion/Fission | 296 c.37.1 (0.0000000) -> d.315.1 (0.6763006): c.37.1&\|b.34.18\|c.37.1&\|d.315.1 (0.4913295) c.55.1 (0.1907514) -> d.22.1 (0.6763006): c.55.1&\|d.22.1 (0.6647399) c.37.1 (0.0000000) -> a.60.4 (0.6820809): a.60.4&\|c.37.1 (0.6763006) | 106 b.40.4 (0.0520231) -> a.171.1 (0.6820809): a.170.1\|b.40.4\|a.171.1\|b.40.4& (0.6763006) b.40.4 (0.0520231) -> a.170.1 (0.6820809): a.170.1\|b.40.4\|a.171.1\|b.40.4& (0.6763006) c.68.1 (0.0693642) -> d.129.2 (0.6878613): c.84.1&\|d.129.2\|c.68.1\|b.82.1 (0.6763006) | 402 |
| Sum total | 829 | 305 | 1,134 |
| 2a) Domains from domain repeats in multidomains with no repeats (4 /\ 4 = 4 parents with 0 common) | | | |
|  | Arcs with part of repeat match | Arcs with complete match | Arcs Total |
| Contemporaneous: Fusion | 0 | 0 | 0 |
| Fission | 0 | 0 | 0 |
| Fusion/Fission | 0 | 0 | 0 |
| Diachronic: Fusion | 0 | 0 | 0 |
| Fission | 0 | 0 | 0 |
| Fusion/Fission | 0 | 0 | 0 |
| Sum total | 0 | 0 | 0 |
| 2b) Domains from domain repeats in multidomains with repeats (3 /\ 3 = 3 parents with 0 common) | | | |
|  | Arcs with part of repeat match | Arcs with complete match | Arcs Total |
| Contemporaneous: Fusion | 0 | 0 | 0 |
| Fission | 0 | 0 | 0 |
| Fusion/Fission | 0 | 0 | 0 |
| Diachronic: Fusion | 0 | 0 | 0 |
| Fission | 0 | 0 | 0 |
| Fusion/Fission | 0 | 0 | 0 |
| Sum total | 0 | 0 | 0 |
| 3a) Domains from multidomains with no repeats in multidomains with no repeats (248 /\ 248 = 248 parents with 3 common) | | | |
|  | Arcs with part of repeat match | Arcs with complete match | Arcs Total |
| Contemporaneous: Fusion | 0 | 0 | 0 |
| Fission | 0 | 0 | 0 |
| Fusion/Fission | 0 | 2 | 2 |
| Diachronic: Fusion | 0 | 0 | 0 |
| Fission | 0 | 0 | 0 |
| Fusion/Fission | 0 | 0 | 0 |
| Sum total | 0 | 2 | 2 |
| 3b) Domains from multidomains with no repeats in multidomains with repeats (16 /\ 16 = 16 parents with 0 common) | | | |
|  | Arcs with part of repeat match | Arcs with complete match | Arcs Total |
| Contemporaneous: Fusion | 0 | 0 | 0 |
| Fission | 0 | 0 | 0 |
| Fusion/Fission | 0 | 0 | 0 |
| Diachronic: Fusion | 0 | 0 | 0 |
| Fission | 0 | 0 | 0 |
| Fusion/Fission | 0 | 0 | 0 |
| Sum total | 0 | 0 | 0 |
| 4) Domains from multidomains with repeats in multidomains with repeats (13 /\ 13 = 13 parents with 1 common) | | | |
|  | Arcs with part of repeat match | Arcs with complete match | Arcs Total |
| Contemporaneous: Fusion | 0 | 0 | 0 |
| Fission | 0 | 0 | 0 |
| Fusion/Fission | 1 | 0 | 1 |
| Diachronic: Fusion | 0 | 0 | 0 |
| Fission | 0 | 0 | 0 |
| Fusion/Fission | 0 | 0 | 0 |
| Sum total | 1 | 0 | 1 |
| Paired set comparisons | | | |
| 5a) Single domains and domains from domain repeats in multidomains with no repeats (3,483 /\ 4 = 4 parents with 4 common) | | | |
|  | Arcs with part of repeat match | Arcs with complete match | Arcs Total |
| Contemporaneous: Fusion | 0 | 0 | 0 |
| Fission | 0 | 0 | 0 |
| Fusion/Fission | 0 | 0 | 0 |
| Diachronic: Fusion | 0 | 1 a.118.19 (0.6763006) -> b.131.1 (0.7630058): c.62.1\|b.131.1\|a.118.19 (0.8265896) | 1 |
| Fission | 0 | 1 g.41.11 (0.8092486) -> d.58.7 (0.9306358): g.41.11\|d.58.7 (0.6763006) | 1 |
| Fusion/Fission | 0 | 2 a.118.19 (0.6763006) -> b.131.1 (0.7630058): b.131.1\|a.118.19 (0.7109827) d.223.1 (0.6820809) -> d.144.1 (0.9710982): d.144.1\|d.223.1 (0.6936416) | 2 |
| Sum total | 0 | 4 | 4 |
| 5b) Single domains and domains from domain repeats in multidomains with repeats (777 /\ 3 = 3 parents with 3 common) | | | |
|  | Arcs with part of repeat match | Arcs with complete match | Arcs Total |
| Contemporaneous: Fusion | 0 | 0 | 0 |
| Fission | 0 | 0 | 0 |
| Fusion/Fission | 0 | 0 | 0 |
| Diachronic: Fusion | 1 b.23.2 (0.6820809) -> d.82.1 (0.7283237): b.23.2&\|d.82.1 (0.7283237) | 0 | 1 |
| Fission | 1 b.23.2 (0.6820809) -> a.86.1 (0.7630058): a.86.1\|b.23.2& (0.6763006) | 0 | 1 |
| Fusion/Fission | 1 d.223.1 (0.6820809) -> d.144.1 (0.9710982): d.144.1\|d.223.1& (0.8439307) | 0 | 1 |
| Sum total | 3 | 0 | 3 |
| 6a) Single domains and domains from multidomains with no repeats in multidomains with no repeats (3,483 /\ 248 = 247 parents with 247 common) | | | |
|  | Arcs with part of repeat match | Arcs with complete match | Arcs Total |
| Contemporaneous: Fusion | 0 | 6 | 6 |
| Fission | 0 | 0 | 0 |
| Fusion/Fission | 0 | 4 | 4 |
| Diachronic: Fusion | 0 | 135 c.37.1 (0.0000000) -> a.36.1 (0.2716763): a.24.13\|c.37.1\|a.36.1 (0.2716763) d.14.1 (0.1791908) -> a.4.9 (0.3352601): d.14.1\|d.101.1\|a.4.9\|d.14.1\|d.101.1\|d.51.1\|b.40.4 (0.3352601) c.26.2 (0.1271676) -> d.229.1 (0.4104046): c.26.2\|d.229.1\|b.153.1 (0.4104046) | 135 |
| Fission | 0 | 83 b.105.1 (0.3815029) -> e.3.1 (0.3872832): e.3.1\|b.105.1 (0.3815029) a.108.1 (0.3352601) -> d.45.1 (0.4624277): a.108.1\|d.45.1 (0.3352601) a.118.5 (0.5086705) -> d.2.1 (0.6647399): a.118.5\|d.2.1 (0.5086705) | 83 |
| Fusion/Fission | 0 | 93 g.81.1 (0.3641618) -> d.193.1 (0.6820809): d.193.1\|g.81.1\|c.51.5 (0.6763006) a.5.10 (0.4219653) -> d.79.4 (0.6878613): d.284.1\|a.5.10\|d.79.4\|d.139.1 (0.6705202) a.5.10 (0.4219653) -> d.79.4 (0.6878613): a.5.10\|d.79.4\|d.139.1\|d.79.4 (0.6763006) | 93 |
| Sum total | 0 | 321 | 321 |
| 6b) Single domains and domains from multidomains with no repeats in multidomains with repeats (777 /\ 16 = 16 parents with 16 common) | | | |
|  | Arcs with part of repeat match | Arcs with complete match | Arcs Total |
| Contemporaneous: Fusion | 0 | 0 | 0 |
| Fission | 0 | 0 | 0 |
| Fusion/Fission | 0 | 0 | 0 |
| Diachronic: Fusion | 3 c.2.1 (0.0057803) -> d.58.39 (0.4450867): d.58.39\|c.2.1& (0.6763006) c.37.1 (0.0000000) -> a.2.9 (0.6878613): c.37.1&\|a.2.9 (0.6994219) a.142.1 (0.5722544) -> b.35.2 (0.6820809): b.35.2\|a.142.1& (0.8208092) | 2 e.3.1 (0.3872832) -> d.175.1 (0.6936416): d.175.1\|e.3.1\|d.11.1& (0.7052023) b.149.1 (0.6763006) -> b.71.1 (0.7052023): c.1.8\|b.71.1\|b.149.1\|b.18.1& (0.7283237) | 5 |
| Fission | 1 h.1.6 (0.6820809) -> g.3.11 (0.8034682): c.62.1\|g.3.11&\|h.1.6 (0.6763006) | 1 h.1.6 (0.6820809) -> c.62.1 (0.7976879): c.62.1\|g.3.11&\|c.62.1\|h.1.6 (0.6820809) | 2 |
| Fusion/Fission | 10 a.5.10 (0.4219653) -> d.79.4 (0.6878613): d.284.1\|a.5.10\|d.79.4&\|d.139.1\|c.23.16 (0.6820809) a.9.1 (0.3930636) -> c.43.1 (0.7398844): b.84.1\|a.9.1&\|c.43.1 (0.6820809) a.9.1 (0.3930636) -> c.43.1 (0.7398844): a.9.1&\|c.43.1 (0.6820809) | 6 c.55.3 (0.0809249) -> b.48.1 (0.6763006): a.4.1&\|c.55.3\|b.48.1 (0.6705202) b.149.1 (0.6763006) -> b.71.1 (0.7052023): b.71.1\|b.149.1\|b.18.1& (0.6994219) a.9.1 (0.3930636) -> c.43.1 (0.7398844): b.84.1&\|a.9.1\|c.43.1 (0.4739884) | 16 |
| Sum total | 14 | 9 | 23 |
| 7) Single domains and domains from multidomains with repeats in multidomains with repeats (777 /\ 13 = 12 parents with 12 common) | | | |
|  | Arcs with part of repeat match | Arcs with complete match | Arcs Total |
| Contemporaneous: Fusion | 0 | 0 | 0 |
| Fission | 0 | 0 | 0 |
| Fusion/Fission | 0 | 0 | 0 |
| Diachronic: Fusion | 6 c.37.1 (0.0000000) -> b.34.18 (0.4913295): c.37.1&\|b.34.18\|c.37.1&\|d.315.1 (0.4913295) c.37.1 (0.0000000) -> b.34.18 (0.4913295): c.37.1&\|b.34.18 (0.6763006) a.3.1 (0.2890173) -> b.61.4 (0.6878613): a.3.1&\|b.61.4\|b.1.18& (0.6878613) | 4 b.40.4 (0.0520231) -> d.202.1 (0.4219653): d.202.1\|b.40.4\|d.52.3&\|a.60.4& (0.4219653) c.37.1 (0.0000000) -> b.34.18 (0.4913295): c.37.1\|b.34.18\|c.37.1&\|d.315.1 (0.6936416) b.40.4 (0.0520231) -> d.202.1 (0.4219653): d.202.1\|b.40.4\|d.52.3&\|a.60.4 (0.7167630) | 10 |
| Fission | 2 g.41.13 (0.6820809) -> d.37.1 (0.6936416): d.37.1&\|g.41.13 (0.6820809) b.61.4 (0.6878613) -> b.1.18 (0.8728324): a.3.1&\|b.61.4\|b.1.18& (0.6878613) | 1 a.7.15 (0.4450867) -> d.322.1 (0.8092486): a.7.15\|d.322.1\|d.136.1& (0.4450867) | 3 |
| Fusion/Fission | 0 | 0 | 0 |
| Sum total | 8 | 5 | 13 |
| 8a) Domains from both domain repeats and multidomains with no repeats in multidomains with no repeats (4 /\ 248 = 0 parents with 0 common) | | | |
|  | Arcs with part of repeat match | Arcs with complete match | Arcs Total |
| Contemporaneous: Fusion | 0 | 0 | 0 |
| Fission | 0 | 0 | 0 |
| Fusion/Fission | 0 | 0 | 0 |
| Diachronic: Fusion | 0 | 0 | 0 |
| Fission | 0 | 0 | 0 |
| Fusion/Fission | 0 | 0 | 0 |
| Sum total | 0 | 0 | 0 |
| 8b) Domains from both domain repeats and multidomains with no repeats in multidomains with repeats (3 /\ 16 = 0 parents with 0 common) | | | |
|  | Arcs with part of repeat match | Arcs with complete match | Arcs Total |
| Contemporaneous: Fusion | 0 | 0 | 0 |
| Fission | 0 | 0 | 0 |
| Fusion/Fission | 0 | 0 | 0 |
| Diachronic: Fusion | 0 | 0 | 0 |
| Fission | 0 | 0 | 0 |
| Fusion/Fission | 0 | 0 | 0 |
| Sum total | 0 | 0 | 0 |
| 9) Domains from both domain repeats and multidomains with repeats in multidomains with repeats (3 /\ 13 = 0 parents with 0 common) | | | |
|  | Arcs with part of repeat match | Arcs with complete match | Arcs Total |
| Contemporaneous: Fusion | 0 | 0 | 0 |
| Fission | 0 | 0 | 0 |
| Fusion/Fission | 0 | 0 | 0 |
| Diachronic: Fusion | 0 | 0 | 0 |
| Fission | 0 | 0 | 0 |
| Fusion/Fission | 0 | 0 | 0 |
| Sum total | 0 | 0 | 0 |
| 10) Domains from both multidomains with no repeats and multidomains with repeats in multidomains with repeats (16 /\ 13 = 1 parents with 1 common) | | | |
|  | Arcs with part of repeat match | Arcs with complete match | Arcs Total |
| Contemporaneous: Fusion | 0 | 0 | 0 |
| Fission | 0 | 0 | 0 |
| Fusion/Fission | 0 | 0 | 0 |
| Diachronic: Fusion | 0 | 0 | 0 |
| Fission | 0 | 0 | 0 |
| Fusion/Fission | 0 | 0 | 0 |
| Sum total | 0 | 0 | 0 |
| Grand total | 855 | 5,011 | 5,866 |
| 11) Domain Categories | | | |
| Donors | 270 - c.37.1 (0.0000000); e.7.1 (0.1329480); a.128.1 (0.1387283) | | |
| Acceptors | 382 - c.56.2 (0.1907514); c.12.1 (0.2312139); c.18.1 (0.2369942) | | |
| Donors/Acceptors | 473 - c.2.1 (0.0057803); c.67.1 (0.0115607); c.66.1 (0.0173410) | | |
| Peers | 22 - c.83.1 (0.3236994); c.114.1 (0.3583815); f.25.1 (0.3641618) | | |
| Noncombinables | 496 - g.41.8 (0.1965318); f.41.1 (0.2312139); a.75.1 (0.2427746) | | |

Supplementary Table 6: Categorization of domains and multidomains into four subgroups and the extraction of unique spliced domains from the subgroups. Four subgroups of domain organization were defined based on structural differences: single domains, domain repeats, multidomains with no repeats and multidomains with repeats. Unique spliced domains were extracted from these four subgroups, in that order. The single domains subgroup was included as it is. The repeat domains in domain repeats and multidomains with repeats subgroups were truncated. Finally, all the multidomains were spliced and unique domains were included in the data set of 1,643 single and spliced domains. The above order of subgroups for computation of unique spliced domains reduced redundancy by avoiding overlap with the prior subgroup of less structural complexity. An implication of this scheme of extracting a unique data set was that 125 spliced domains borrowed ages of their parent multidomains, out of which 48 had multiple parent multidomains and were assigned the age of the ancient most parent multidomain. Given the above defined order of structural groups for extraction of the SPX domain set and the scheme for assignment of ages, eight of the 169 unique age slivers of the original 6,162 domains/multidomains data set were left unassigned in the SPX’s and SPAX’s 1,643 domains data set. The eight missing nd values from the timeline were 0.046, 0.087, 0.168, 0.173, 0.416, 0.538, 0.618 and 0.954. The algorithm designed to generate unique spliced domains had a reduced time complexity of O(n) due to incorporation of the structured programming functions provided by PHP 4. The space complexity of the algorithm was <= 64MB and it required ~0.316 seconds to run. Computation of spliced domains based on the domain repeats section of the multidomains with repeats subgroup separately was eluded due to lower significance and overcomplication of the resultant summary data. The scheme of extracting unique spliced domains based on increasing level of structural complexity ensured consistency with the original data set of protein domain organization, specifically in terms of age assignments of single and spliced domains.

| 1) Single domains (all included) | 1,513 |
| --- | --- |
|  |  |
| 2) Domains from domain repeats | |
| 2a) Domain repeats (truncated to domains) | 387 |
| 2b) Excluding domains from 2a) common with 1) | 5 |
|  |  |
| 3) Domains from multidomains with no repeats | |
| 3a) Multidomains with no repeats (original set) | 3,484 |
| 3b) Domains spliced from 3a) | 8,418 |
| 3c) Unique domains in 3b) | 1,122 |
| 3d) Excluding domains from 3c) common with 1) | 119 |
| 3e) Excluding domains from 3d) common with 2b) | 116 |
| 3f) Domains in 3e) reassigned age most ancient across multiple occurrences in 3a) | 46 |
|  |  |
| 4) Domains from multidomains with repeats | |
| 4a) Multidomains with repeats (original set) | 778 |
| 4b) Domains/domain repeats truncated and spliced from 4a) | 2,280 |
| 4c) Unique domains in 4b) | 442 |
| 4d) Excluding domains from 4c) common with 1) | 21 |
| 4e) Excluding domains from 4d) common with 2b) | 19 |
| 4f) Excluding domains from 4e) common with 3e) | 9 |
| 4g) Domains in 3f) reassigned age most ancient across multiple occurrences in 4a) | 2 |

**Supplementary Videos**

**Supplementary** **Video 1**. Simulation of evolutionary growth of the SPX network of domain organization from ancient to modern times of the protein world, March 18, 2021.

**Supplementary** **Video 2**. Simulation of *waterfall* model of the SPX network based on the linear (incremented by 1) progression of networks with nodes representing connectivity higher than or equal to various (0 to 100) percentiles of combined outdegree and indegree at Age 1, March 18, 2021.

**Supplementary** **Video 3**. Simulation of pairwise *NG_age_* modularity of the SPX network through progression of heat maps and dendrograms over the timeline, March 18, 2021.

**Supplementary Files**

**Supplementary** **File 1**. Data matrix used to build the phylogenomic tree of architectures. Genome identifiers are described in Table S1 of ref.^20^, March 18, 2021.

**Supplementary** **File 2**. Most parsimonious phylogenomic tree of domain architectures, March 18, 2021.

**Supplementary** **File 3**. Most parsimonious phylogenomic tree of domain architectures, with taxa labeled with SCOP *ccs* identifiers, March 18, 2021.
